# Effect of Coordination Environment and Electronic Coupling on Redox Entropy in a Family of Dinuclear Complexes

**DOI:** 10.1021/acselectrochem.4c00186

**Published:** 2025-02-18

**Authors:** Daniela Carmona-Pérez, Meiqin Gao, Samantha Andes, William W. Brennessel, Agnes E. Thorarinsdottir

**Affiliations:** Department of Chemistry, University of Rochester, Rochester, New York 14627, United States

**Keywords:** variable-temperature electrochemistry, temperature coefficient, redox entropy, electronic coupling

## Abstract

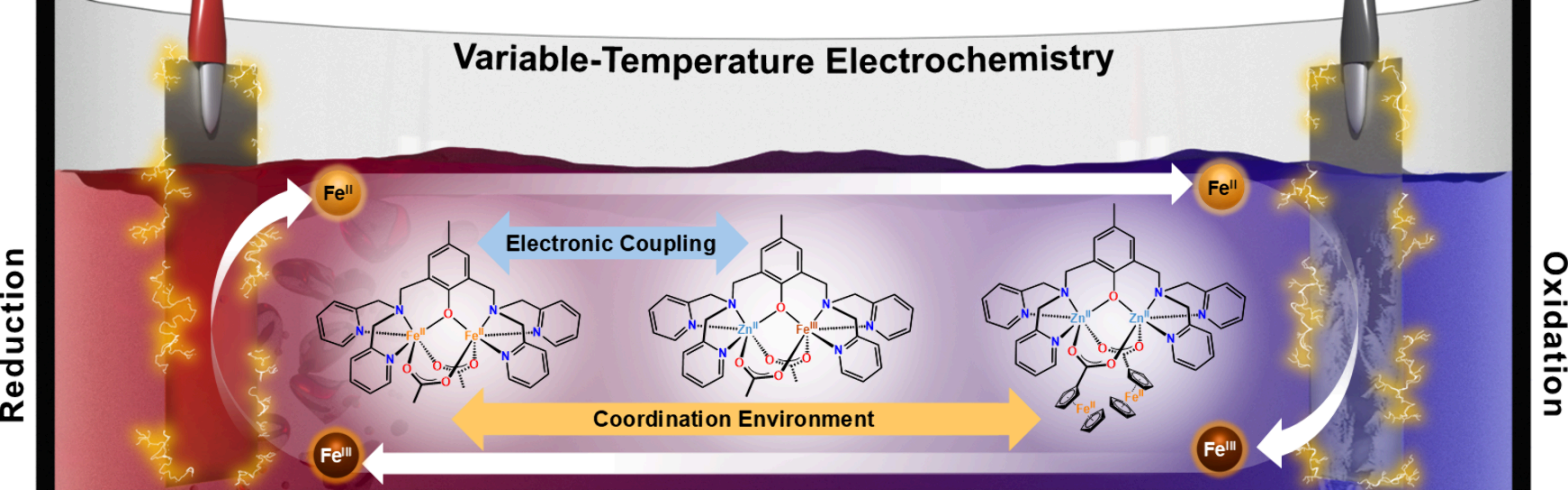

The elucidation of factors that govern the temperature sensitivity of the electrochemical potential is essential to the development of electrochemical systems with target properties. Toward this end, we report a series of isostructural homo- and heterometallic M_2_ (M = Fe^II^, Fe^III^, Zn^II^) complexes supported by a phenoxo-centered tetrapyridyl ligand and ancillary carboxylate ligands that enables independent change in (i) charge, (ii) coordination environment of the redox-active center(s), and (iii) electronic coupling strength between redox centers. Variable-temperature electrochemical analysis of the series reveals the temperature coefficient for Fe-based redox couples to be highly dependent on the coordination environment of the redox-active center(s), with Fe centers in a pseudo-octahedral [FeN_3_O_3_] coordination environment affording a 2-fold greater temperature coefficient for the Fe^III^/Fe^II^ redox couple than those in ancillary ferrocenyl groups. In contrast, identical temperature coefficients for the Fe^III^/Fe^II^ redox event in Fe_2_ and FeZn complexes establish electronic coupling strength to have a minimal impact on the temperature dependence of the Fe-based redox couple. Taken together, these results provide important insights for the design of molecular compounds with target redox properties, and they provide the first examination of how electronic coupling influences the temperature dependence of the redox potential and the associated redox entropy in molecular compounds.

## Introduction

Charge-transfer reactions underpin myriad technologies relying on chemical, electrochemical, and photochemical processes.^1−4^ For electrochemical processes in particular, the kinetics and thermodynamics of electron-transfer reactions determine the efficacy of a given redox system toward applications in energy storage and conversion devices, electrocatalysis, and electrochemical sensing.^5−7^ As such, a complete understanding of the kinetics and thermodynamics of electron-transfer processes is essential to inform the design of electrochemical systems with target properties for specific applications in energy and sustainability.

Toward developing understanding of the thermodynamics of electron-transfer reactions, variable-temperature electrochemical analysis of molecular compounds is one attractive approach owing to the inherent linear relationship between the redox reaction entropy (Δ*S*_redox_) and the temperature dependence of the electrochemical potential (∂*E*/∂*T*)—the so-called temperature coefficient (α)—of redox-active molecules in solution (eq 1).
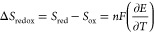
1The entropy change in a redox reaction is caused by the difference in partial molar entropies of the reduced (*S*_red_) and oxidized (*S*_ox_) species, originating from structural (bond lengths, bond angles) and physical (spin state) changes in the redox species and effects from their interactions with surrounding solvent molecules and electrolyte ions.^8−10^ Indeed, variable-temperature electrochemistry has found significant success in determining Δ*S*_redox_ of various one-electron-transfer reactions in mononuclear transition metal complexes^8,10−28^ and more recently of one- and two-electron-transfer reactions in polyoxometalates.^29,30^ Such studies have provided insights into the influence of charge and solution environment on Δ*S*_redox_. Moreover, compounds that exhibit a large absolute temperature coefficient are attractive for use in liquid-phase thermoelectrochemical cells (also known as thermocells or thermogalvanic cells) for converting low-grade waste heat into electricity,^9,10,18−22,24,26−29^ as well as for electrochemical sensing of temperature.^16,17,25^

While molecules and molecular clusters offer an ideal platform for understanding the thermodynamics of electron-transfer reactions by virtue of their excellent tunability through ligand and structure design, there is a dearth of studies that probe electronic effects on Δ*S*_redox_ for molecular compounds. As such, there is a significant interest in understanding how electronic effects of metal substitution influence the temperature dependence of the electrochemical potential for isostructural molecular compounds. Combining such electronic studies with structural investigations would enhance our understanding of the factors that dictate the temperature responsiveness of the electrochemical potential and thus facilitate the design of compounds with optimal temperature coefficients and thermodynamic properties for desired applications.

Toward this end, we set out to design a system in which the identity of the redox-active center(s) can be precisely tuned with minimal structural perturbation and with independent alteration of the overall charge of the system. Dinuclear transition metal complexes supported by a dinucleating tetrapyridyl ligand and ancillary carboxylate ligands satisfy these criteria. Indeed, various homo- and heterometallic dinuclear complexes have been synthesized using this platform,^31−39^ highlighting the potential of this system to elucidate the impact of coordination environment and electronic coupling on the temperature dependence of the electrochemical potential. Accordingly, we herein report a series of homo- and heterometallic M_2_ (M = Fe^II^, Fe^III^, Zn^II^) complexes bearing ancillary acetate or ferrocenecarboxylate ligands and demonstrate that the coordination environment around the redox-active center(s) has profound effects on Δ*S*_redox_ and the temperature dependence of the electrochemical potential, while electronic coupling strength is shown to have a minimal impact. These studies underscore that not only charge, but also proper redox center design is a key element to consider toward optimizing the temperature sensitivity of molecular redox reactions.

## Experimental Section

### General Considerations

Unless otherwise specified, the manipulations described below were carried out under ambient atmosphere and temperature. Air- and water-free manipulations were performed under a dry dinitrogen atmosphere in a MBraun UNIlab pro glovebox. Glassware was oven-dried at 125 °C for at least 4 h and allowed to cool in an evacuated antechamber prior to use in the glovebox. Acetonitrile (MeCN), dichloromethane (CH_2_Cl_2_), diethyl ether (Et_2_O), methanol (MeOH), and tetrahydrofuran (THF) solvents used inside the glovebox were dried using a commercial solvent purification system from Pure Process Technology and stored over 3 Å molecular sieves prior to use. MeCN, CH_2_Cl_2_, Et_2_O, 1,2-dimethoxyethane (DME), hexanes, MeOH, and triethylamine solvents used outside the glovebox were purchased from Sigma–Aldrich and Fisher Scientific. Ultrapure water (18.2 MΩ cm) was obtained from an Arium Mini water purification system from Sartorius. Deuterated solvents were purchased from Cambridge Isotope Laboratories. (^*n*^Bu_4_N)(PF_6_) used for electrochemical measurements was purchased from A2B Chem LLC and recrystallized two times from ethanol and dried under vacuum for at least 12 h prior to use. Anhydrous iron(II) acetate (Fe(OAc)_2_) was obtained as a generous gift from the Barnett Laboratory at the University of Rochester, synthesized following a modification of a published protocol.^40^ All other reagents were purchased from commercial vendors and used without further purification. Experimental details on the synthesis and characterization of ligands and organic precursors are provided in the Supporting Information.^38,41,42^

### Syntheses of Dinuclear Complexes

#### Synthesis of [(BPMP)Fe_2_(OAc)_2_](PF_6_) (**1**)

**Figure 1:**
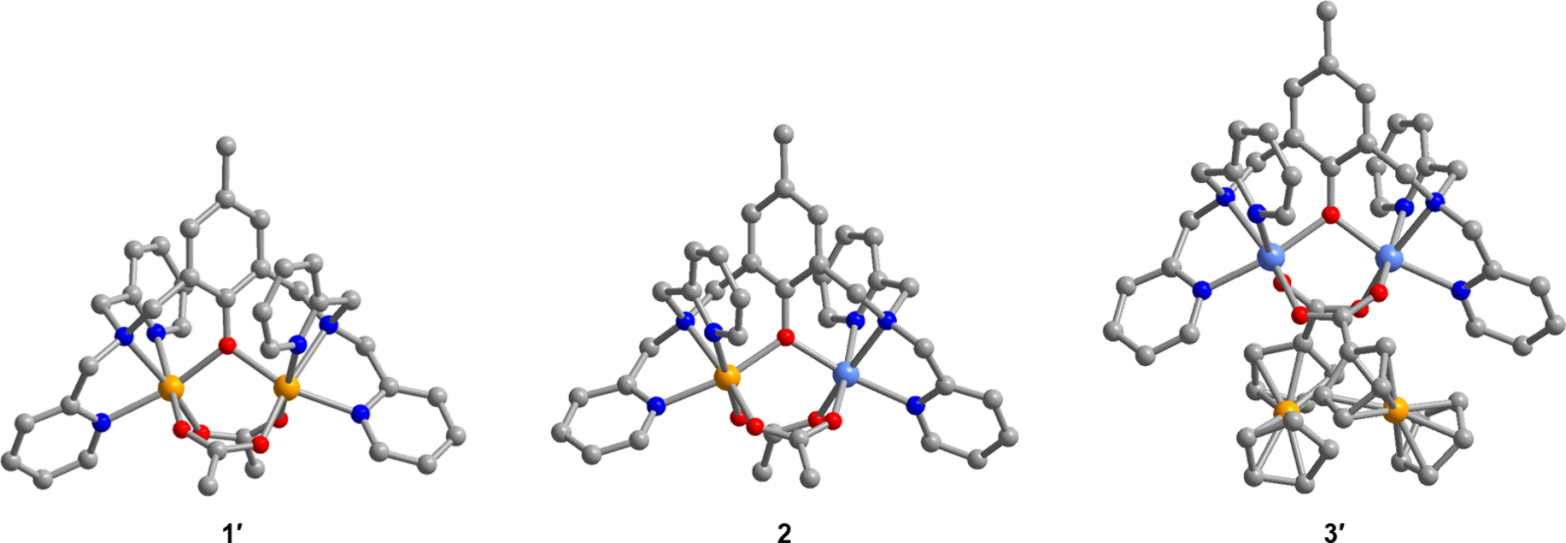
Crystal structures of the cationic complexes [(BPMP)Fe_2_(OAc)_2_]^+^, [(BPMP)FeZn(OAc)_2_]^2+^, and [(BPMP)Zn_2_(FcCOO)_2_]^+^, as observed in **1′**, **2**, and **3′**, respectively. Light blue, orange, red, blue, and gray spheres represent Zn, Fe, O, N, and C atoms, respectively; H atoms are omitted for clarity.

This compound was synthesized following a modified literature procedure.^38^ Inside the glovebox, a colorless solution of Fe(OAc)_2_ (143 mg, 0.82 mmol) in MeCN (2 mL) was added dropwise to a stirring yellow solution of HBPMP (197 mg, 0.37 mmol) in MeCN (3 mL) to give a dark yellow brown solution. After being stirred at 25 °C for 15 min, a colorless solution of NaOAc (71 mg, 0.87 mmol) in MeCN (2 mL) was added dropwise, and the resulting orange brown solution was stirred for 1 h. A colorless solution of (NH_4_)(PF_6_) (75 mg, 0.46 mmol) in MeCN (2 mL) was then added dropwise and the orange brown solution was stirred for 30 min. The reaction solution was filtered through a Kimwipe plug and the solvent was removed under reduced pressure. Diffusion of Et_2_O vapor into a concentrated solution of the red orange residue in CH_2_Cl_2_ (6 mL) afforded an orange solid that was washed with THF (2 × 10 mL) and dried in the glovebox atmosphere for 15 min. The orange solid was recrystallized two times using diffusion of Et_2_O vapor into a concentrated solution of the solid in CH_2_Cl_2_ (6 mL) to give a crystalline orange solid that was washed with Et_2_O (2 × 5 mL) and dried under reduced pressure for 12 h to give **1** (135 mg, 40%) as an orange crystalline solid. Anal. Calcd for C_37_H_39_F_6_Fe_2_N_6_O_5_P: C, 49.14; H, 4.35; N, 9.29%. Found: C, 49.44; H, 4.49; N, 9.13%. UV–visible absorption spectrum (0.1 mM; MeCN, 22–23 °C): 256 nm (14680 M^–1^ cm^–1^), 306 nm (3840 M^–1^ cm^–1^), 441 nm (2350 M^–1^ cm^–1^). NIR absorption spectrum (3.0 mM; MeCN, 22–23 °C): ∼890 nm (28 M^–1^ cm^–1^), ∼1040 nm (24 M^–1^ cm^–1^). Solution magnetic susceptibility (6.2 mM; MeCN, 295.2 K): 6.95(4) cm^3^ K mol^–1^. Slow diffusion of Et_2_O vapor into a concentrated solution of **1** in CH_2_Cl_2_ afforded orange plate-shaped crystals of [(BPMP)Fe_2_(OAc)_2_](PF_6_)·solvent (**1′**) suitable for single-crystal X-ray diffraction analysis (Figure 1, left, Figure S1). Note that the term “solvent” in the formula above denotes a combination of crystallographically disordered CH_2_Cl_2_ and Et_2_O molecules (vide infra).

#### Synthesis of [(BPMP)Fe_2_(OAc)_2_](PF_6_)_2_·0.2(NH_4_)(PF_6_) (**1-ox**)

A colorless solution of (NH_4_)(PF_6_) (99 mg, 0.61 mmol) in MeCN (5 mL) was added dropwise to a stirring orange solution of [(BPMP)Fe_2_(OAc)_2_](PF_6_) (177 mg, 0.20 mmol) in MeCN (5 mL) to give a black solution. This solution was left undisturbed for 16 h and Et_2_O (20 mL) was added to give a grey solid. The grey solid was collected by filtration and washed with MeOH (10 mL) and Et_2_O (20 mL). Diffusion of Et_2_O vapor into a concentrated solution of the grey solid in MeCN (2 mL) afforded a crystalline black solid that was washed with Et_2_O (10 mL) and dried under reduced pressure for 6 h to give **1-ox** (107 mg, 51%) as a black crystalline solid. Anal. Calcd for C_37_H_39.8_F_13.2_Fe_2_N_6.2_O_5_P_2.2_: C, 41.07; H, 3.71; N, 8.03%. Found: C, 41.03; H, 3.73; N, 8.49%. ESI–MS (*m*/*z*): Calcd for C_37_H_39_Fe_2_N_6_O_5_ ([(BPMP)Fe_2_(OAc)_2_]^2+^), 379.6; found, 379.8. UV–visible absorption spectrum (0.1 mM; MeCN, 22–23 °C): 254 nm (18820 M^–1^ cm^–1^), ∼295 nm (7410 M^–1^ cm^–1^), ∼380 nm (2450 M^–1^ cm^–1^), ∼545 nm (880 M^–1^ cm^–1^). UV–visible absorption spectrum (solid, 22 °C): 255 nm, 296 nm, ∼380 nm, ∼580 nm. NIR absorption spectrum (2.9 mM; MeCN, 22–23 °C): ∼1300 nm (270 M^–1^ cm^–1^). Solution magnetic susceptibility (5.5 mM; MeCN, 295.2 K): 8.50(4) cm^3^ K mol^–1^. Slow diffusion of DME vapor into a concentrated solution of **1-ox** in CH_2_Cl_2_ afforded dark green plate-shaped crystals of [(BPMP)Fe_2_(OAc)_2_](PF_6_)_2_·DME (**1-ox′**) suitable for single-crystal X-ray diffraction analysis (Figures S2 and S3).

#### Synthesis of [(BPMP)FeZn(OAc)_2_](PF_6_)_2_ (**2**)

This compound was synthesized following a modified literature procedure.^31^ An orange solution of Fe(NO_3_)_3_·9H_2_O (84 mg, 0.21 mmol) in MeOH (5 mL) was added dropwise to a stirring yellow solution of HBPMP (110 mg, 0.21 mmol) in MeOH (5 mL) to give a dark blue solution. After being stirred at 25 °C for 15 min, a colorless solution of ZnBr_2_ (47 mg, 0.21 mmol) in MeOH (2 mL) was added dropwise and the dark green solution was stirred for 15 min. A colorless solution of Zn(OAc)_2_·2H_2_O (136 mg, 0.62 mmol) in MeOH (2 mL) was then added and the resulting purple solution was stirred for 3 h. Subsequently, a colorless solution of (NH_4_)(PF_6_) (340 mg, 2.1 mmol) in MeOH (3 mL) was added dropwise and the solution was cooled to 5 °C in the refrigerator for 16 h to afford a purple solid. The purple solid was collected by filtration and washed with MeOH (20 mL) and Et_2_O (40 mL). Diffusion of Et_2_O vapor into a concentrated solution of the purple solid in MeCN (2 mL) afforded a crystalline purple solid that was washed with Et_2_O (10 mL) and dried under reduced pressure for 6 h to give **2** (124 mg, 56%) as a purple crystalline solid. Anal. Calcd for C_37_H_39_F_12_FeN_6_O_5_P_2_Zn: C, 41.97; H, 3.71; N, 7.94%. Found: C, 41.80; H, 3.70; N, 7.85%. ESI–MS (*m*/*z*): Calcd for C_37_H_39_F_6_FeN_6_O_5_PZn ([(BPMP)FeZn(OAc)_2_(PF_6_)]^+^), 912.1; found, 912.2. ICP–MS: molar ratio Zn:Fe = 1.00(1). UV–visible absorption spectrum (0.1 mM; MeCN, 22–23 °C): 255 nm (17730 M^–1^ cm^–1^), ∼295 nm (6400 M^–1^ cm^–1^), ∼360 nm (1900 M^–1^ cm^–1^), 560 nm (950 M^–1^ cm^–1^). UV–visible absorption spectrum (solid, 22 °C): 257 nm, 296 nm, 565 nm. Solution magnetic susceptibility (10.0 mM; MeCN, 296.2 K): 4.38(1) cm^3^ K mol^–1^. Slow diffusion of Et_2_O vapor into a concentrated solution of **2** in MeCN afforded purple plate-shaped crystals of **2** suitable for single-crystal X-ray diffraction analysis (Figure 1, center, Figure S4).

#### Synthesis of [(BPMP)Zn_2_(FcCOO)_2_](PF_6_)_0.97_(OTf)_0.03_·0.2NaPF_6_ (**3**)

This compound was synthesized following a modified literature procedure.^36^ Inside the glovebox, a colorless solution of NaOMe (8.6 mg, 0.16 mmol) in MeOH (1 mL) was added dropwise to a stirring yellow solution of HBPMP (77 mg, 0.15 mmol) in MeOH (3 mL) at 50 °C to give a dark yellow brown solution. After being stirred at 50 °C for 15 min, a colorless solution of Zn(OTf)_2_ (111 mg, 0.31 mmol) in MeOH (1 mL) was added dropwise and the dark yellow brown solution was stirred for 15 min. Subsequently, an orange solution of (^*n*^Bu_4_N)(FcCOO) (133 mg, 0.28 mmol) in MeOH (1 mL) was added dropwise and the resulting orange solution was stirred at 50 °C for 1 h. This orange solution was filtered while hot through a PTFE syringe filter. A white suspension of NaPF_6_ (0.84 mg, 0.50 mmol) in MeOH (3 mL) was added to the orange filtrate and the resulting mixture was stirred at 50 °C for 30 min. The orange turbid solution was cooled to 5 °C in the refrigerator for 16 h to afford a yellow solid. The yellow solid was collected by filtration in air and washed with MeOH (20 mL) and Et_2_O (40 mL). Slow evaporation of a concentrated solution of the yellow solid in MeCN (5 mL) afforded crystalline yellow solid that was washed with Et_2_O (10 mL) and dried under reduced pressure for 6 h to give **3** (96 mg, 51%) as a yellow crystalline solid. Anal. Calcd for C_55.03_H_51_F_7.11_Fe_2_N_6_Na_0.2_O_5.09_P_1.17_S_0.03_Zn_2_: C, 50.95; H, 3.96; N, 6.48%. Found: C, 50.58; H, 3.86; N, 6.45%. ESI–MS (*m*/*z*): Calcd for C_55_H_51_Fe_2_N_6_O_5_Zn_2_ ([(BPMP)Zn_2_(FcCOO)_2_]^+^), 1117.1; found, 1117.2. ^1^H NMR (400 MHz, (CD_3_)_2_SO, 22 °C): δ 8.95 (d, 2H), 8.26 (d, 2H), 8.14 (t, 2H), 7.76 (d, 2H), 7.66 (t, 2H), 7.45 (t, 2H), 7.10 (t, 2H), 6.68 (d, 2H), 6.50 (s, 2H), 4.59 (d, 2H), 4.42 (d, 4H), 4.25 (m, 6H), 4.06 (s, 10H), 3.93 (d, 2H), 3.69 (d, 2H), 3.51 (d, 2H), 3.16 (d, 2H),1.96 (s, 3H) (Figure S5). ^19^F{^1^H} NMR (376 MHz, (CD_3_)_2_SO, 22 °C): δ −70.02 (d, ^1^*J*_PF_ = 711 Hz), −77.65 (Figure S6). UV–visible absorption spectrum (0.1 mM; MeCN, 22–23 °C): 255 nm (19570 M^–1^ cm^–1^), 306 nm (4990 M^–1^ cm^–1^), 444 nm (350 M^–1^ cm^–1^). UV–visible absorption spectrum (solid, 22 °C): ∼260 nm, ∼319 nm, 443 nm. Slow evaporation of a concentrated MeCN solution of **3** afforded yellow plate-shaped crystals of [(BPMP)Zn_2_(FcCOO)_2_](PF_6_)_0.78_(OTf)_0.22_·2MeCN (**3′**) suitable for single-crystal X-ray diffraction analysis (Figure 1, right, Figure S7).

### X-ray Structure Determination

Single crystals of **1′**, **1-ox′**, **2**, and **3′** were placed onto a nylon loop and mounted on a Rigaku XtaLAB Synergy-S Dualflex diffractometer equipped with a HyPix-6000HE HPC area detector for data collection at 100 K. A preliminary set of cell constants and an orientation matrix were calculated from a small sampling of reflections.^43^ For each crystal, a short pre-experiment was run, from which an optimal data collection strategy was determined. The full data collections were carried out using a PhotonJet Cu X-ray source. Raw data were integrated and corrected for Lorentz and polarization effects with CrysAlis^Pro^.^43^ Absorption corrections were applied using the multiscan method within CrysAlis^Pro^.^43^ Space group assignments were determined based on intensity statistics. Structures were solved using direct methods in SHELXT^44^ and refined using SHELXL^45^ operated within the OLEX2 interface.^46^ Most or all non-hydrogen atoms were assigned from the direct methods structure solution. Full-matrix least squares and difference Fourier cycles were then performed which located any remaining non-hydrogen atoms based on electron density and proposed molecular formula. All hydrogen atoms were placed at calculated positions using suitable riding models and refined using isotropic displacement parameters derived from their parent atoms. Thermal parameters for all non-hydrogen atoms were refined anisotropically.

In the crystal structure of **1′**, reflection contributions from highly disordered solvent molecules, located in channels along [100], were fixed and added to the calculated structure factors using the SQUEEZE routine of the program Platon.^47^ A void volume of 316 Å^3^, with 70 electrons, was estimated per unit cell and ascribed to a combination of CH_2_Cl_2_ and Et_2_O molecules. Due to this disorder, the nomenclature of the single crystals of **1′** is noted as [(BPMP)Fe_2_(OAc)_2_](PF_6_)·solvent. Thus, all calculated quantities that derive from the molecular formula are known to be inaccurate. The intensity data of **1-ox′** were integrated according to non-merohedral twin law [−1 0 0/0–1 0 / −0.536 0.005 1], a 180° rotation around reciprocal lattice [001]. There were 4032 unique isolated reflections in the first component, 4058 unique isolated reflections in the second component, and 7800 unique overlapping reflections. Without twin modeling, *R*_1_ (strong data) refined to 0.211. The mass ratio of the two components refined to 0.5030(11):0.4970(11). In the crystal structure of **2**, there is one Fe and one Zn atom between the two metal sites, and each site contains a mixture of the two atom types in the following ratio: Fe1:Zn2, 0.64:0.36 and Fe2:Zn1, 0.36:0.64. In the crystal structure of **3′**, the anion is modeled as a disordered mixture of (PF_6_)^−^ and (OTf)^−^ in a ratio of 0.78:0.22. Crystallographic data for these compounds and the details of data collection are listed in Table S1.^48^ Selected interatomic distances and angles for **1′**–**3′** and **1-ox′** are further provided in Tables 1 and S2, respectively.

**Table 1:** Selected Mean Interatomic Distances (Å) and Angles (deg) and Octahedral Distortion Parameter (*∑*_sum_) for the Cationic Complexes in **1′**, **2**, and **3′** at 100 K^a^

	**1′**	**2**	**3′**
M–O_phenoxo_	2.0573(9)	2.035(2)	2.0433(9)
M–O_carboxylate_	2.0939(7)	2.022(1)	2.0715(6)
M–N	2.2185(6)	2.155(1)	2.1930(6)
M···M^b^	3.3558(3)	3.4480(5)	3.3349(3)
M–O_phenoxo_–M	109.30(5)	115.80(9)	109.39(5)
O–C–O	125.5(2)	125.3(2)	126.2(2)
*trans-*O–M–N	165.43(3)	168.95(4)	165.63(3)
*∑*_sum_^c^	80.74(2)	68.75(2)	74.66(2)

a M denotes a Fe or a Zn atom.

b Intramolecular M···M distance.

c Octahedral distortion parameter (∑) = absolute deviation from 90° of each 12 *cis* angle in [MN_3_O_3_].

### Electrochemical Measurements

All electrochemical experiments were carried out under a dry dinitrogen atmosphere in a MBraun UNIlab pro glovebox using CH Instruments 760E electrochemical workstation. Samples of complexes **1**, **1-ox**, **2**, and **3** (0.7–5.0 mM) were prepared in dry MeCN containing 0.1 M (^*n*^Bu_4_N)(PF_6_) supporting electrolyte. Unless otherwise specified, experiments were carried out using a 10 mL glass beaker or a 20 mL scintillation vial, a 3 mm diameter glassy carbon working electrode (CH Instruments, Inc.), a non-aqueous Ag/AgNO_3_ reference electrode (CH Instruments, Inc.) filled with a MeCN solution containing 0.01 M AgNO_3_ and 0.1 M (^*n*^Bu_4_N)(PF_6_), and a counter electrode composed of a platinum mesh (99.9%, 52 mesh woven from 0.1 mm diameter wire, Fisher Scientific) attached to a platinum wire (99.95%, 0.5 mm diameter, Fisher Scientific). Before use, glassy carbon working electrodes were polished with alumina powder (0.05 μm, Allied High Tech Products, Inc.) on a microfiber polishing cloth and platinum counter electrodes were cleaned by soaking in concentrated nitric acid followed by drying with a butane flame. All electrochemical glassware was washed with concentrated nitric acid (ACS Plus, 15.8 N, Fisher Scientific) and ultrapure water before experiments, and oven-dried for at least 4 h prior to use. Cyclic voltammetry (CV) measurements were conducted using a positive scan direction and a scan rate of 25–400 mV s^–1^. The second CV cycle is displayed in all cases. All potentials are referenced to the Ag/AgNO_3_ electrode. Drifts in the reference electrode potential were checked routinely using the Fc^+^/Fc redox couple (*E*_1/2_ = 0.090 V vs Ag/AgNO_3_). Uncompensated solution resistance (*R*_u_) was determined to be ∼9–44 Ω using the potential step method (amplitude of 50 mV) around the open-circuit potential (*E*_OCP_). Note, however, that all potentials are reported without applying *iR*_u_ compensation as identical CV traces were obtained with and without applying such compensation in manual mode (Figure S8). Negative currents correspond to anodic reactions (oxidation) and positive currents correspond to cathodic reactions (reduction). Independently prepared samples of each type were measured to ensure reproducibility. The provided data are representative examples.

### Diffusion Coefficient Determination

The diffusion coefficients associated with each redox process for complexes **1**, **1-ox**, **2**, and **3** were determined at room temperature (23–25 °C) from variable-scan-rate (25–400 mV s^–1^) CV measurements. The anodic and cathodic diffusion coefficients of each redox couple were separately quantified from the anodic and cathodic peak currents, respectively, using Randles–Ševčík analysis.^49,50^ Specifically, the diffusion coefficients were estimated using the slopes of the linear fits to the data in plots of peak current vs the square root of scan rate. As the redox couples of **1**, **1-ox**, **2**, and **3** are not strictly reversible, rather quasi-reversible, as the peak potential separation is >59.2 mV per electron transfer at 25 °C and increases slightly with scan rate in the 25–400 mV s^–1^ range, the diffusion coefficients were separately estimated using the Randles–Ševčík equations for a fully reversible redox couple (eq 2) and an irreversible redox couple (eq 3) at 25 °C:^49−52^
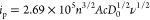
2
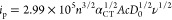
3In these equations, *i*_p_ is the peak current (anodic or cathodic), *n* is the number of electrons transferred in the given reaction, *A* is the geometric surface area of the working electrode (cm^2^), *c* is the concentration of the redox-active species in the bulk solution (mol cm^–3^), *D*_0_ is the diffusion coefficient (cm^2^ s^–1^), *v* is the scan rate (V s^–1^), and α_CT_ is the charge-transfer coefficient. In this study, α_CT_ was assumed to be 0.5 owing to the electrochemical symmetry of the investigated redox couples.^51^ Summaries of the estimated anodic, cathodic, and average diffusion coefficients obtained using eqs 2 and 3 are provided in Tables S3 and S4, respectively. The true values of the diffusion coefficients are expected to be within the ranges suggested by eqs 2 and 3.

### Temperature Coefficient Determination

The temperature coefficient (α) of each redox couple of **1**–**3** was estimated using variable-temperature CV measurements in an isothermal electrochemical setup. The temperature of the solution near the working electrode was controlled using a hot plate and quantified using a thermocouple (stainless steel) immersed in the analyte solution. The thermocouple was calibrated using an external temperature controller (Omega Engineering CS8DPT). The solution temperature was increased in ∼3–5 °C intervals in the temperature range ∼23–47 °C. At each temperature, the cell was allowed to equilibrate for ∼3 min before three CV cycles were collected. Solutions were stirred between measurements at different temperatures. The half-wave potentials (*E*_1/2_) for each redox couple of **1**–**3** were extracted from the variable-temperature CV data and plotted against temperature. As the diffusion coefficients for the oxidized and reduced species of individual redox couples are similar (Tables S3 and S4), we make the estimation that the formal potential and the half-wave potential are equal (*E*^0^′ ≈ *E*_1/2_),^51^ thus the slopes of the linear fits to the data of *E*_1/2_ vs temperature plots afford the temperature coefficients of the redox couples with respect to the reference electrode potential. Notably, the electrochemical potential of the Ag/AgNO_3_ reference electrode is also sensitive to temperature.^53^ Accordingly, the true temperature coefficient of a given redox couple is obtained after correcting for the temperature coefficient of the reference electrode potential using the following equations:^23^
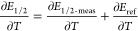
4
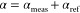
5In these equations, *E*_1/2-meas_ is the measured half-wave potential, *E*_ref_ is the potential of the reference electrode, α is the true temperature coefficient, α_meas_ is the measured temperature coefficient, and α_ref_ is the temperature coefficient of the reference electrode potential.

The temperature coefficient of the non-aqueous Ag/AgNO_3_ reference electrode potential was estimated using non-isothermal open-circuit potential measurements in a two-electrode setup following a modified literature procedure where one Ag/AgNO_3_ reference electrode serves as a working electrode and a second Ag/AgNO_3_ reference electrode serves as a reference/counter electrode.^23^ A three-compartment custom-made glass cell (Adams & Chittenden Scientific) with fine frits separating the two side compartments from the middle compartment was used for the measurements. The cell was arranged such that one of the side compartments was placed in a heating block, while the other side compartment was held at the temperature of the glovebox. For each measurement, ∼10 mL and ∼3 mL of MeCN containing 0.1 M (^*n*^Bu_4_N)(PF_6_) were added to the side and middle compartments, respectively. The temperature of the solution in the side compartment housing the Ag/AgNO_3_ electrode serving as a working electrode was increased in ∼3–4 °C intervals in the temperature range ∼23–43 °C. Thermocouples (stainless steel) were placed in each side compartment at the same height as the electrodes. Temperature measurements and associated calibration were carried out as previously described. At each temperature, the cell was allowed to equilibrate for ∼3 min before *E*_OCP_ was recorded for 120 s while stirring the solution. The average *E*_OCP_ values were plotted against the temperature difference between the two Ag/AgNO_3_ electrodes, and the slope of the linear fit to the data afforded the temperature coefficient of the Ag/AgNO_3_ reference electrode potential (α_ref_). The reported value of 0.53(3) mV °C^–1^ is an average obtained from three independent measurements. This value is in a good agreement with values reported in the literature for the Ag/AgNO_3_ reference electrode potential in MeCN solutions.^30,53^ We note that no correction for the thermal liquid junction potential was made because such contribution should be minimal in a three-compartment double-fritted electrochemical cell using the same electrolyte solution inside and outside of the reference electrode frit.

### Redox Entropy Determination

The temperature coefficient values for the redox couples of **1**–**3** were used to estimate the associated redox reaction entropies (Δ*S*_redox_) using the following equation:^10,14,23^
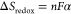
6In this equation, *n* is the number of electrons transferred in the given reaction, *F* is Faraday’s constant, and α is the corrected temperature coefficient.

### Radius of Gyration Determination

The radius of gyration (*R*_g_) for complexes **1**, **1-ox**, **2**, and **3** was calculated using the Multiwfn program (version 3.8(dev))^54,55^ employing the *xyz* coordinates from their respective crystal structures (**1′**, **1-ox′**, **2**, **3′**). The program relies on the following equation for the radius of gyration:
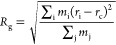
7In this equation, *r* is the coordinate of the nucleus, *r*_c_ is the center of mass, and *m* is the atomic mass.

### UV–Visible–NIR Absorption Spectroscopy

Solution UV–visible–NIR absorption spectra were collected at 22–23 °C on an Agilent Cary 6000i UV–visible–NIR spectrophotometer. Solution UV–visible spectra were collected in the 250–800 nm range for 0.1–0.3 mM samples of complexes **1**, **1-ox**, **2**, and **3**, and ligands HBPMP and (^*n*^Bu_4_N)(FcCOO) in dry MeCN. Solution NIR spectra were collected in the 800–1600 nm range for 2.9–3.0 mM samples of complexes **1**, **1-ox**, and **2** in dry MeCN. The data were collected in a quartz cuvette and treated with a background correction of the MeCN solvent. UV–visible absorption spectra were collected for solid samples of complexes **1-ox**, **2**, and **3** at ∼22 °C in the 250–800 nm range on an Agilent Cary 7000 spectrophotometer equipped with a universal measurement device for direct control over the incident angle and detector position. Samples were prepared by dropcasting concentrated CH_2_Cl_2_ solutions of the complexes onto a quartz disc followed by drying in air. The data were treated with a background correction of the quartz disc and the spectra are reported as normalized absorption spectra where the absorbance for each compound was normalized with the strongest absorbance set to 1.

### Solution Magnetic Measurements

The solution magnetic moments of complexes **1**, **1-ox**, **2**, and **3** were determined using the Evans method^56^ by collecting ^1^H NMR spectra at 22–23 °C (295–296 K) on a Bruker 400 MHz (9.4 T) spectrometer. Each compound (5.5–10.4 mM) was dissolved in a mixture of 2% (*v*/*v*) CH_2_Cl_2_ in CD_3_CN and placed inside an NMR tube containing a sealed capillary with the same solvent mixture but without the to-be-characterized compound as a reference solution. Diamagnetic corrections were carried out based on the empirical formula of each compound (as determined by elemental analysis) using Pascal’s constants.^57^ The paramagnetic molar susceptibility (χ_M_^para^) was calculated using the following equation:^56^
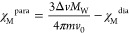
8In this equation, Δν is the frequency difference (Hz) between the CH_2_Cl_2_ resonance in the sample and reference solutions, *M*_w_ is the molecular mass of the to-be-characterized compound (g mol^–1^), *v*_0_ is the operating frequency of the NMR spectrometer (Hz), *m* is the concentration of the to-be-characterized compound (g cm^–3^), and χ_M_^dia^ (cm^3^ mol^–1^) is the diamagnetic contribution to the molar susceptibility (cm^3^ mol^–1^).

### Other Physical Measurements

^1^H and ^19^F{^1^H} NMR spectra were collected at 22–23 °C at 400 and 376 MHz frequencies, respectively, on Bruker 400 MHz (9.4 T) or automated JEOL 400 MHz (9.4 T) spectrometers. Diffusion ordered spectroscopy (DOSY) NMR experiments were collected at 22–23 °C on a JEOL 400 MHz (9.4 T) spectrometer using a linear array of 16 gradient field strengths from 0.03 T m^–1^ to 0.3 T m^–1^, a diffusion time of 0.1 s, and a relaxation delay of 3 s. All chemical shift values (δ) are reported in ppm and coupling constants (*J*) are reported in hertz (Hz). ^1^H NMR spectra are referenced to residual proton signals from the deuterated solvents (7.26 ppm for CDCl_3_, 2.50 ppm for (CD_3_)_2_SO, and 1.94 ppm for CD_3_CN). ^19^F{^1^H} NMR spectra are referenced to an external standard of CFCl_3_ (δ = 0 ppm). The MestReNova 10.0 NMR data processing software was used to analyze and process all recorded NMR spectra. The Bayesian method was used to generate DOSY spectra. The reported value of the diffusion coefficient for **3** in CD_3_CN solution is an average of two independent measurements. Error bars were determined from the width of the DOSY NMR signals. Elemental analyses of all complexes were performed at the CENTC Elemental Analysis Facility at the University of Rochester. Samples for analysis were weighed with a PerkinElmer Model AD 6000 Autobalance and their compositions were determined with a PerkinElmer 2400 Series II Elemental Analyzer. Air-sensitive samples were handled in a VAC Atmospheres glovebox. Electrospray ionization mass spectrometry (ESI–MS) measurements were performed on a Shimadzu LCMS-2020 single quadrupole liquid chromatograph mass spectrometer. All measurements were carried out in MeCN carrier solvent using positive ionization mode. Inductively coupled plasma mass spectrometry (ICP–MS) was performed on a PerkinElmer NexION 2000 ICP–MS instrument in kinetic energy discrimination (KED) mode. Samples were dissolved in a 3% aqueous nitric acid solution and the emissions for Fe and Zn compared to standard solutions.

## Results and Discussion

### Design and Syntheses

**Scheme 1:**
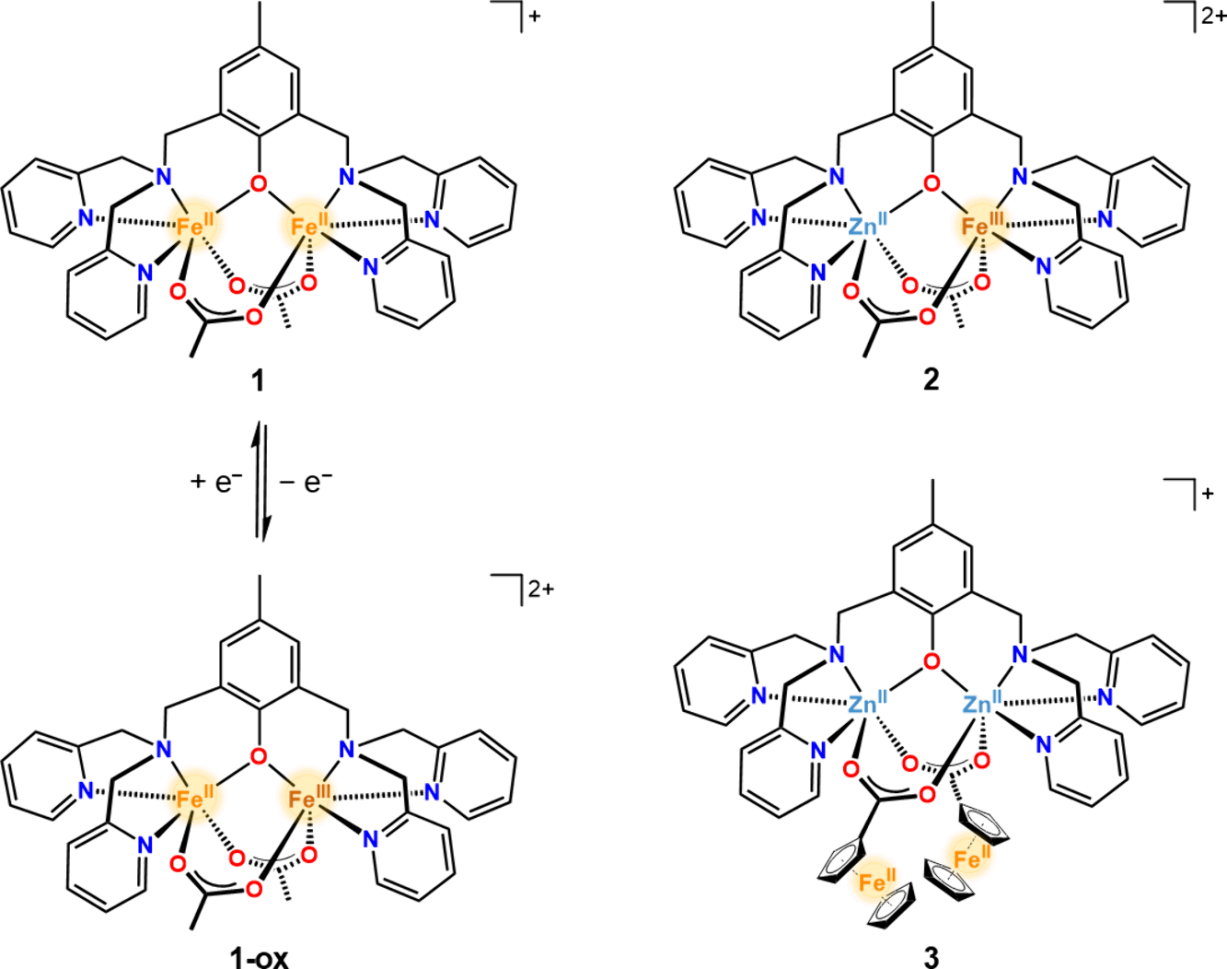
Molecular Structures of the Cationic Complexes Discussed in This Work [(BPMP)Fe_2_(OAc)_2_]^+^, [(BPMP)Fe_2_(OAc)_2_]^2+^, [(BPMP)FeZn(OAc)_2_]^2+^, and [(BPMP)Zn_2_(FcCOO)_2_]^+^, as Observed in **1**, **1-ox**, **2**, and **3**, Respectively The yellow spheres highlight redox-active metal centers.

With the goal of better understanding the effects of the coordination environment of the redox-active center(s) and electronic coupling on the temperature dependence of the electrochemical potential of molecular compounds, we selected dinuclear complexes as model systems owing to their high synthetic modularity and ease of structural characterization. Specifically, we targeted a series of isostructural M_2_ (M = Fe^II^, Fe^III^, Zn^II^) complexes featuring a phenoxo-centered tetrapyridyl ligand and two bridging carboxylates (Scheme 1). In this series, charge, electronic coupling strength, and coordination environment of the redox-active center can be separately modified through the proper selection of metal centers and ancillary carboxylate ligands.

The ligand, HBPMP, was synthesized through an S_N_2 reaction between 2,6-bis(bromomethyl)-4-methylphenol and bis(2-pyridylmethyl)amine (see experimental details and Scheme S1 in the Supporting Information). Reaction of HBPMP with two equivalents of Fe(OAc)_2_ under anaerobic conditions in the presence of NaOAc and (NH_4_)(PF_6_) in MeCN afforded [(BPMP)Fe_2_(OAc)_2_](PF_6_) (**1**) as an orange solid. Similar reaction of HBPMP with one equivalent of Fe(NO_3_)_3_·9H_2_O, three equivalents of Zn(OAc)_2_·2H_2_O, and one equivalent of ZnBr_2_ in the presence of (NH_4_)(PF_6_) in MeOH gave [(BPMP)FeZn(OAc)_2_](PF_6_)_2_ (**2**) as a purple solid. On the other hand, treatment of HBPMP with NaOMe in MeOH, followed by addition of two equivalents each of Zn(OTf)_2_ and (^*n*^Bu_4_N)(FcCOO), and excess of NaPF_6_ afforded [(BPMP)Zn_2_(FcCOO)_2_](PF_6_)_0.97_(OTf)_0.03_·0.2NaPF_6_ (**3**) as a yellow solid (Figures S5 and S6). Note that all three complexes feature two ancillary carboxylate ligands. The carboxylate ligands are substituted with a redox-inactive methyl group in **1** and **2**, and a redox-active ferrocenyl group in **3**.

### Crystal Structures

Slow diffusion of Et_2_O vapor into concentrated CH_2_Cl_2_ and MeCN solutions of **1** and **2** gave orange and purple plate-shape crystals of **1**·solvent (**1′**) and **2**, respectively. Slow evaporation of a concentrated MeCN solution of **3** afforded yellow plate-shape crystals of [(BPMP)Zn_2_(FcCOO)_2_](PF_6_)_0.78_(OTf)_0.22_·2MeCN (**3′**). Single-crystal X-ray diffraction analysis at 100 K revealed that **1′**, **2**, and **3′** are isostructural and crystallize in the triclinic space group *P*1̅ with one cationic M_2_ (M = Fe^II^, Fe^III^, Zn^II^) complex in the asymmetric unit (Figures S1, S4, and S7). The general structure of the cationic complexes in **1′**, **2**, and **3′** consists of two nearly identical metal centers in distorted octahedral coordination environments, each comprised of a μ-phenoxo O atom, a tertiary amine N atom, and two pyridyl N atoms from (BPMP)^−^ and two O atoms from the ancillary carboxylate ligands (Figure 1). In the structure of **2**, there is one Fe atom and one Zn atom between the two metal sites, and each site contains a mixture of the two metals. Inductively coupled plasma mass spectrometry (ICP–MS) further confirms the 1:1 molar ratio of Fe and Zn in **2**.

The mean M–O_phenoxo_, M–O_carboxylate_, and M–N distances of 2.035(2)–2.0573(9) Å, 2.022(1)–2.0939(7) Å, and 2.155(1)–2.2185(6) Å across the series (Table 1) are consistent with reported distances for related phenoxo-bridged Fe^II^_2_, Fe^III^Zn^II^, and Zn^II^_2_ complexes with [MN_3_O_3_] coordination environments.^31−37,39,58^ These bond lengths are indicative of high-spin Fe^II^ and Fe^III^ centers in **1′** and **2**, respectively. The mean M–O_phenoxo_–M bond angle of 115.80(9)° and intramolecular M···M distance of 3.4480(5) Å in the structure of **2** are slightly larger than those observed in the homometallic analogues **1′** and **3′**, consistent with literature reports.^31−37,39,58^ In contrast, the O–C–O bond angle for the carboxylate ligands is similar across the series, highlighting the insignificant structural changes associated with altering the ancillary carboxylate ligand from (OAc)^−^ to (FcCOO)^−^. Finally, all three complexes display a significant structural distortion from ideal octahedral geometry, as indicated with mean *trans*-O–M–N bond angles of 165.43(3)–168.95(4)° across the series and an octahedral distortion parameter (*∑*_sum_) of 80.74(2)°, 68.75(2)°, and 74.66(2)° for **1′**, **2**, and **3′**, respectively. Taken together, the comparable structural metrics for **1′**, **2**, and **3′** corroborate the use of this series as isostructural analogues for probing the influence of charge, electronic coupling, and coordination environment on the temperature dependence of the electrochemical potential of Fe-based redox couples. In further support of that, we calculated the radius of gyration for the cationic complexes in **1′**, **2**, and **3′** using their crystallographic coordinates. Values of 3.96 Å, 4.01 Å, and 4.53 Å were obtained for the cationic complexes in **1′**, **2**, and **3′**, respectively, indicating that replacing bridging (OAc)^−^ ligands with (FcCOO)^−^ results in ∼13–14% increase in the radius of gyration. While non-negligible, this increase in the radius of gyration of [(BPMP)Zn_2_(FcCOO)_2_]^+^ as compared to [(BPMP)Fe_2_(OAc)_2_]^+^ and [(BPMP)FeZn(OAc)_2_]^2+^ is relatively small.

### UV–Visible–NIR Spectroscopy

To probe the electronic structures of compounds **1**–**3** in solution, UV–visible absorption spectra were collected for samples in MeCN. The spectrum of **1** (Figure S9) shows three key features: an intense absorption band at 256 nm (ε = 14680 M^–1^ cm^–1^) and two lower-intensity bands at 306 nm (ε = 3840 M^–1^ cm^–1^) and 441 nm (ε = 2350 M^–1^ cm^–1^). We assign the two highest-energy bands to ligand-based transitions owing to the similar bands observed for complexes **2** and **3**, and free ligand HBPMP (Figures S9–S13). The absorption at 441 nm is assigned to a metal–ligand charge-transfer (MLCT) transition from Fe^II^ to pyridine, in accord with literature precedent for similar Fe_2_ complexes.^32−34,58^ In addition to ligand-based transitions, the spectrum of **2** (Figure S10) shows a broad absorption centered at 560 nm (ε = 950 M^–1^ cm^–1^), which is ascribed to a ligand–metal charge-transfer (LMCT) transition from the phenolate to Fe^III^.^32,33,37^ Similarly, compound **3** displays a broad, weak absorption centered at 444 nm (ε = 350 M^–1^ cm^–1^) (Figure S11). On the basis of the presence of *d*^10^ Zn^II^ centers in **3** and close similarity with the spectrum of (^*n*^Bu_4_N)(FcCOO) (Figure S14), we assign this band to *d*–*d* transitions within the ferrocenyl groups on the ancillary carboxylate ligands.^59,60^

The different spectral features observed for **1** and **2**, originating from the presence of Fe^II^ vs Fe^III^ centers in these compounds, prompted us to investigate the stability of **1** toward oxygen in air using absorption spectroscopy. The spectrum of **1** changes gradually over the course of 60 min in air (Figure S15). Specifically, the intensity of the MLCT band at 441 nm decreases, while the absorption increases below ∼420 nm and above ∼500 nm. The observation of two isosbestic points at 419 and 494 nm indicates the clean conversion to a new species. Comparison with the spectrum of **2** indicates that the new species is the mixed-valence Fe^II^Fe^III^ analogue of **1**. Indeed, reaction of an MeCN solution of **1** with oxygen in air in the presence of (NH_4_)(PF_6_) afforded [(BPMP)Fe_2_(OAc)_2_](PF_6_)_2_·0.2(NH_4_)(PF_6_) (**1-ox**) as a black solid. Slow diffusion of DME vapor into a concentration CH_2_Cl_2_ solution of **1-ox** afforded dark green plate-shaped single crystals of **1-ox**·DME (**1-ox′**). Crystallographic analysis revealed **1′** and **1-ox′** to be isostructural, however, the distinct mean Fe–O bond distances for the two Fe centers in **1-ox′** of 1.972(2) Å and 2.092 Å are indicative of valence-localized high-spin Fe^III^ and Fe^II^ sites (Figures S2 and S3, Tables S1 and S2).^32,33^ The UV–visible absorption spectrum of **1-ox** in MeCN is shown in Figure S16 and is very similar to the spectrum collected of **1** after 60 min exposure to ambient air (Figure S17). Furthermore, the NIR absorption spectrum of **1-ox** (Figure S18) shows a characteristic Fe-based intervalence charge-transfer (IVCT) band at ∼1300 nm (ε = 270 M^–1^ cm^–1^),^32−34,61^ whereas the other Fe-containing complexes exhibit minimal absorption in the 800–1600 nm range (Figure S19). While the incompleteness of the band within the accessible solvent window precludes detailed analysis of the IVCT behavior in **1-ox**, similar Fe^II^Fe^III^ complexes have been assigned to class II mixed-valence complexes with weak to moderate electronic coupling between Fe centers.^33^ Notably, **1-ox** and **2** form a pair of isostructural and isocharge compounds with and without electronic communication between metal centers, respectively, attractive for electrochemical analysis.

Finally, the close similarities between the absorption spectra collected for MeCN solutions of **1**, **1-ox**, **2**, and **3**, and solid samples of these compounds (Figures S20–S23) suggest that the structures of the dinuclear complexes determined from X-ray diffraction are preserved in MeCN solutions.

### Solution Magnetic Properties

To further probe the solution electronic structures of compounds **1**, **1-ox**, **2**, and **3**, and assess their magnetic properties, dc magnetic susceptibility data were collected at room temperature (22–23 °C) using the Evans method.^56^ Average values of χ_M_*T* = 6.95(4) cm^3^ K mol^–1^, 8.50(4) cm^3^ K mol^–1^, 4.38(1) cm^3^ K mol^–1^, and ∼0 cm^3^ K mol^–1^ were obtained for **1**, **1-ox**, **2**, and **3**, respectively. Assuming two magnetically non-interacting Fe^II^ centers in **1**, the value of χ_M_*T* per Fe^II^ ion of 3.48(4) cm^3^ K mol^–1^ corresponds to *g* = 2.15(1), consistent with octahedral, high-spin Fe^II^ (*S* = 2) centers.^32,62^ The increase in χ_M_*T* upon oxidation of **1** to **1-ox** is consistent with the increase in spin state from *S* = 2 to *S* = ^5^/_2_ for one of the Fe centers. The value of χ_M_*T* for **2** is in accord with an octahedral, high-spin Fe^III^ (*S* = ^5^/_2_) center next to a diamagnetic Zn^II^ center,^37^ affording *g* = 2.00(1). Similarly, the near-zero magnetic moment obtained for **3** is as expected for a complex containing two diamagnetic Zn^II^ centers and two ferrocenyl groups. In sum, magnetic measurements of solutions of **1**, **1-ox**, **2**, and **3**, underscore the presence of octahedral, high-spin Fe centers in **1**, **1-ox**, and **2**, together with the diamagnetic nature of **3**.

### Room-Temperature Electrochemistry

**Figure 2:**
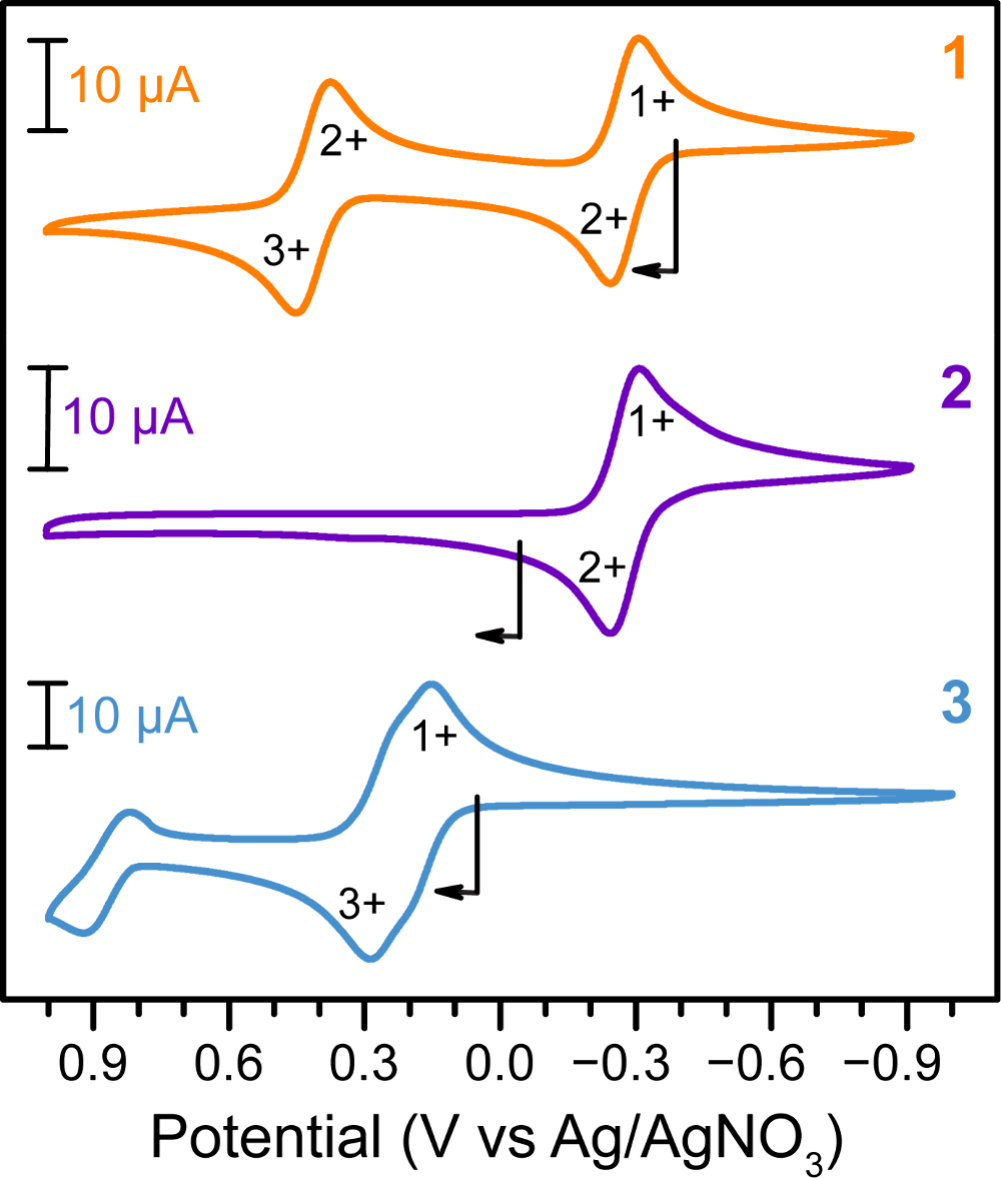
Cyclic voltammograms of 0.9 mM of **1**, 0.9 mM of **2**, and 0.8 mM of **3** in MeCN containing 0.1 M (^*n*^Bu_4_N)(PF_6_) supporting electrolyte collected at room temperature (23–25 °C) using 100 mV s^–1^ scan rate. Vertical black lines and arrows denote the open-circuit potentials and scan direction, respectively. Black numbers denote the charge states of complexes at respective potentials. Glassy carbon, Ag/AgNO_3_, and Pt mesh were used as working, reference, and counter electrodes, respectively.

Having established the geometric structures of **1**, **1-ox**, **2**, and **3** in both solid state and solution, and demonstrated the minimal structural perturbation across the series together with determination of the metal oxidation states and spin states, we sought to investigate their electrochemical properties using cyclic voltammetry (CV). The cyclic voltammograms of **1**, **2**, and **3** in MeCN containing 0.1 M (^*n*^Bu_4_N)(PF_6_) supporting electrolyte collected at room temperature (23–25 °C) are depicted in Figure 2 (see also Figure S8). The voltammogram of **1** exhibits two quasi-reversible processes with half-wave potentials of *E*_1/2_ = −0.335 and 0.389 V vs Ag/AgNO_3_, corresponding to Fe^II^Fe^III^/Fe^II^_2_ and Fe^III^_2_/Fe^II^Fe^III^ redox couples, respectively (Table 2). The potential separation of Δ*E*_1/2_ = 0.724 V corresponds to a comproportionation constant of *K*_C_ = 1.92 × 10^12^ for the reaction [(BPMP)Fe_2_(OAc)_2_]^3+^ + [(BPMP)Fe_2_(OAc)_2_]^+^ → 2[(BPMP)Fe_2_(OAc)_2_]^2+^, indicating that the mixed-valence complex is stable toward disproportionation. The CV of **1-ox** is identical to the one obtained of **1** albeit with a different open-circuit potential (Figure S24), consistent with starting from a Fe^II^Fe^III^ vs Fe^II^_2_ complex.

**Table 2:** Selected Electrochemical and Thermodynamic Parameters for **1**, **2**, and **3** in MeCN Solutions^a^

Compound	Charge State	Redox Couple	*E*_1/2_^b^ (V vs Ag/AgNO_3_)	*α*^c^ (mV °C^–1^)	Δ*S*_redox_^d^ (J K^–1^ mol^–1^)
**1**	2+/1+	Fe^II^Fe^III^/Fe^II^_2_	–0.335	1.07(4)	103(5)
	3+/2+	Fe^III^_2_/Fe^II^Fe^III^	0.389	1.7(1)	164(10)
**2**	2+/1+	Fe^III^/Fe^II^	–0.345	1.07(2)	103(3)
**3**^e^	3+/1+	2×Fe^III^/Fe^II^	0.224	0.53(4)	51(4)

a Data were collected for MeCN solutions containing 1.1–5.0 mM of metal complex (**1**, **2**, **3**) and 0.1 M (^*n*^Bu_4_N)(PF_6_) supporting electrolyte using glassy carbon working electrode, Ag/AgNO_3_ reference electrode, and Pt counter electrode.

b Half-wave potentials at 23.9 °C, 23.3 °C, and 24.4 °C for **1**, **2**, and **3**, respectively.

c Temperature coefficient (α) obtained from variable-temperature CV measurements and calculated using eqs 4 and 5; average values from 3–5 independent measurements and error bars denote standard deviations of those measurements.

d Change in entropy associated with a given redox reaction, calculated using eq 6; errors were estimated using error propagation of the average temperature coefficient values.

e Two overlapping redox features assigned to the two ferrocenyl groups on the bridging carboxylate ligands; the half-wave potential was estimated using the cathodic peak of the first redox event and the anodic peak of the second event.

The CV of **2** shows a single quasi-reversible process with *E*_1/2_ = −0.345 V vs Ag/AgNO_3_, agreeing well with the Fe^II^Fe^III^/Fe^II^_2_ redox potential for **1**. In contrast, the CV of **3** collected in the same potential window of 1.0 V to −1.0 V vs Ag/AgNO_3_ exhibits a broad quasi-reversible wave centered at 0.224 V vs Ag/AgNO_3_ and a less intense quasi-reversible wave with *E*_1/2_ = 0.871 V vs Ag/AgNO_3_. We assign the wave at less positive potentials to two overlapping redox processes of the ferrocenyl groups on the ancillary bridging ligands. As the two ferrocenyl groups are not in direct electronic communication, their redox potentials are very similar and thus overlapping redox waves are observed. Accordingly, this broad wave can be considered as a pseudo-two-electron redox wave with the overall charge of the Zn^II^_2_ complex in **3** changing by two units (+3/+1) (Figure 2, black numbers). The more anodically shifted redox wave in the CV of **3** is assigned to the oxidation (and corresponding reduction) of a bridging carboxylate moiety.^63^

To gain further insights into the redox behavior of the dinuclear complexes, CVs were collected for each compound at variable scan rates (25–400 mV s^–1^) (Figures S25–S30). Plots of the anodic and cathodic peak currents vs the square root of scan rate exhibit a linear correlation for all redox couples, indicative of diffusion-limited electrochemical processes (Figures S31–S36). Using Randles–Ševčík analysis,^49−52^ we estimated the diffusion coefficients for the oxidized and reduced species of each redox couple from the linear fits of the data (Tables S3 and S4). The similar values of the diffusion coefficients for the oxidized and reduced species for each redox couple of **1**, **1-ox**, **2**, and **3** underscores that the difference between *E*_1/2_ and the formal electrochemical potential (*E*^0^′) is minimal. As such, *E*_1/2_ may be employed as a proxy for *E*^0^′ in variable-temperature electrochemical measurements (vide infra). Furthermore, the similar diffusion coefficients obtained for the redox processes across the series of dinuclear complexes illustrates the similar size and mobility of the four cationic complexes in MeCN solutions. We further note that the values of the diffusion coefficients for **3** estimated from Randles–Ševčík analysis are consistent with those calculated using the radius of gyration and Stokes–Einstein equation,^51^ as well as those measured using diffusion ordered spectroscopy NMR experiments (Table S5).^64^

### Variable-Temperature Electrochemistry

**Figure 3:**
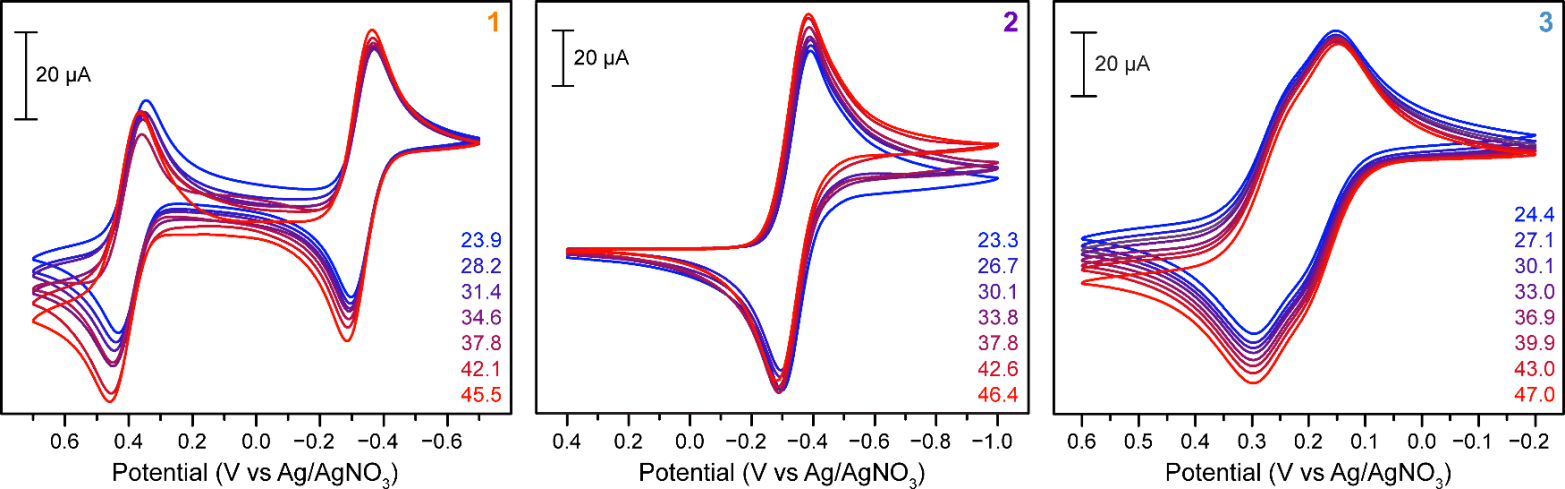
Cyclic voltammograms of 1.9 mM of **1**, 4.7 mM of **2**, and 1.9 mM of **3** in MeCN containing 0.1 M (^*n*^Bu_4_N)(PF_6_) supporting electrolyte collected at variable temperatures (23–47 °C) using 100 mV s^–1^ scan rate. Glassy carbon, Ag/AgNO_3_, and Pt mesh were used as working, reference, and counter electrodes, respectively.

**Figure 4:**
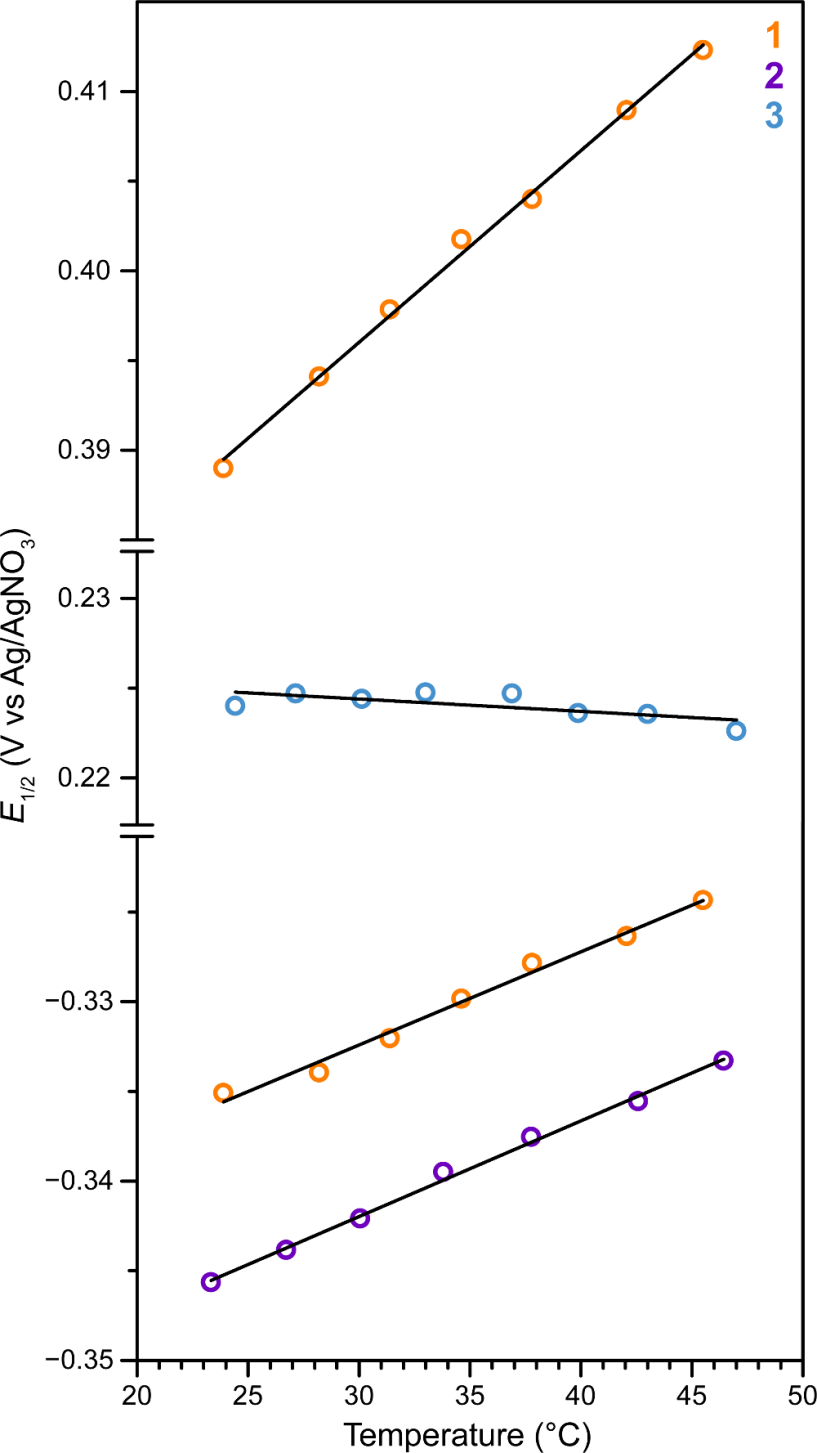
Temperature dependence of the redox potential (*E*_1/2_) for **1**, **2**, and **3** in MeCN containing 0.1 M (^*n*^Bu_4_N)(PF_6_) supporting electrolyte, as obtained from the variable-temperature cyclic voltammetry data shown in Figure 3. Colored circles and black lines correspond to experimental data and linear fits, respectively.

In order to probe the impact of charge, electronic coupling, and coordination environment on the temperature dependence of the redox potentials for the isostructural series **1**–**3**, variable-temperature CV measurements were undertaken for MeCN solutions using isothermal setup, in which the working electrode, reference electrode, and counter electrode are heated in a single-compartment cell. The CVs collected in the temperature range 23–47 °C for **1**, **2**, and **3** are displayed in Figure 3. The voltammograms of **1** and **2** shift to more positive (or less negative) potentials with increasing temperature, consistent with cationic redox couples.^8,10,12−14,18,19,21,23,27^ In contrast, the voltammogram of **3** shows minimal temperature dependence. The temperature dependence of *E*_1/2_ is better visualized in Figure 4, where the slopes of the linear fits to the data represent the temperature coefficients (α) for respective redox couples referenced to the Ag/AgNO_3_ reference electrode. As the redox potential of the reference electrode is also sensitive to temperature, the measured temperature coefficients must be corrected by adding the temperature coefficient of the Ag/AgNO_3_ reference electrode using eqs 4 and 5. Variable-temperature open-circuit potential measurements between two Ag/AgNO_3_ reference electrodes in MeCN containing 0.1 M (^*n*^Bu_4_N)(PF_6_) using a non-isothermal setup afforded a temperature coefficient of 0.53(3) mV °C^–1^ (Figures S37 and S38), in agreement with values reported in similar electrolyte solutions.^30,53^ Accordingly, the two redox couples of **1** exhibit temperature coefficients of α = 1.07(4) mV °C^–1^ (2+/1+) and α = 1.7(1) mV °C^–1^ (3+/2+) (Table 2).

Similarly, the Fe^III^/Fe^II^ redox couple in **2** affords a value of α = 1.07(2) mV °C^–1^. The identical temperature coefficients obtained for the Fe^III^/Fe^II^ redox wave corresponding to 2+/1+ change in overall charge of the cationic complexes in **1** and **2** indicates that electronic coupling strength does not significantly impact the temperature dependence of the electrochemical potential in these dinuclear Fe-based complexes. Notably, temperature coefficients of the 2+/1+ redox wave of **1** and **2** are significantly larger than reported values for MeCN solutions of tris(2,2′-bipyridine) complexes of chromium and ruthenium undergoing the same change in overall charge.^14^ In contrast, the pseudo-two-electron redox wave of **3** exhibits negligible temperature dependence as compared to the Ag/AgNO_3_ reference electrode (Figure 4), affording a temperature coefficient of α = 0.53(4) mV °C^–1^, which is nonetheless about 5× larger than the value of the Fc^+^/Fc couple measured in ionic liquids and water solutions.^14,65^

Using eq 1, the redox reaction entropy associated with each redox wave of compounds **1**–**3** can be estimated. These values of Δ*S*_redox_ are listed in Table 2. Previous reports have found a linear relationship between Δ*S*_redox_ and the change in charge density of mononuclear transition metal complexes.^14^ Specifically, the values of Δ*S*_redox_ are in correspondence with the dielectric continuum function (*Z*_ox_^2^/*r*_ox_ – *Z*_red_^2^/*r*_red_) from the Born model of the redox reaction entropy (eq 9).^12−14^
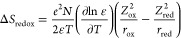
9In this equation, *Z*_ox_ and *Z*_red_ represent the charge of the oxidized and reduced species of a redox couple, respectively, *r*_ox_ and *r*_red_ denote the ionic radius of the oxidized and reduced species, respectively, *e* is the elementary charge, *T* is the temperature, *N* is Avogadro’s number, and ε is the static dielectric constant of the solvent. Note that the radii of the cationic complexes in **1**–**3** do not change significantly with charge, as evidenced by the near identical radius of gyration for **1** and **1-ox** (3.96 Å vs 3.99 Å). The ∼1.6-fold larger redox entropy for the 3+/2+ redox wave of **1** as compared to the 2+/1+ wave agrees well with eq 9 and underscores the importance of charge density change in the design of compounds with highly temperature-sensitive redox potentials. Nevertheless, the observation of a much smaller redox entropy for the pseudo-two-electron redox wave (3+/1+) of **3** as compared to the 2+/1+ wave of **1** and **2** indicates that charge density change alone does not dictate the temperature coefficient of the redox potential. Rather the coordination environment of the redox-active center(s) plays a critical role as well. Our study highlights that the electrochemical properties of Fe centers in a pseudo-octahedral [FeN_3_O_3_] coordination environment comprising a phenoxo-centered tetrapyridyl ligand and two ancillary carboxylate ligands are more sensitive to temperature changes than those of the Fe center in ferrocenecarboxylate.

## Conclusions

The foregoing results demonstrate that the coordination environment of a redox-active metal center significantly impacts the temperature dependence of its redox potential while the strength of electronic coupling between metal centers plays a smaller role. Specifically, for a series of isostructural homo- and heterometallic M_2_ (M = Fe^II^, Fe^III^, Zn^II^) complexes **1**–**3**, the temperature coefficient of the Fe^III^/Fe^II^ redox couple is 2-fold greater for Fe centers in a pseudo-octahedral [FeN_3_O_3_] coordination environment as compared to that in ferrocenecarboxylate, despite larger change in the charge of the cationic complex for the latter (2+/1+ vs 3+/1+). In contrast, the temperature coefficient of the Fe^III^/Fe^II^ redox couple corresponding to the same change in charge (2+/1+) is identical for the Fe_2_ and FeZn analogues, in spite of the presence of electronic coupling between Fe sites in the former. This investigation suggests that coordination environment and charge density change, as opposed to electronic coupling strength, are key parameters to consider toward optimizing the temperature dependence of the redox potential and the associated redox entropy of molecular compounds. Work is underway to investigate the impact of coordination environment, charge density, and electronic coupling on redox entropy in other molecular systems with the goal of generating generalizable design principles for compounds displaying electrochemical potentials with target temperature sensitivity, in particular those that exhibit highly temperature-sensitive redox potentials suitable for applications in waste-heat harvesting and electrochemical sensing.

## Supporting Information Available

Additional experimental details and characterization data, including NMR spectra, X-ray crystallographic data, UV–visible–NIR spectra, and electrochemical data (PDF)
