# Supplementary material for: Effect of Coordination Environment and Electronic Coupling on Redox Entropy in a Family of Dinuclear Complexes
Source: ACS Electrochem. 2025 Feb 18;1(5):741–53. doi: 10.1021/acselectrochem.4c00186 (PMC12051193; doi:10.1021/acselectrochem.4c00186)
Supplement: Supplementary file 1 — ec4c00186_si_001.pdf [file ec4c00186_si_001.pdf]

Supporting Information for:

Effect of Coordination Environment and Electronic Coupling on Redox Entropy in a Family of Dinuclear Complexes

Daniela Carmona-Pérez, Meiqin Gao, Samantha Andes, William W. Brennessel, and *Agnes E. Thorarinsdottir**

Department of Chemistry, University of Rochester, Rochester, New York 14627, USA

*Correspondence to: agnes.thorarinsdottir@rochester.edu (A.E.T.)

Table of Contents

A. Supplementary Text

Synthesis of 2,6-bis(hydroxymethyl)-4-methylphenol……………………………………..S3
Synthesis of 2,6-bis(bromomethyl)-4-methylphenol……………………………………….S3
Synthesis of HBPMP ligand……………………………………………………………….S3
Synthesis of (^{n}Bu$_4$N)(FcCOO)…………………………………………………………..S4

B. Supplementary Scheme

Scheme S1. Overview of HBPMP ligand synthesis………………………………………..S5

C. Supplementary Figures

Figure S1. Crystal structure of **1′**……………………………………………………………….S6
Figure S2. Crystal structure of cationic complex in **1-ox′**……………………………………….S7
Figure S3. Crystal structure of **1-ox′**…………………………………………………………...S8
Figure S4. Crystal structure of **2**………………………………………………………………S9
Figure S5. ^{1}H NMR spectrum of **3** in DMSO-d_6…………………………………………………S10
Figure S6. ^{19}F{^{1}H} NMR spectrum of **3** in DMSO-d_6…………………………………………S11
Figure S7. Crystal structure of **3′**…………………………………………………………...S12
Figure S8. Comparison of CVs of **1–3** with and without iR_u compensation…….…………S13
Figure S9. UV–visible absorption spectrum of **1** in MeCN……………………………………S14
Figure S10. UV–visible absorption spectrum of **2** in MeCN…………………………………S15
Figure S11. UV–visible absorption spectrum of **3** in MeCN…………………………………..S16
Figure S12. Comparison of UV–visible absorption spectra of **1**, **2**, and **3** in MeCN…………S17
Figure S13. UV–visible absorption spectrum of HBPMP in MeCN…………………………S18
Figure S14. UV–visible absorption spectrum of (^{n}Bu$_4$N)(FcCOO) in MeCN………………..S19

Figure S15. UV–visible absorption spectra of **1** in MeCN upon exposure to air……………S20
Figure S16. UV–visible absorption spectrum of **1-ox** in MeCN………………………………S21
Figure S17. Comparison of UV–visible absorption spectra of Fe_2 complexes in MeCN….S22
Figure S18. NIR absorption spectrum of **1-ox** in MeCN………………………………………S23
Figure S19. Comparison of NIR absorption spectra of **1**, **1-ox**, and **2** in MeCN……………S24
Figure S20. UV–visible absorption spectrum of solid **1-ox**……………………………………S25
Figure S21. UV–visible absorption spectrum of solid **2**………………………………………S26
Figure S22. UV–visible absorption spectrum of solid **3**………………………………………S27
Figure S23. Comparison of UV–visible absorption spectra of solids **1-ox**, **2**, and **3**……...S28
Figure S24. Comparison of CVs of **1** and **1-ox** in MeCN at ambient temperature…………S29
Figure S25. Variable-scan-rate CV of **1** (2+/1+) in MeCN at ambient temperature…………S30
Figure S26. Variable-scan-rate CV of **1** (3+/2+) in MeCN at ambient temperature…………S31
Figure S27. Variable-scan-rate CV of **1-ox** (2+/1+) in MeCN at ambient temperature……S32
Figure S28. Variable-scan-rate CV of **1-ox** (3+/2+) in MeCN at ambient temperature……S33
Figure S29. Variable-scan-rate CV of **2** in MeCN at ambient temperature…………………S34
Figure S30. Variable-scan-rate CV of **3** in MeCN at ambient temperature…………………S35
Figure S31. Randles–Ševčík plot for **1** (2+/1+) in MeCN at ambient temperature…………S36
Figure S32. Randles–Ševčík plot for **1** (3+/2+) in MeCN at ambient temperature…………S37
Figure S33. Randles–Ševčík plot for **1-ox** (2+/1+) in MeCN at ambient temperature………S38
Figure S34. Randles–Ševčík plot for **1-ox** (3+/2+) in MeCN at ambient temperature………S39
Figure S35. Randles–Ševčík plot for **2** in MeCN at ambient temperature…………………..S40
Figure S36. Randles–Ševčík plot for **3** in MeCN at ambient temperature…………………..S41
Figure S37. Plot of E_{OCP} vs time for Ag/$AgNO_3$ electrode at different temperatures……….S42
Figure S38. Plot of E_{OCP} vs ΔT for Ag/$AgNO_3$ electrode in MeCN…………………………...S43

D. Supplementary Tables

Table S1. Crystallographic data for **1′**, **1-ox′**, **2**, and **3′**………………………………………S44
Table S2. Selected mean interatomic distances and angles for **1-ox′**……………………….S45
Table S3. Diffusion coefficients for **1**, **1-ox**, **2**, and **3** in MeCN (reversible couple)……….S46
Table S4. Diffusion coefficients for **1**, **1-ox**, **2**, and **3** in MeCN (irreversible couple)……….S47
Table S5. Comparison of diffusion coefficients for **3**……………………………………………S48

E. References……………………………………………………………………………………..…S49

A. Supplementary Text

Synthesis of 2,6-Bis(hydroxymethyl)-4-methylphenol. This compound was synthesized following a modified literature procedure.[1] 4-Methylphenol (6.09 g, 56.3 mmol) was dissolved in a solution of sodium hydroxide (3.05 g, 76.3 mmol) in deionized H_2O (11.5 mL). To a stirring solution of this, a 37% (w/w) formaldehyde solution in H_2O (13 mL) was added dropwise and the resulting light yellow solution was stirred for 20 h at 25 °C to afford a white suspension. The white solid was collected by vacuum filtration and dissolved in deionized H_2O (50 mL). The pH of this solution was adjusted to ~5 by addition of glacial acetic acid. Stirring for 15 minutes at 25 °C resulted in the formation of a white precipitate. This precipitate was collected by vacuum filtration and dried with suction on the filter for 2 h to afford the title compound as a white solid (3.30 g, 35%). ^{1}H NMR (400 MHz, $(CD_3)_2SO$, 22 °C): δ 8.30 (s, 1H), 6.94 (s, 2H), 5.18 (s, 2H), 4.51 (s, 4H), 2.20 (s, 3H).

Synthesis of 2,6-Bis(bromomethyl)-4-methylphenol. This compound was synthesized following a modified literature procedure.[1] 2,6-Bis(hydroxymethyl)-4-methylphenol (3.95 g, 23.5 mmol) was dissolved in 33% (w/w) hydrobromic acid solution in acetic acid (32 mL) to give a white yellow suspension. After stirring vigorously at 25 °C for 17 h, the mixture was diluted with deionized H_2O (30 mL) and stirred for additional 30 minutes. The resulting off-white precipitate was collected by vacuum filtration and washed with cold deionized H_2O (50 mL). This solid was then dissolved in deionized H_2O (30 mL) and extracted with diethyl ether (3 × 30 mL). The combined organic layer was washed with cold deionized H_2O (2 × 30 mL) and dried over Na_2SO_4. Hexanes (180 mL) was added to the filtrate to induce precipitation of solid impurities. The precipitate was removed by filtration and the colorless filtrate was evaporated to dryness under reduced pressure to give the product as a white solid (1.34 g, 19%). ^{1}H NMR (400 MHz, $CDCl_3$, 22 °C): δ 7.08 (s, 2H), 5.49 (s, 1H), 4.54 (s, 4H), 2.26 (s, 3H).

Synthesis of 2,6-Bis{[bis(2-pyridylmethyl)amino]methyl}-4-methylphenol (HBPMP). This compound was synthesized following a modified literature procedure.[2] Under an atmosphere of dinitrogen, a mixture of bis(2-pyridylmethyl)amine (3.89 g, 19.5 mmol) and triethylamine (3.78 g, 37.4 mmol) in THF (6 mL) was added dropwise to a solution of 2,6-bis(bromomethyl)-4-methylphenol (2.77 g, 9.42 mmol) in THF (20 mL) at 0 °C. After the addition was complete, formation of triethylammonium bromide salt was observed. The resulting mixture was warmed to 25 °C and left stirring at that temperature for 18 h. Then, the mixture was heated to reflux and

stirred for additional 5 h. Afterwards, the triethylammonium salt was removed by filtration and the resulting yellow solution was concentrated under reduced pressure to give a yellow oily residue. This residue was suspended in deionized H_2O (30 mL) and extracted with CH_2Cl_2 (3 × 30 mL). The combined organic layer was washed with brine (30 mL), dried over Na_2SO_4, and filtered, and the solvent was removed under reduced pressure to give a yellow oily residue. This residue was purified by flash column chromatography on neutral alumina using a 5% MeOH/95% CH_2Cl_2 solvent mixture as the eluent. The product was obtained as a brown oil (1.05 g, 21%). ^{1}H NMR (400 MHz, $CDCl_3$, 22 °C): δ 8.48 (d, 4H), 7.56 (dt, 4H), 7.47 (d, 4H), 7.08 (t, 4H), 6.97 (s, 2H), 3.86 (s, 8H), 3.77 (s, 4H), 2.20 (s, 3H). UV–visible absorption spectrum (0.2 mM, MeCN, 22–23 °C): 262 nm (ε = 12150 M^{-1} cm^{-1}), 284 nm (shoulder, ε = 4080 M^{-1} cm^{-1}).

Synthesis of Tetrabutylammonium Ferrocenecarboxylate (^{n}Bu$_4$N)(FcCOO). This compound was synthesized following a modified literature procedure.[3] Ferrocenecarboxylic acid (1.00, 4.35 mmol) was dissolved in MeOH (60 mL) to give a dark orange solution. To this, a methanolic solution of tetrabutylammonium hydroxide (6.53 mL, 1.0 M) was added dropwise, giving a bright orange solution that was stirred at 25 °C for 1 h. Afterwards, the solvent was removed under reduced pressure, giving a dark orange residue that was dissolved in MeCN (8 mL), and diethyl ether (120 mL) was added to precipitate the product as a light orange solid that was collected by filtration (0.906 g, 44%). ^{1}H NMR (400 MHz, $CDCl_3$, 22 °C): δ 4.71 (t, 2H), 4.19 (s, 5H), 4.11 (t, 2H), 3.29 (m, 8H), 1.61 (m, 8H), 1.41 (m, 8H), 0.97 (t, 12H). UV–visible absorption spectrum (0.3 mM, MeCN, 22–23 °C): 329 nm (ε = 100 M^{-1} cm^{-1}), 444 nm (ε = 110 M^{-1} cm^{-1}).

B. Supplementary Scheme

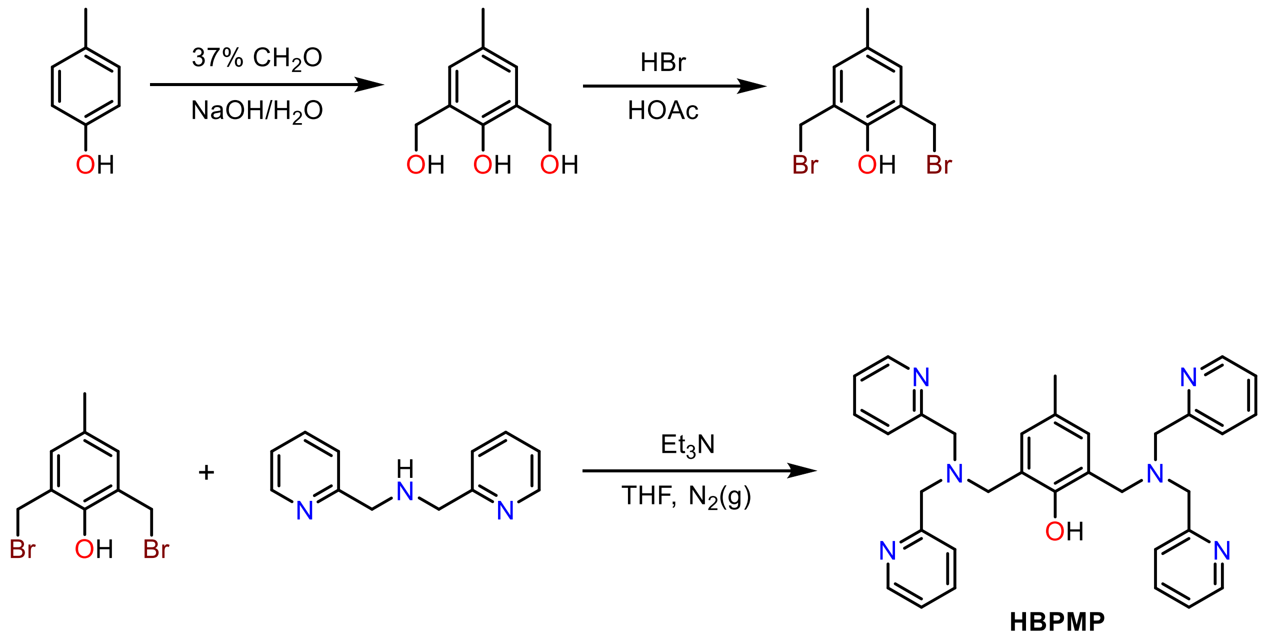

Scheme S1. Syntheses of organic ligand precursors and ligand HBPMP.

C. Supplementary Figures

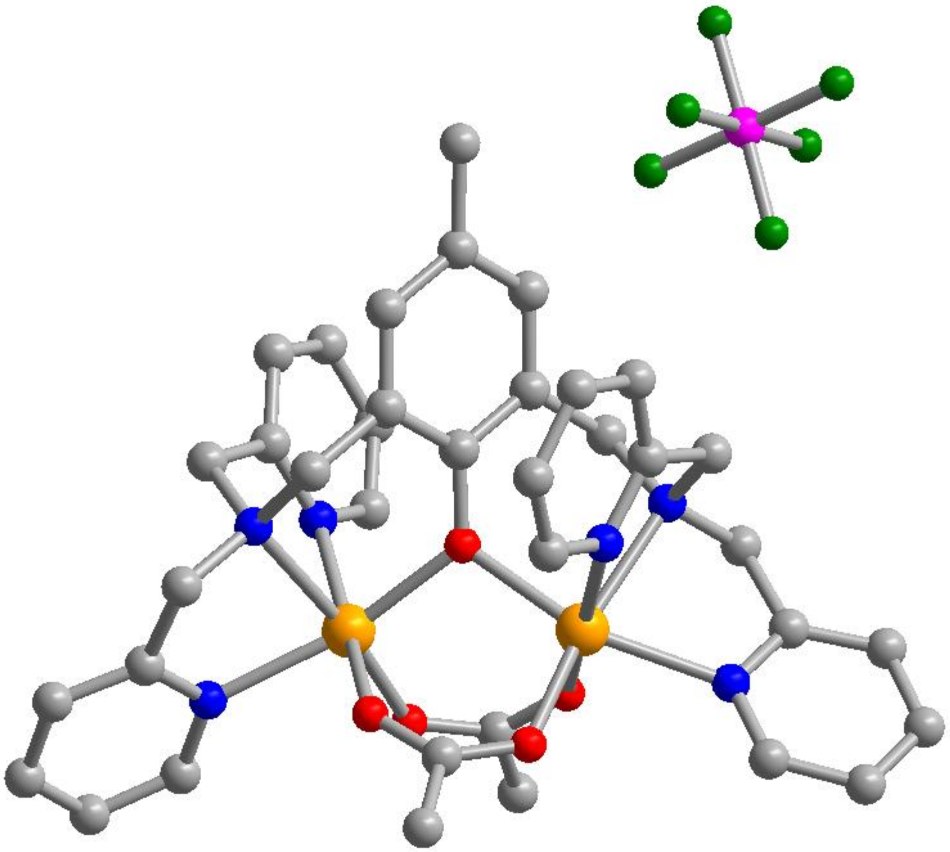

Figure S1. Crystal structure of **1′** with solvent molecules removed for clarity. Orange, pink, green, red, blue, and gray spheres represent Fe, P, F, O, N, and C atoms, respectively; H atoms are omitted for clarity.

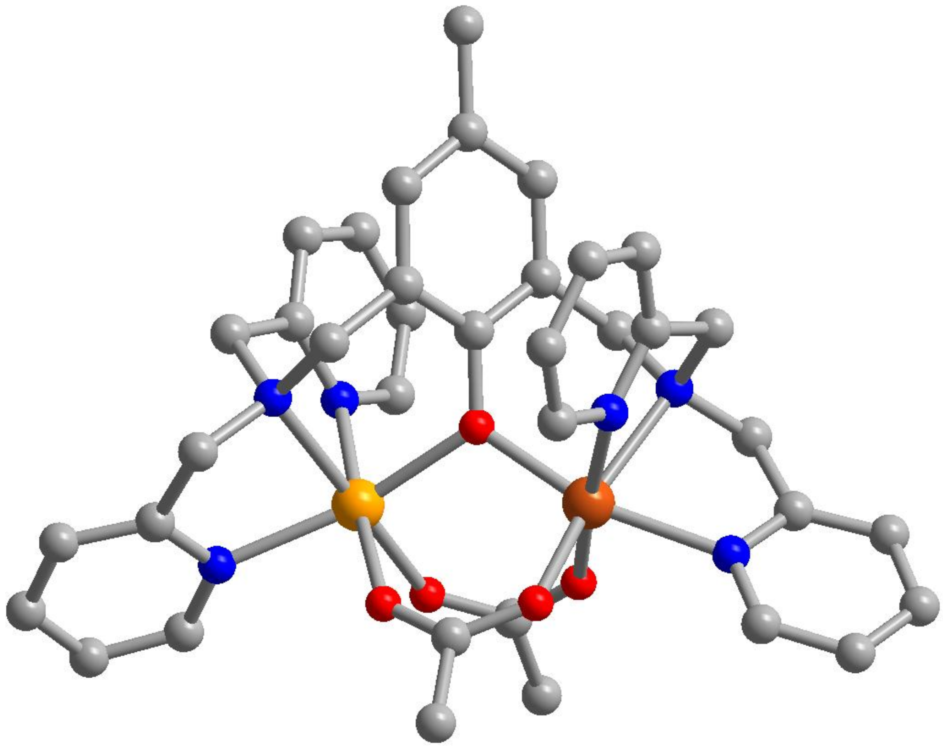

Figure S2. Crystal structure of the cationic complex $[(BPMP)Fe_2(OAc)_2]^{2+}$, as observed in **1-ox′**. Orange, red, blue, and gray spheres represent Fe, O, N, and C atoms, respectively; H atoms are omitted for clarity. Note that the different shades of orange, light and dark, represent Fe centers in different oxidation states, Fe^{II} and Fe^{III}, respectively.

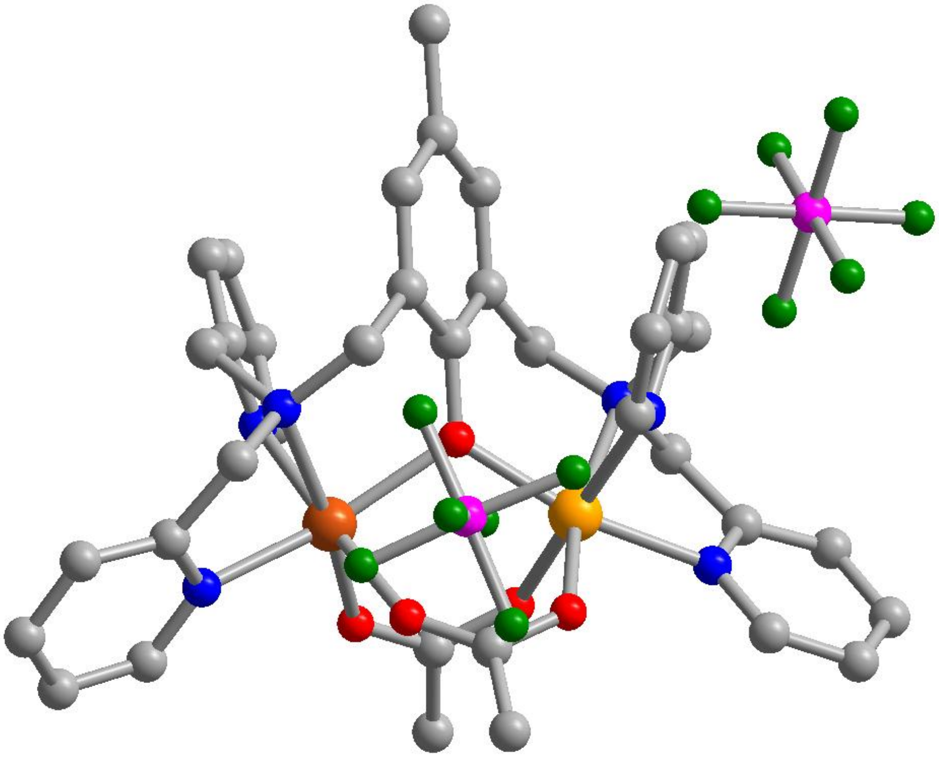

Figure S3. Crystal structure of **1-ox′** with solvent molecules removed for clarity. Orange, pink, green, red, blue, and gray spheres represent Fe, P, F, O, N, and C atoms, respectively; H atoms are omitted for clarity. Note that the different shades of orange, light and dark, represent Fe centers in different oxidation states, Fe^{II} and Fe^{III}, respectively.

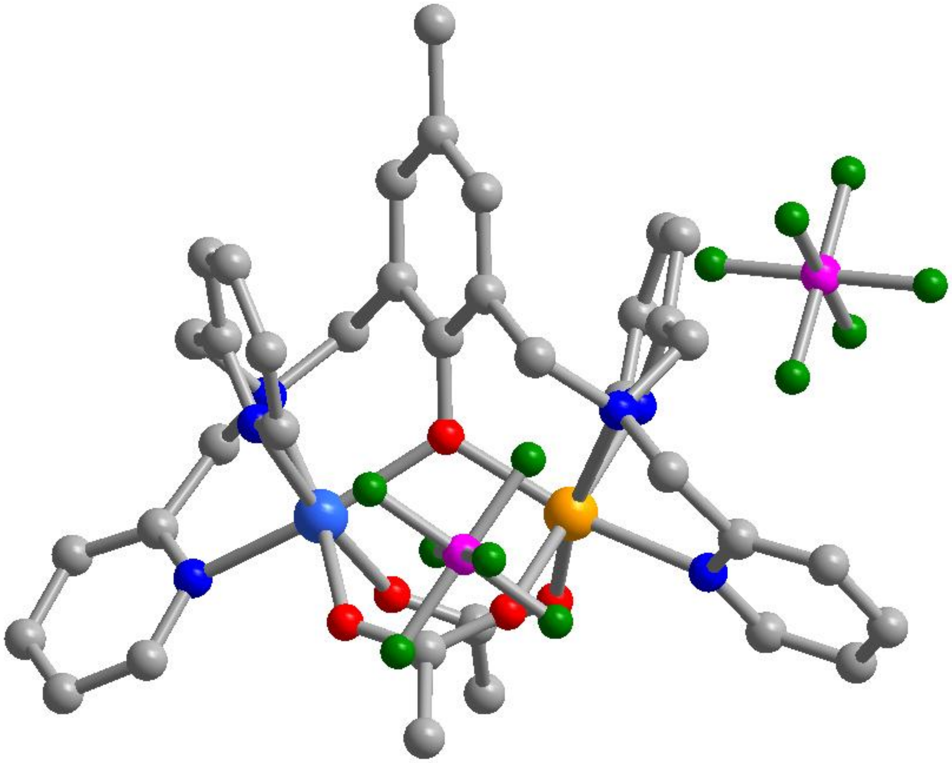

Figure S4. Crystal structure of **2**. Light blue, orange, pink, green, red, blue, and gray spheres represent Zn, Fe, P, F, O, N, and C atoms, respectively; H atoms are omitted for clarity.

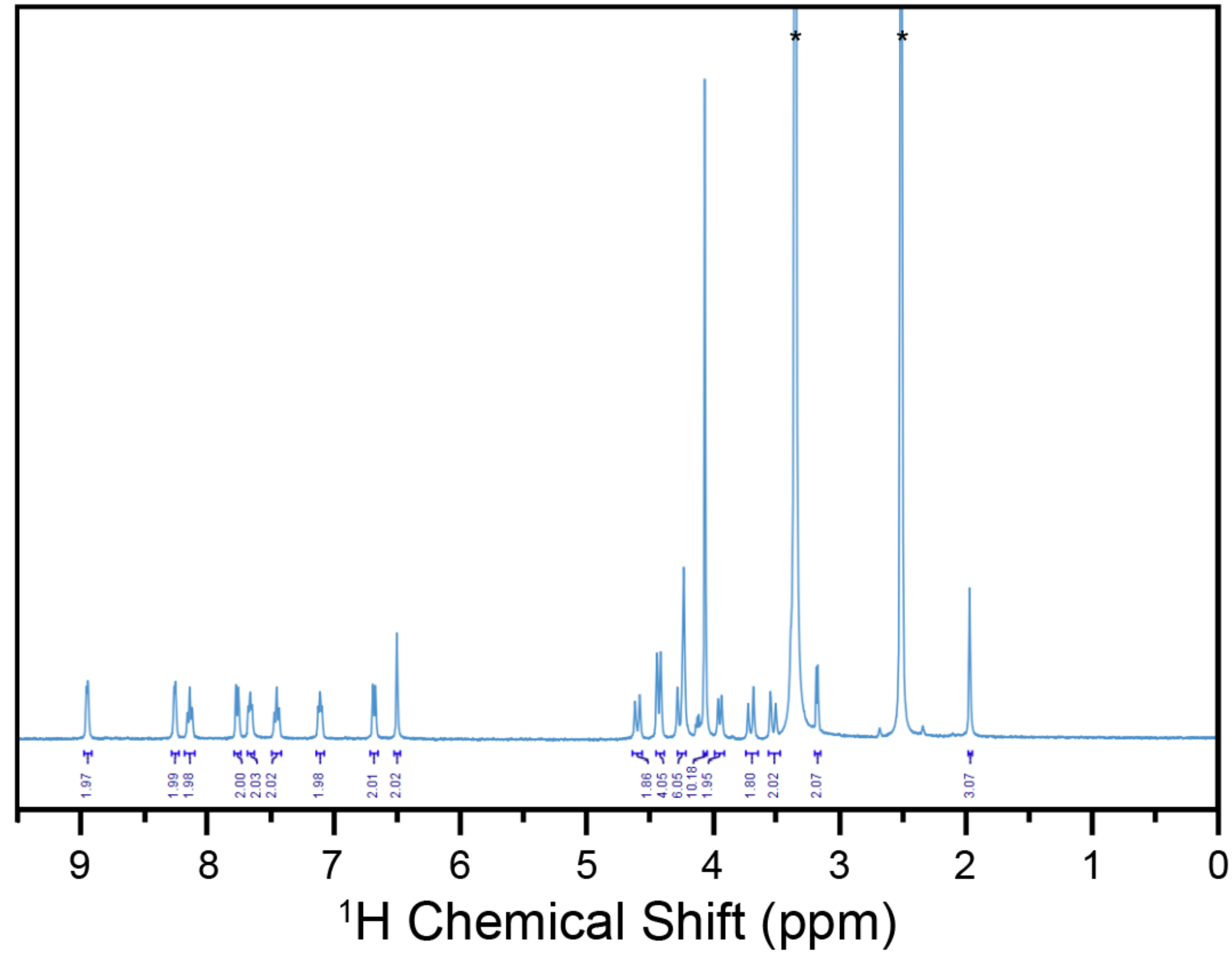

Figure S5. ¹H NMR spectrum of **3** in $(CD_3)_2SO$. The asterisks denote peaks from residual solvents.

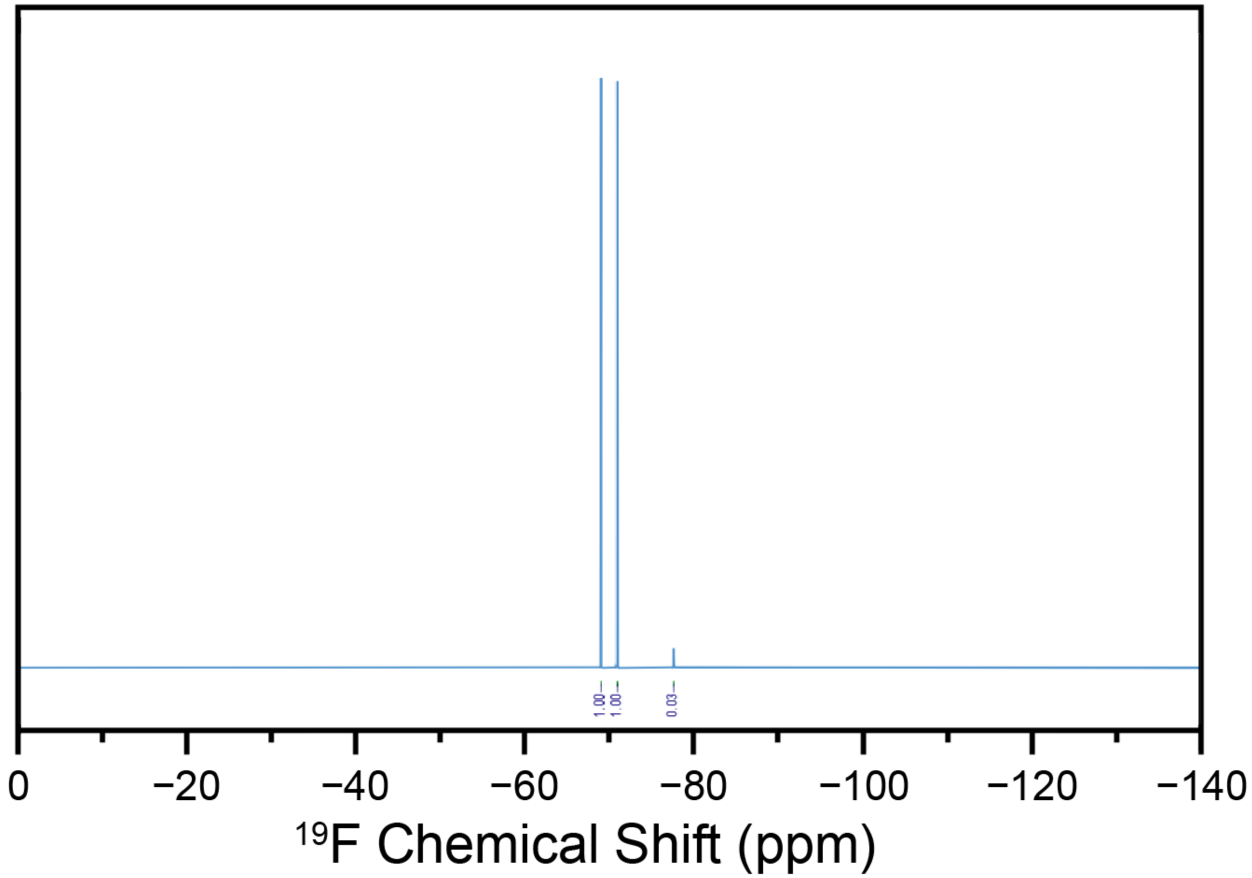

Figure S6. $^{19}F\{^1H\}$ NMR spectrum of **3** in $(CD_3)_2SO$.

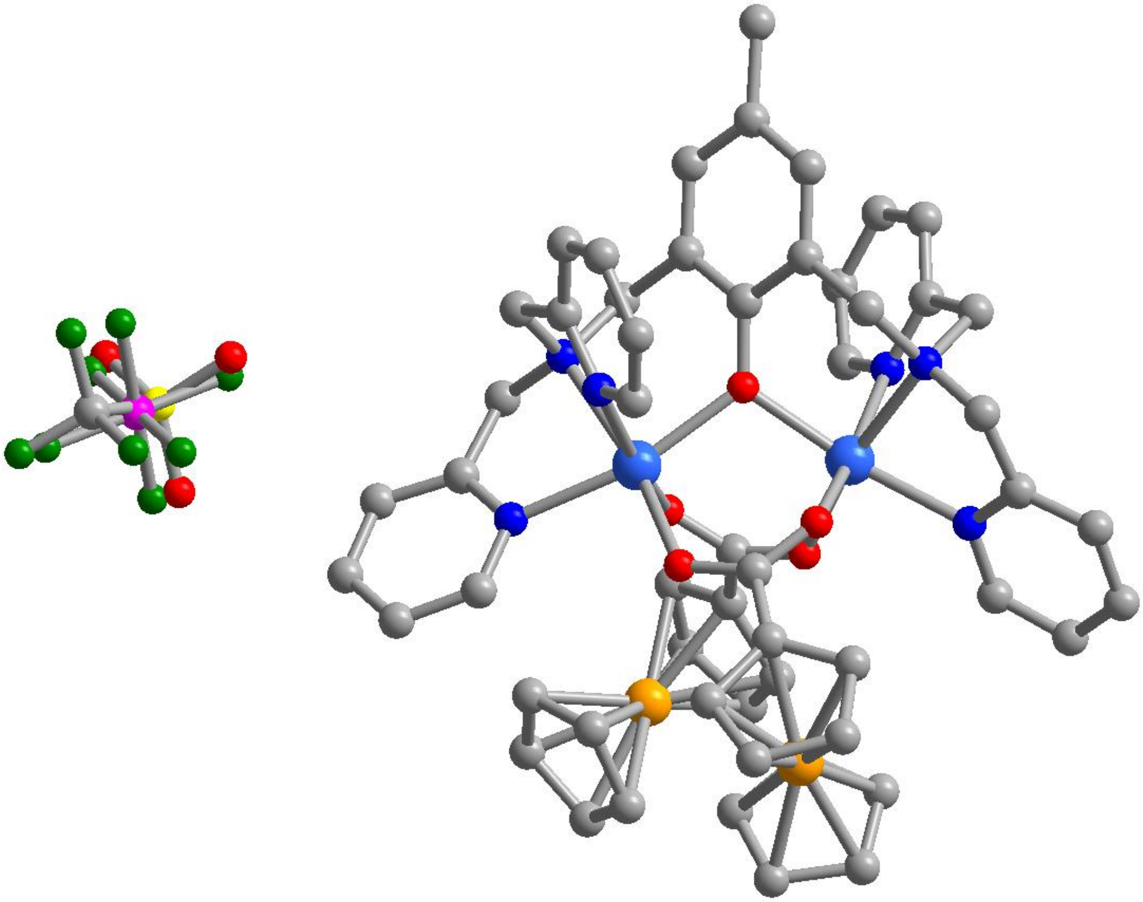

Figure S7. Crystal structure of **3′** with solvent molecules removed for clarity. Light blue, orange, yellow, pink, green, red, blue, and gray spheres represent Zn, Fe, S, P, F, O, N, and C atoms, respectively; H atoms are omitted for clarity. Note that the counteranion is a disordered mixture of $(PF_6)^-$ and $(OTf)^-$.

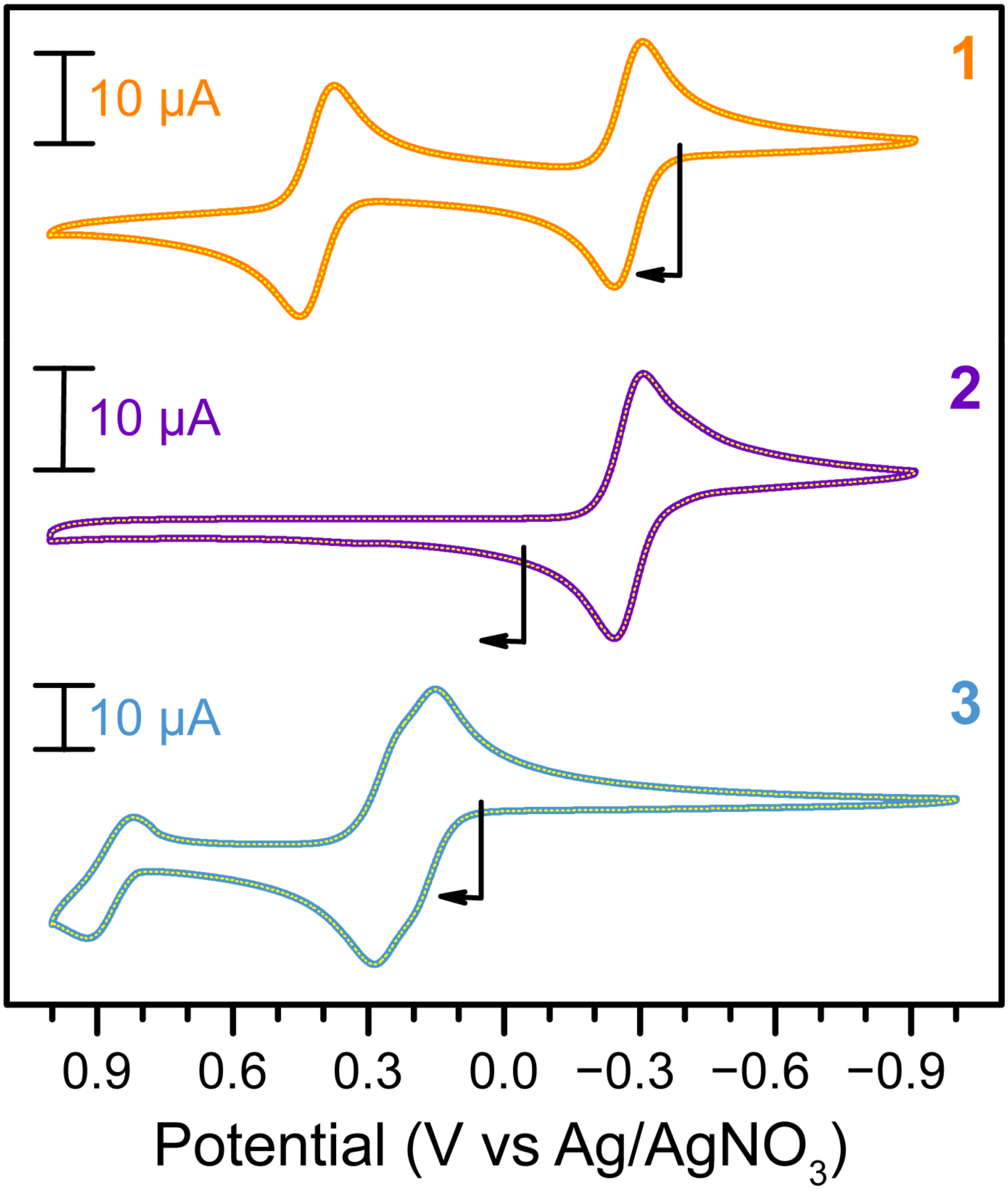

Figure S8. Comparison of CVs of 0.9 mM of **1**, 0.9 mM of **2**, and 0.8 mM of **3** in MeCN containing 0.1 M ($^{n}Bu_4N$)(PF_6) supporting electrolyte collected at ambient temperature (23–25 °C) using 100 mV s^{-1} scan rate. Vertical black lines and arrows denote the open-circuit potentials and scan direction, respectively. Orange, purple, and blue voltammogram traces were collected without applying iR_u compensation, whereas the overlaid yellow dashed lines represent the voltammograms after applying 100% manual iR_u compensation as described in the Experimental section. Glassy carbon, Ag/$AgNO_3$, and Pt mesh were used as working, reference, and counter electrodes, respectively.

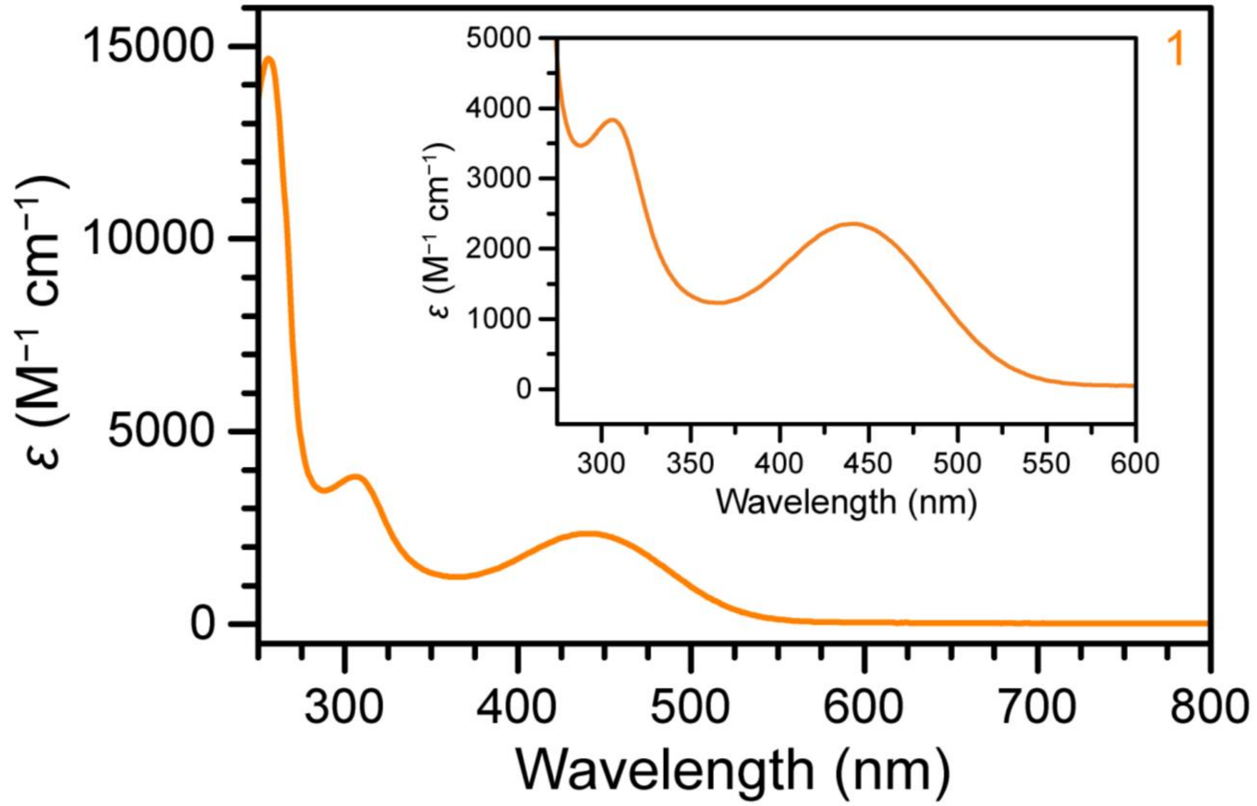

Figure S9. UV–visible absorption spectrum of 0.1 mM of **1** in MeCN. Note that the molar absorptivity (*ε*) is plotted against wavelength.

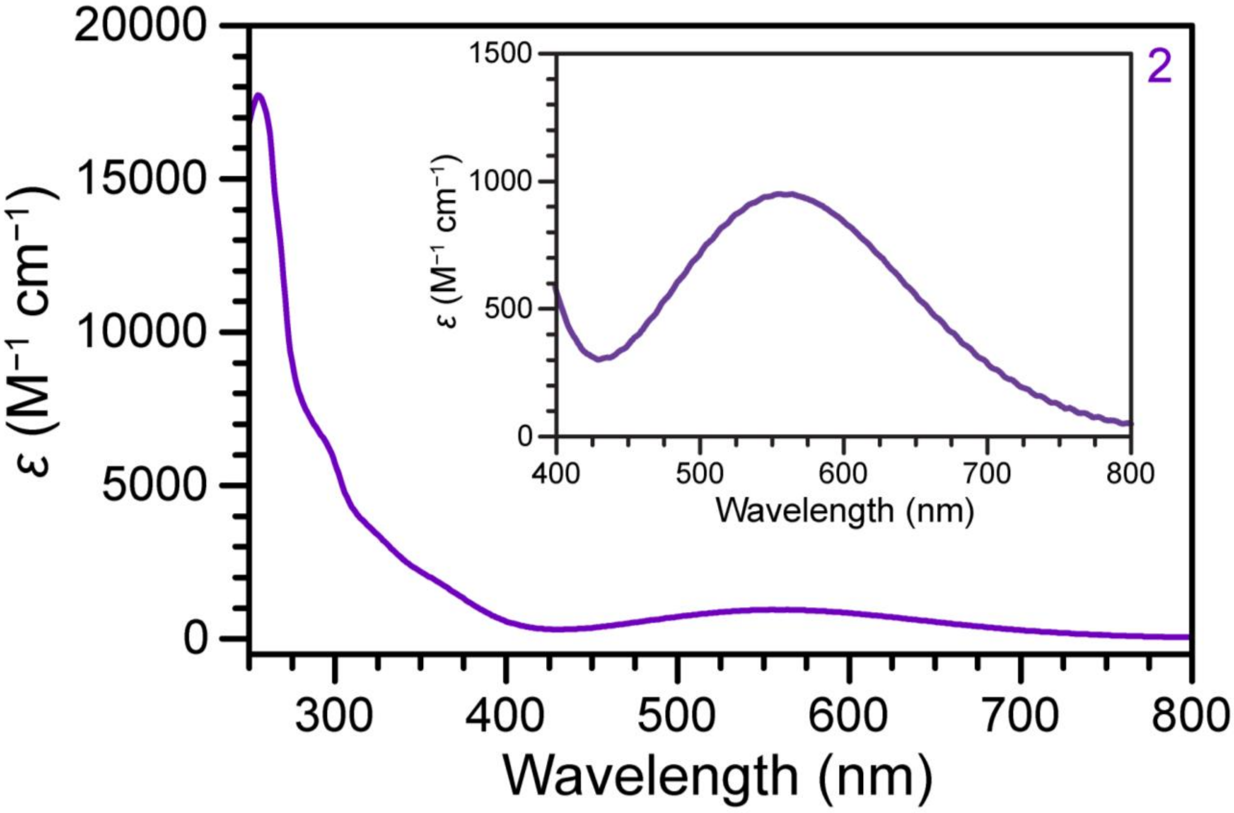

Figure S10. UV–visible absorption spectrum of 0.1 mM of **2** in MeCN. Note that the molar absorptivity (*ε*) is plotted against wavelength.

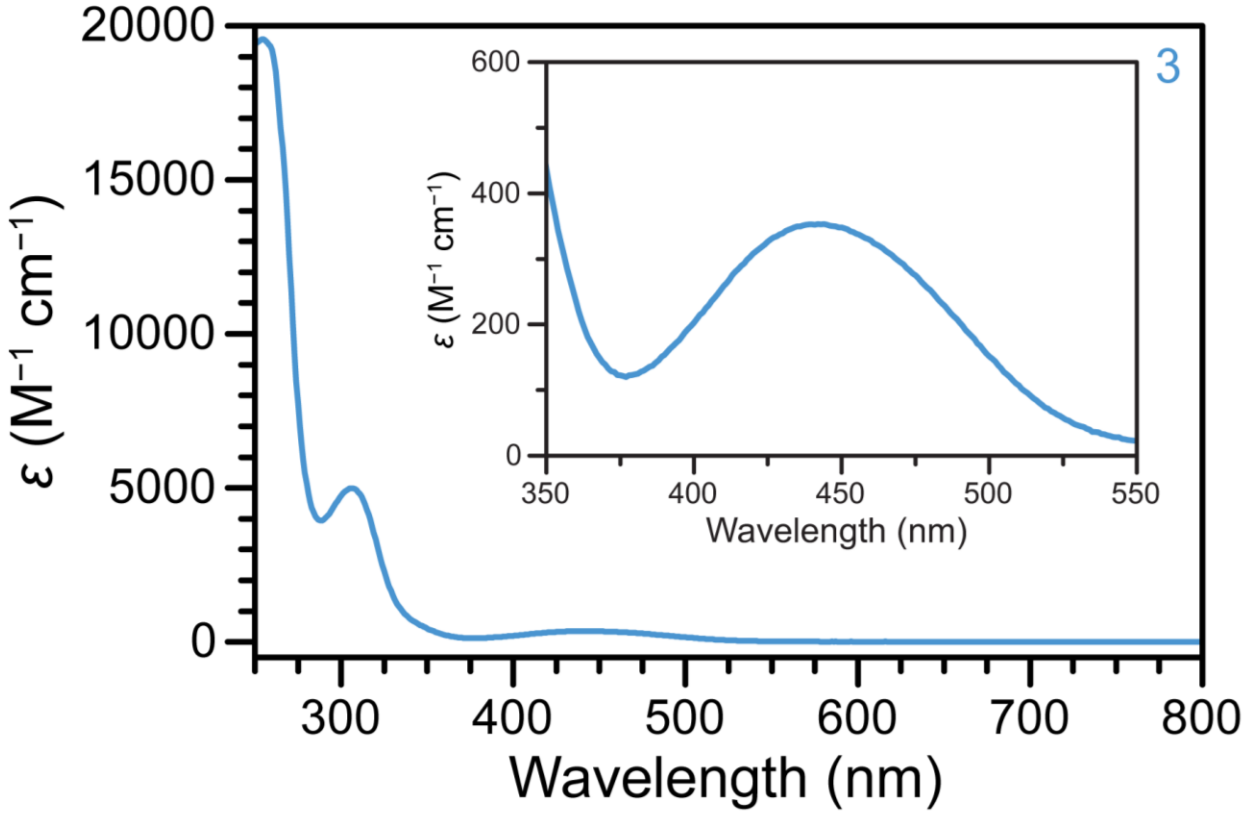

Figure S11. UV–visible absorption spectrum of 0.1 mM of **3** in MeCN. Note that the molar absorptivity (*ε*) is plotted against wavelength.

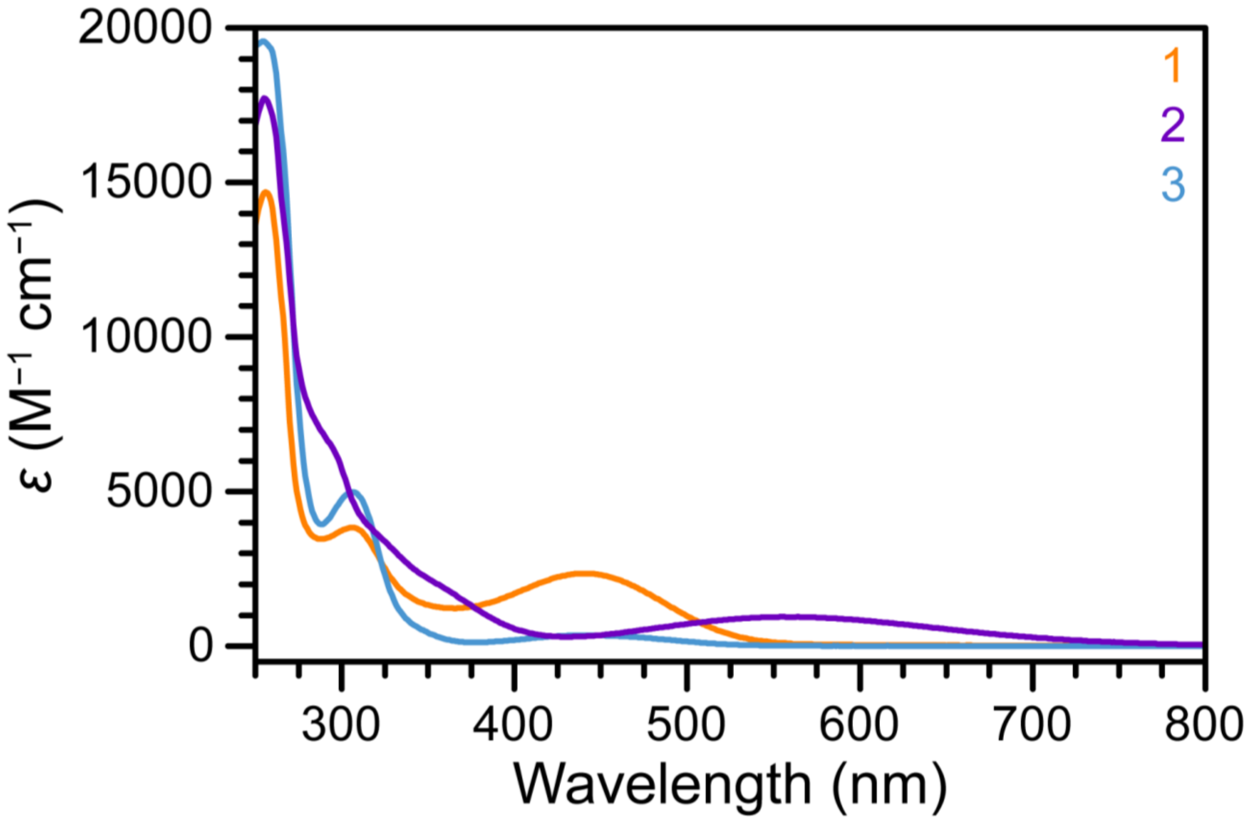

Figure S12. Comparison of UV–visible absorption spectra of **1**, **2**, and **3** in MeCN. Note that the molar absorptivity (*ε*) is plotted against wavelength.

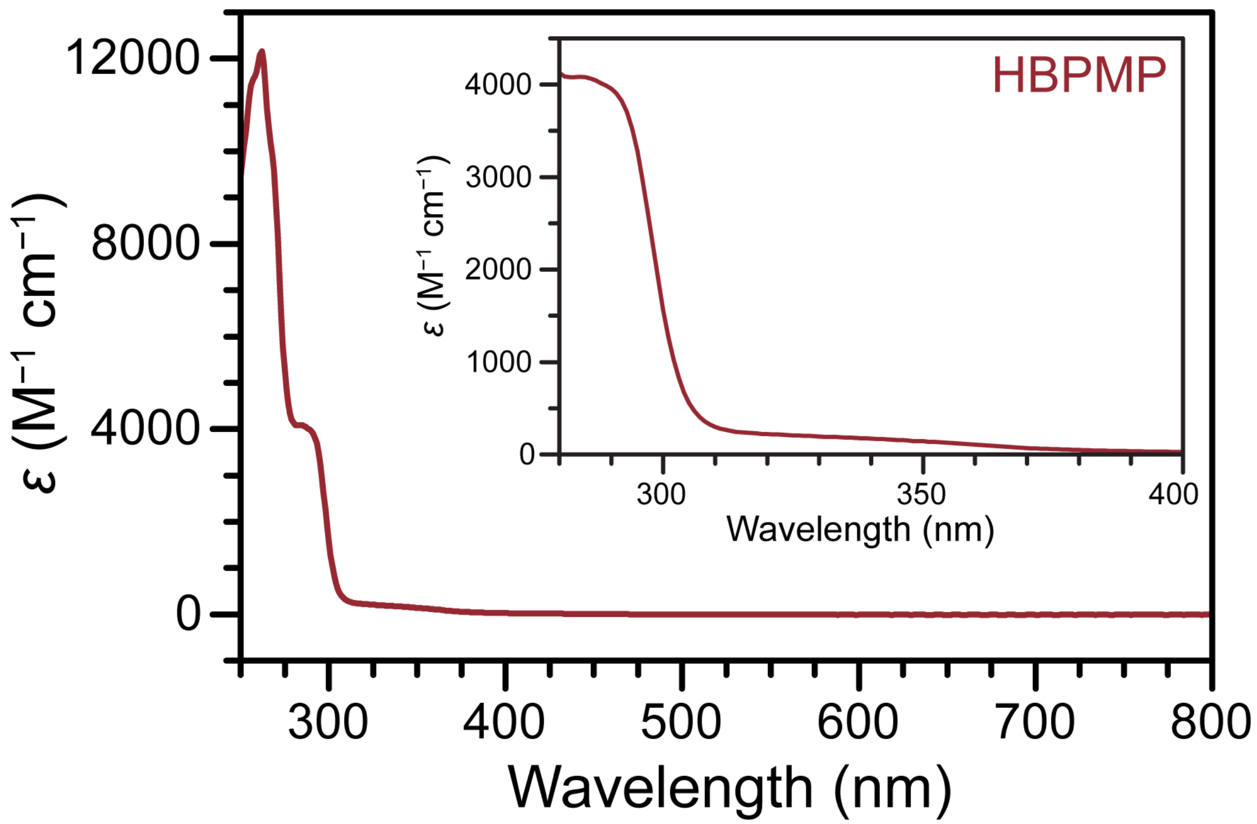

Figure S13. UV–visible absorption spectrum of 0.2 mM of HBPMP ligand in MeCN. Note that the molar absorptivity (ε) is plotted against wavelength.

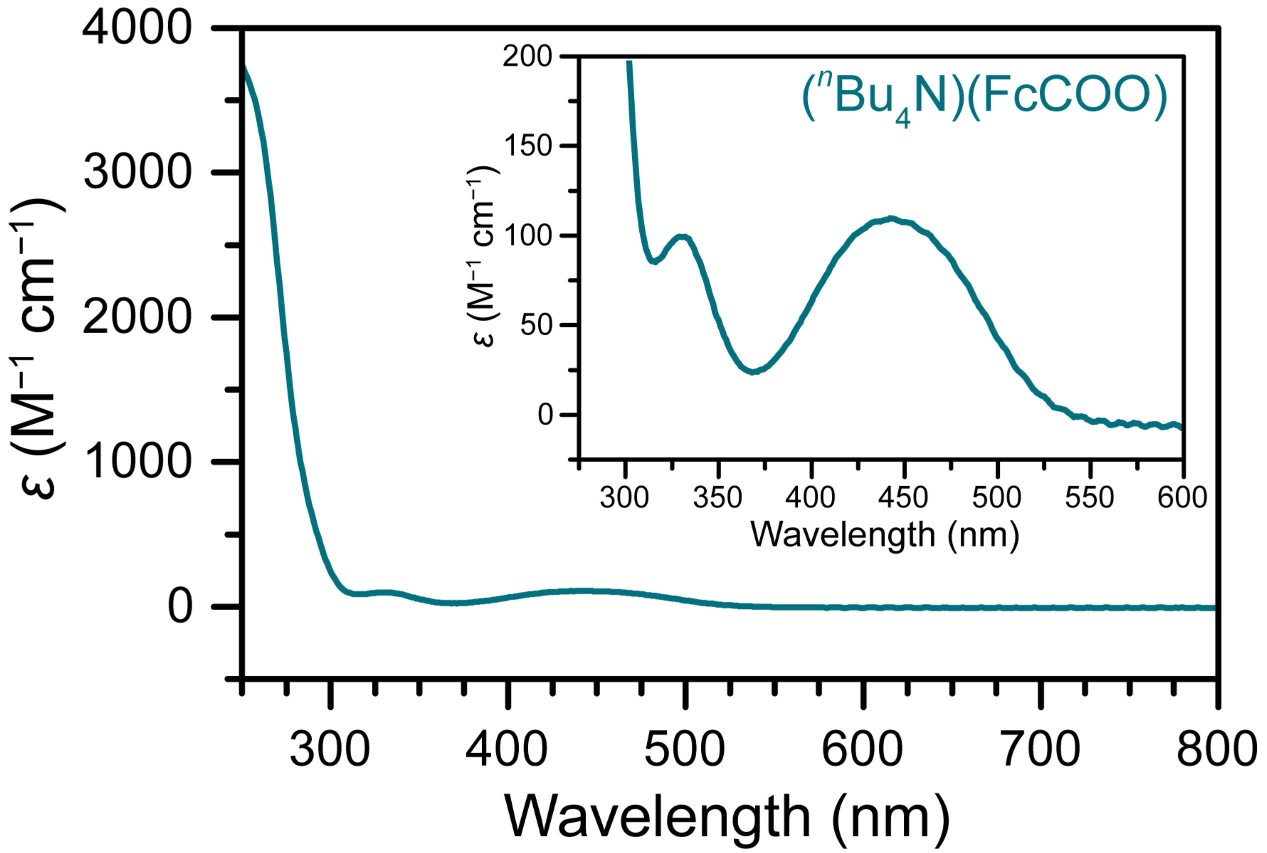

Figure 14. UV–visible absorption spectrum of 0.3 mM of (^{n}Bu$_4$N)(FcCOO) in MeCN. Note that the molar absorptivity (*ε*) is plotted against wavelength.

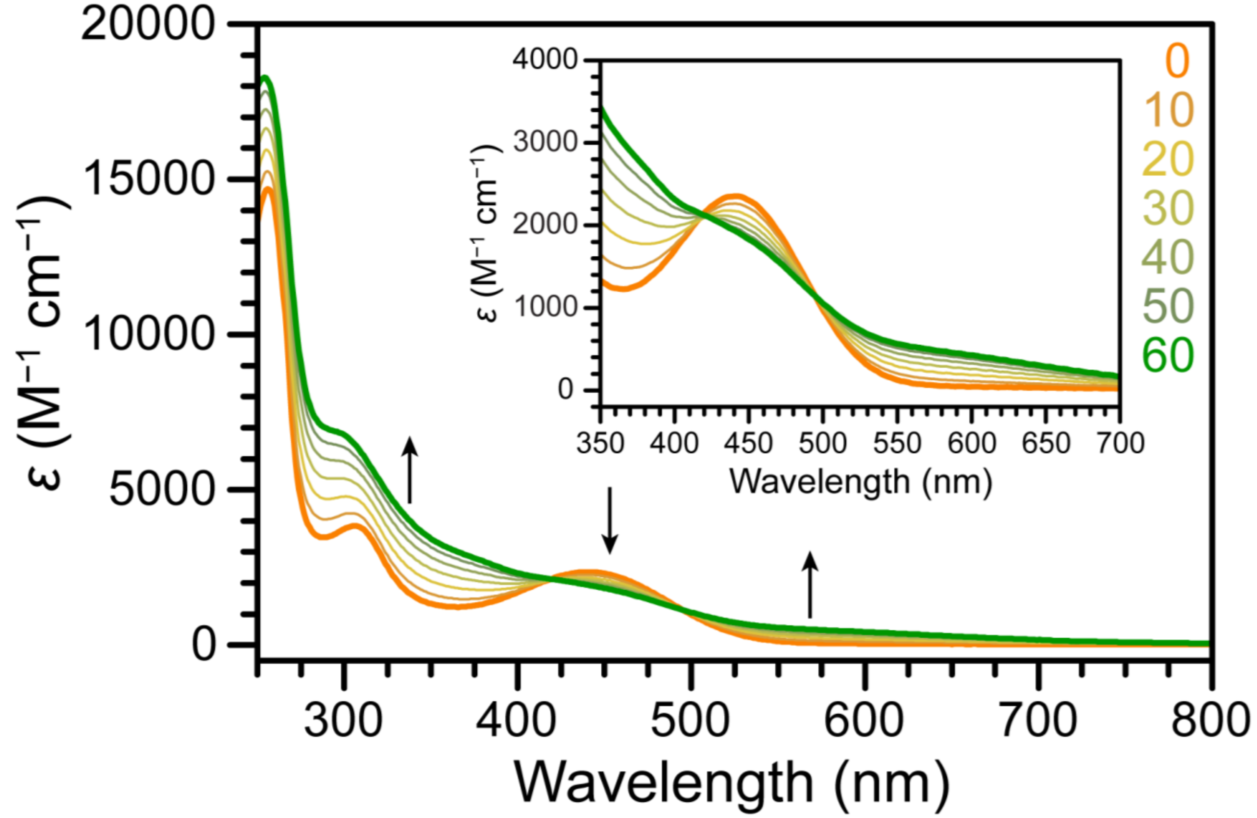

Figure S15. UV–visible absorption spectra of 0.1 mM of **1** in MeCN in the absence of O_2 (orange) and upon exposure to ambient air for variable time (10–60 minutes). The legend denotes the number of minutes that the sample was exposed to air prior to data collection. The black arrows indicate the changes in spectral features that occur upon air exposure. Note that the molar absorptivity (ε) is plotted against wavelength.

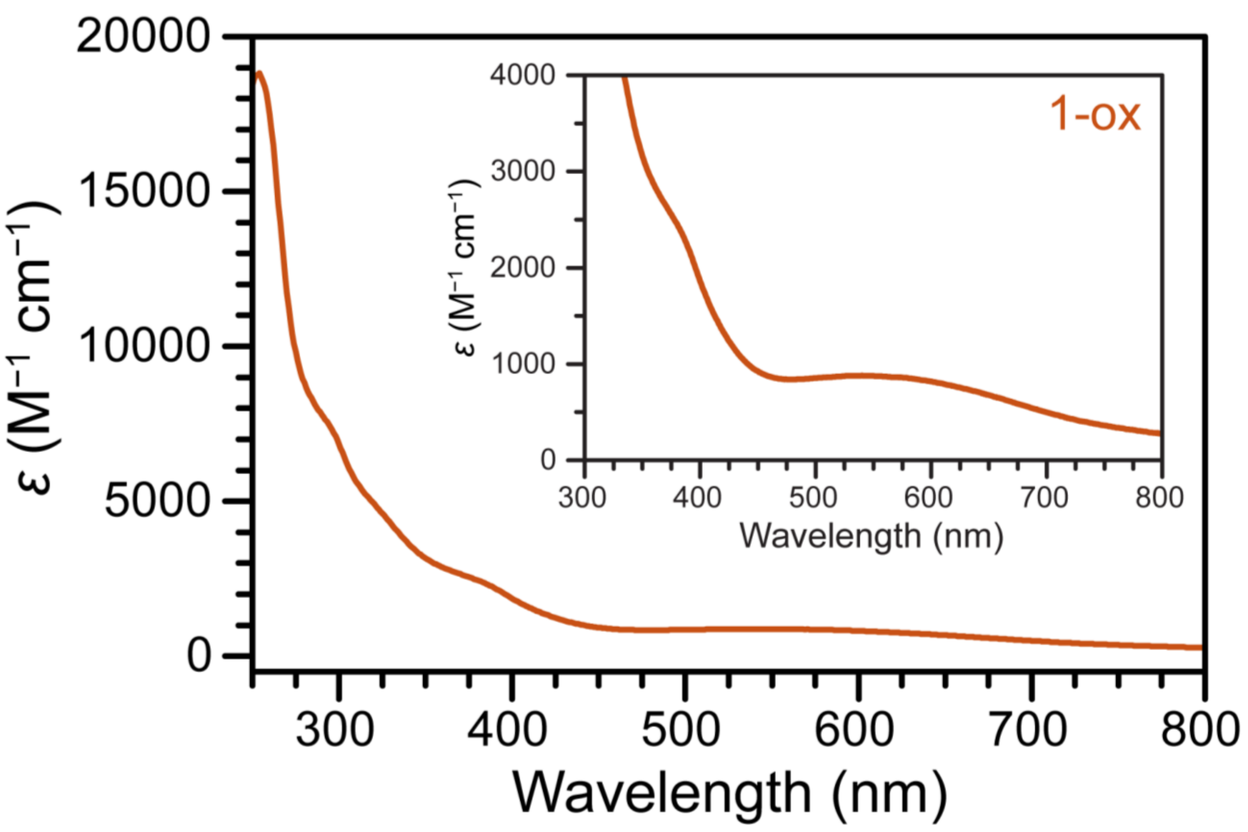

Figure S16. UV–visible absorption spectrum of 0.1 mM of **1-ox** in MeCN. Note that the molar absorptivity (ε) is plotted against wavelength.

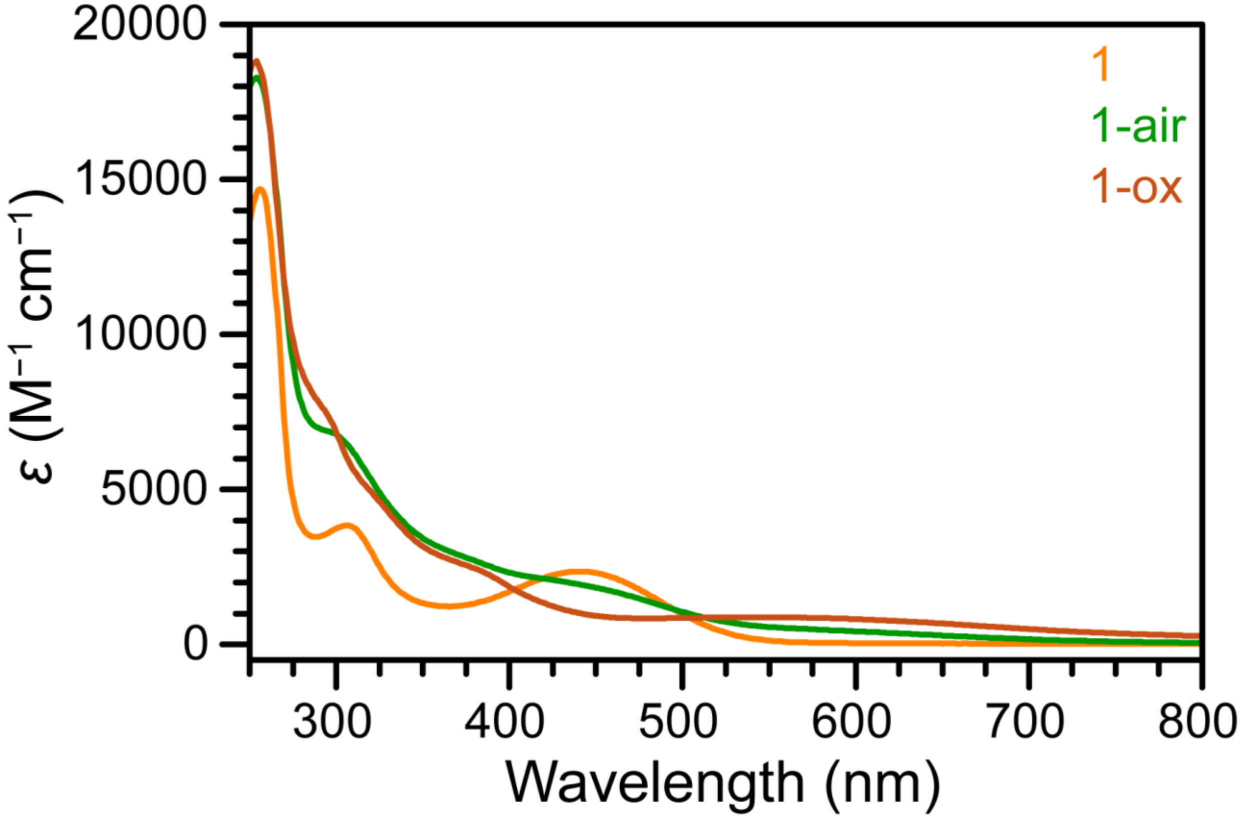

Figure S17. Comparison of UV–visible absorption spectra of **1** in the absence of O_2 (orange), **1** after exposure to ambient air for 60 minutes (green), and **1-ox** in MeCN. Note that the molar absorptivity (ε) is plotted against wavelength.

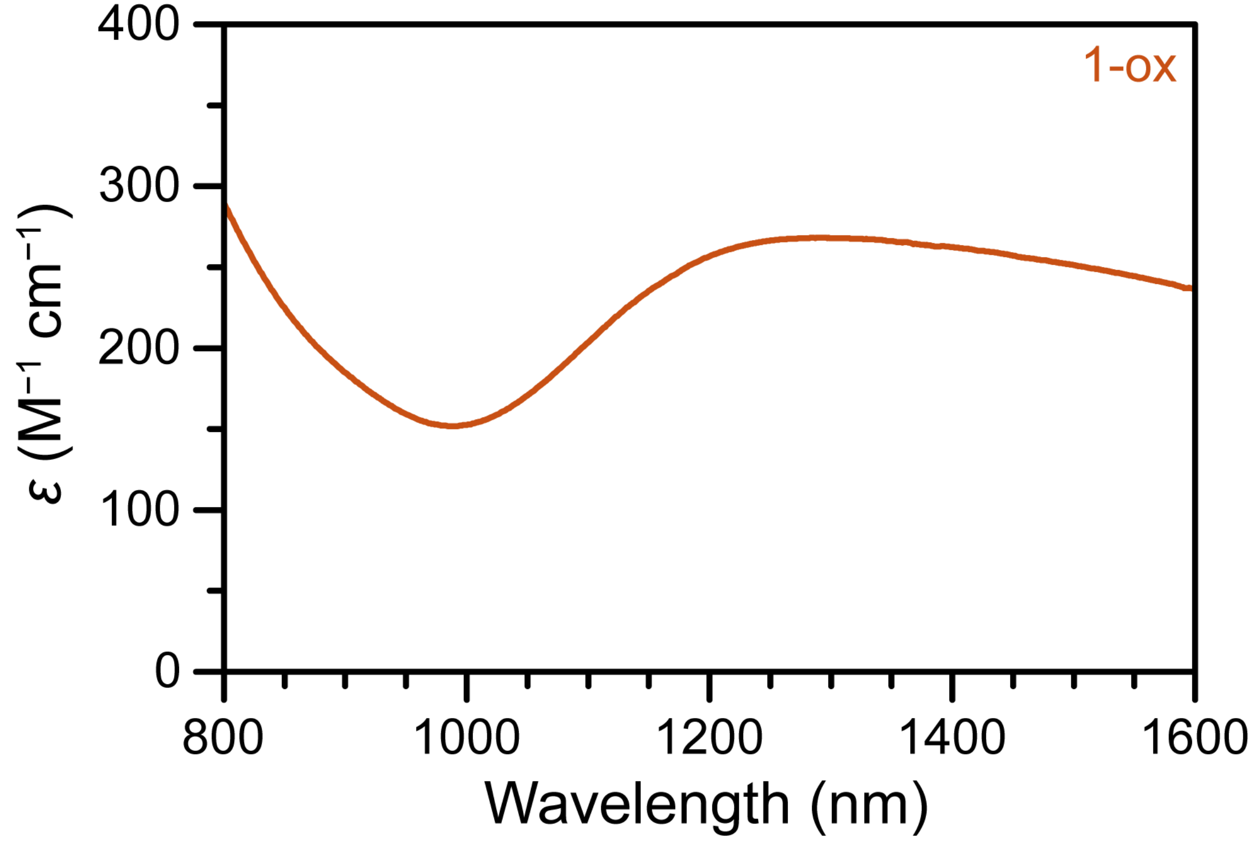

Figure S18. NIR absorption spectrum of 2.9 mM of **1-ox** in MeCN. Note that the molar absorptivity (ε) is plotted against wavelength.

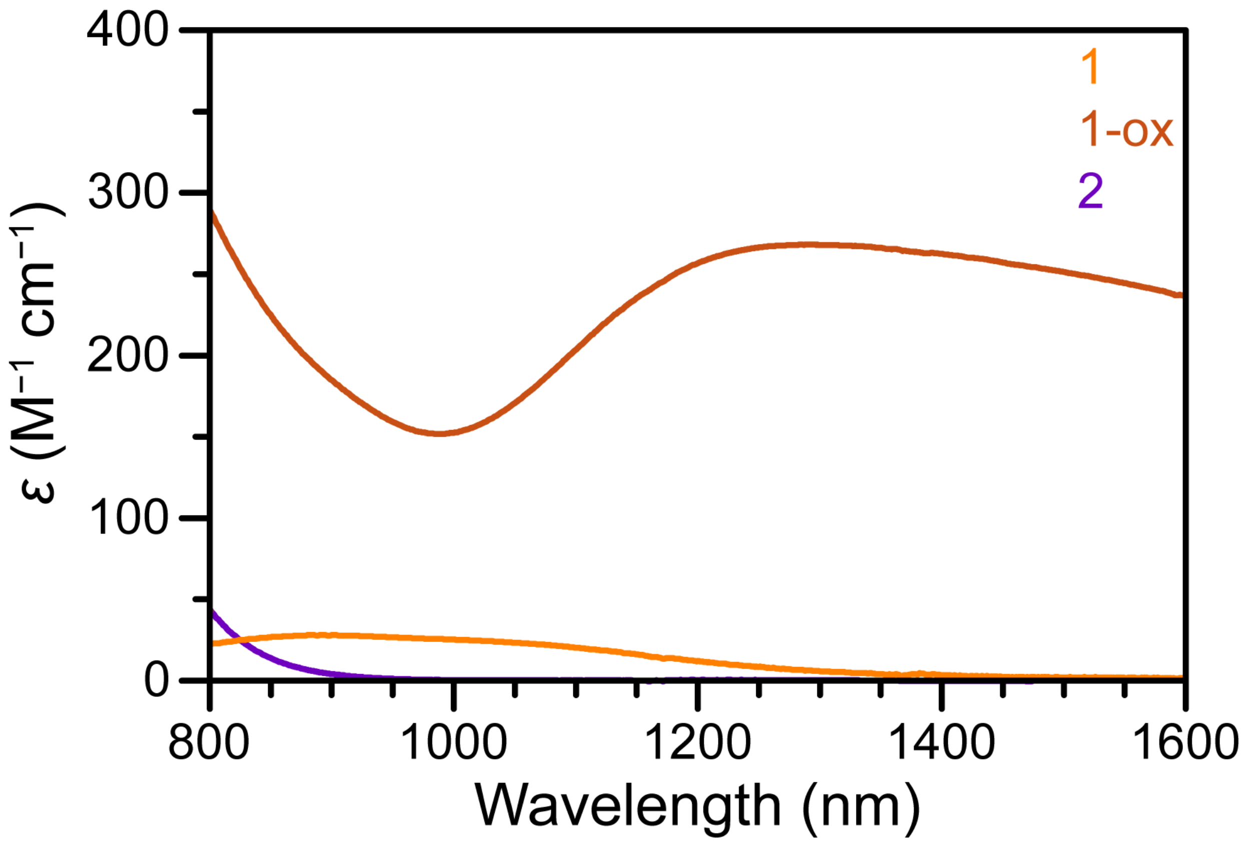

Figure S19. Comparison of NIR absorption spectra of **1**, **1-ox**, and **2** in MeCN. Note that the molar absorptivity (*ε*) is plotted against wavelength.

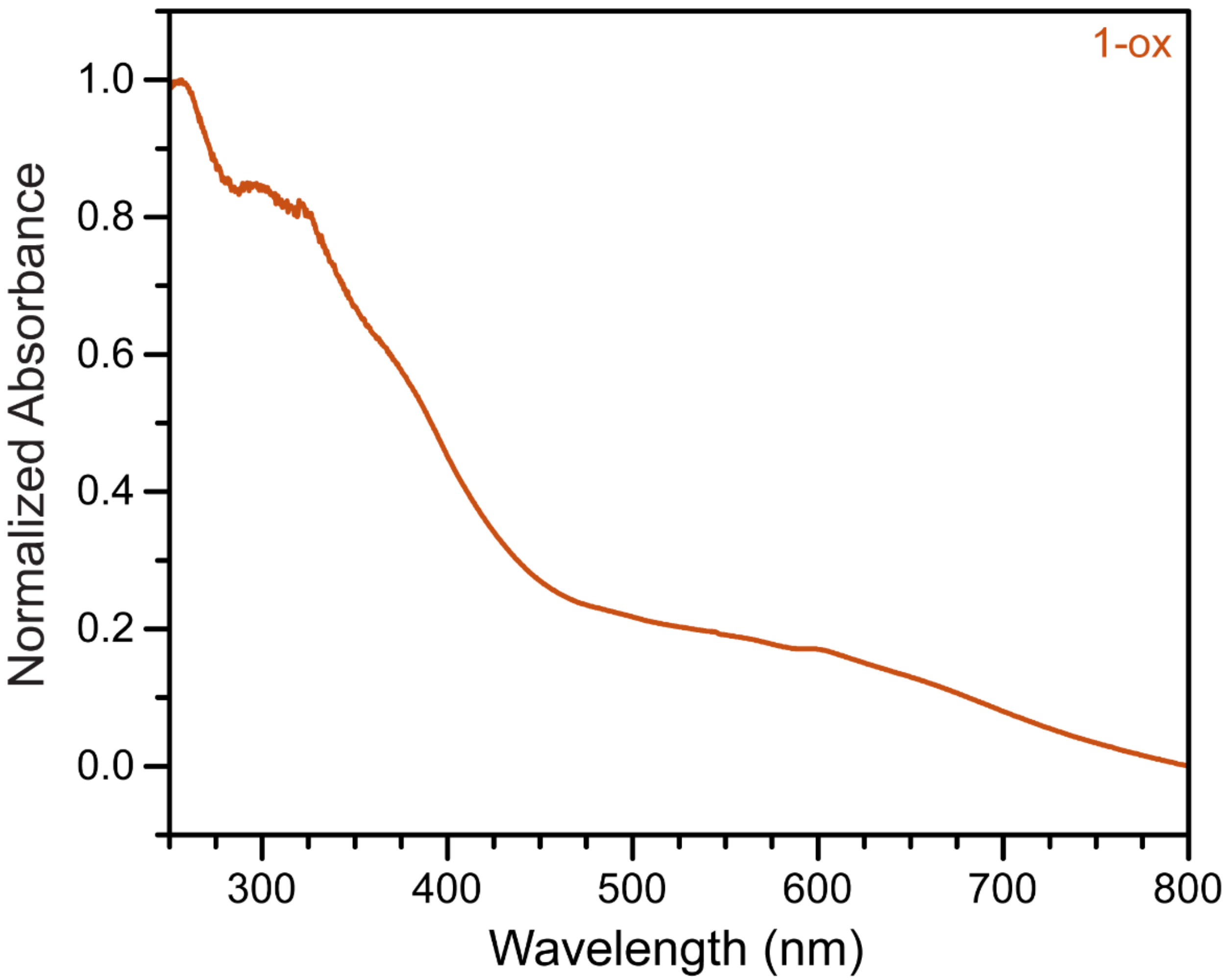

Figure S20. UV–visible absorption spectrum of a solid sample of **1-ox**. Note that the normalized absorbance over the 250–800 nm range is plotted against wavelength.

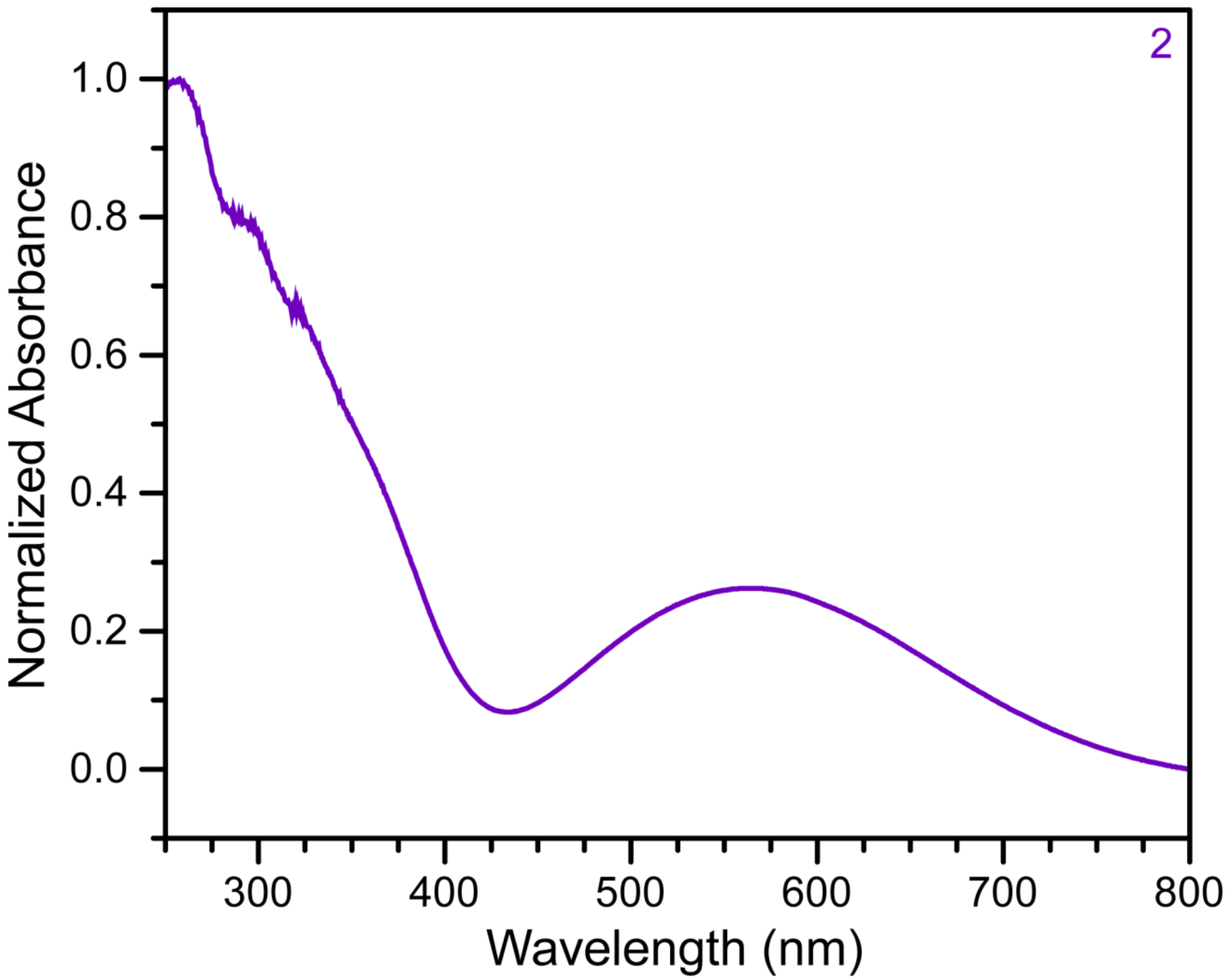

Figure S21. UV–visible absorption spectrum of a solid sample of **2**. Note that the normalized absorbance over the 250–800 nm range is plotted against wavelength.

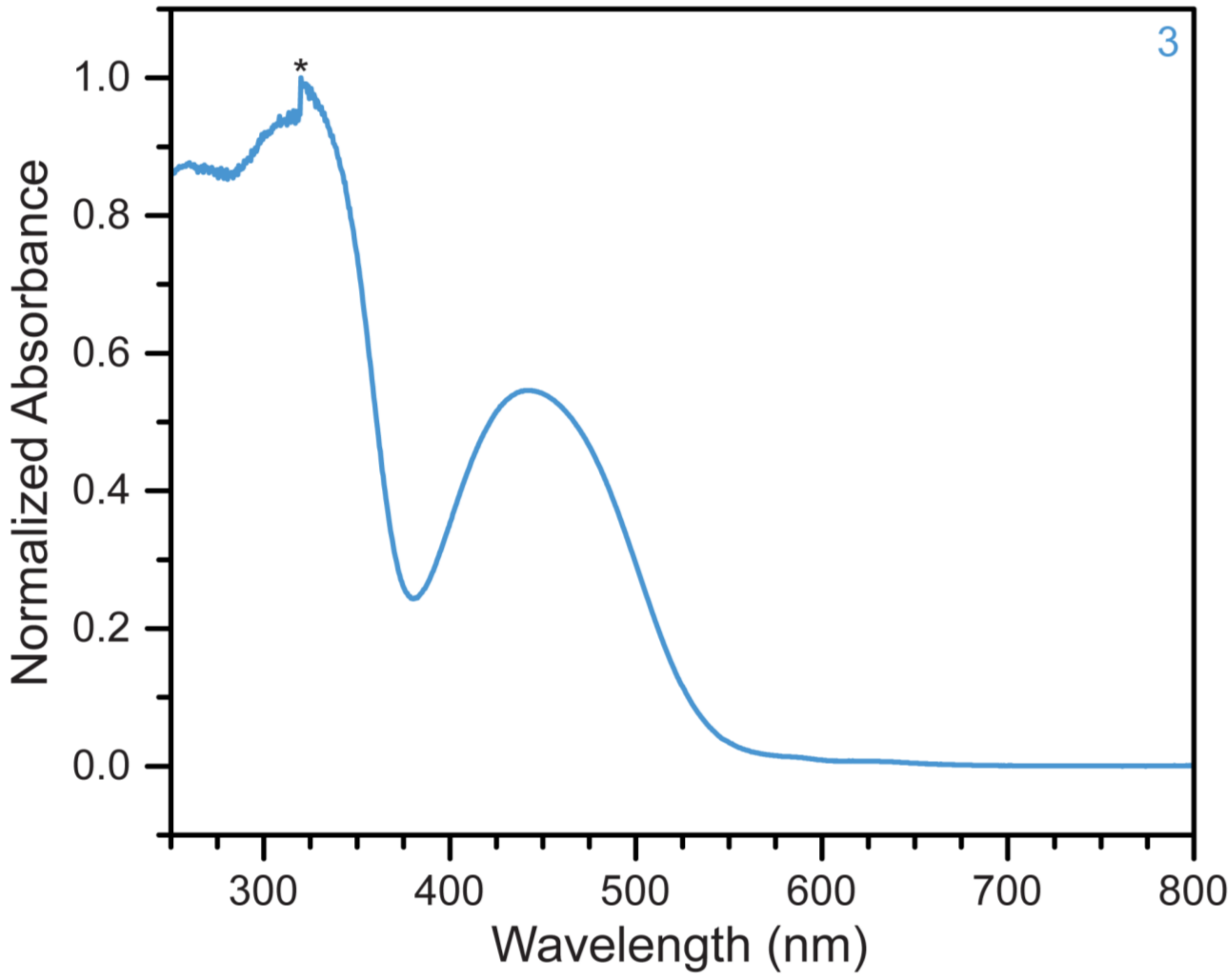

Figure S22. UV–visible absorption spectrum of a solid sample of **3**. Note that the normalized absorbance over the 250–800 nm range is plotted against wavelength. The asterisk denotes an instrumental artifact arising from a detector change at 325 nm.

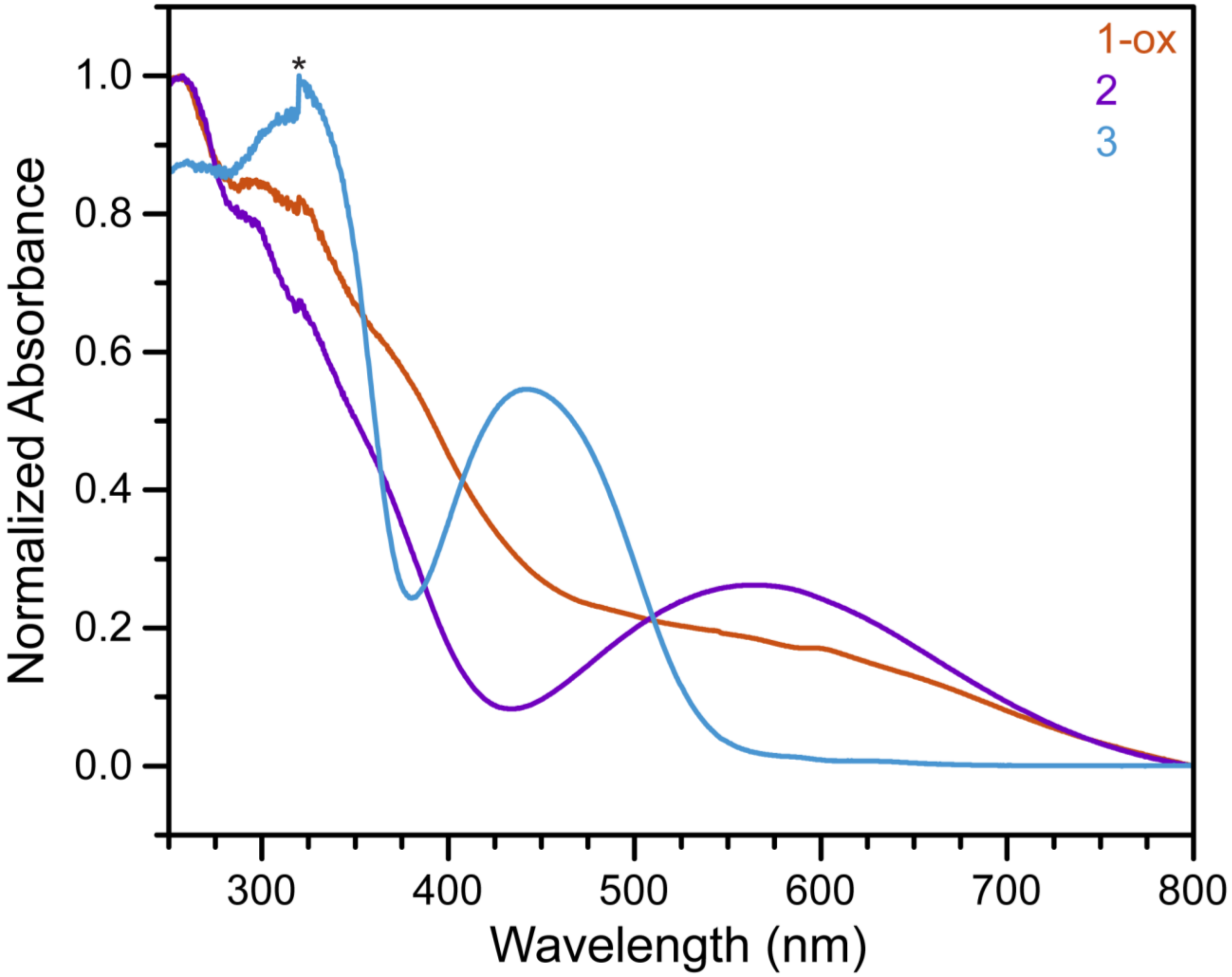

Figure S23. Comparison of UV–visible absorption spectra of solid samples of **1-ox**, **2**, and **3**. Note that the normalized absorbance over the 250–800 nm range for each sample is plotted against wavelength. The asterisk denotes an instrumental artifact arising from a detector change at 325 nm.

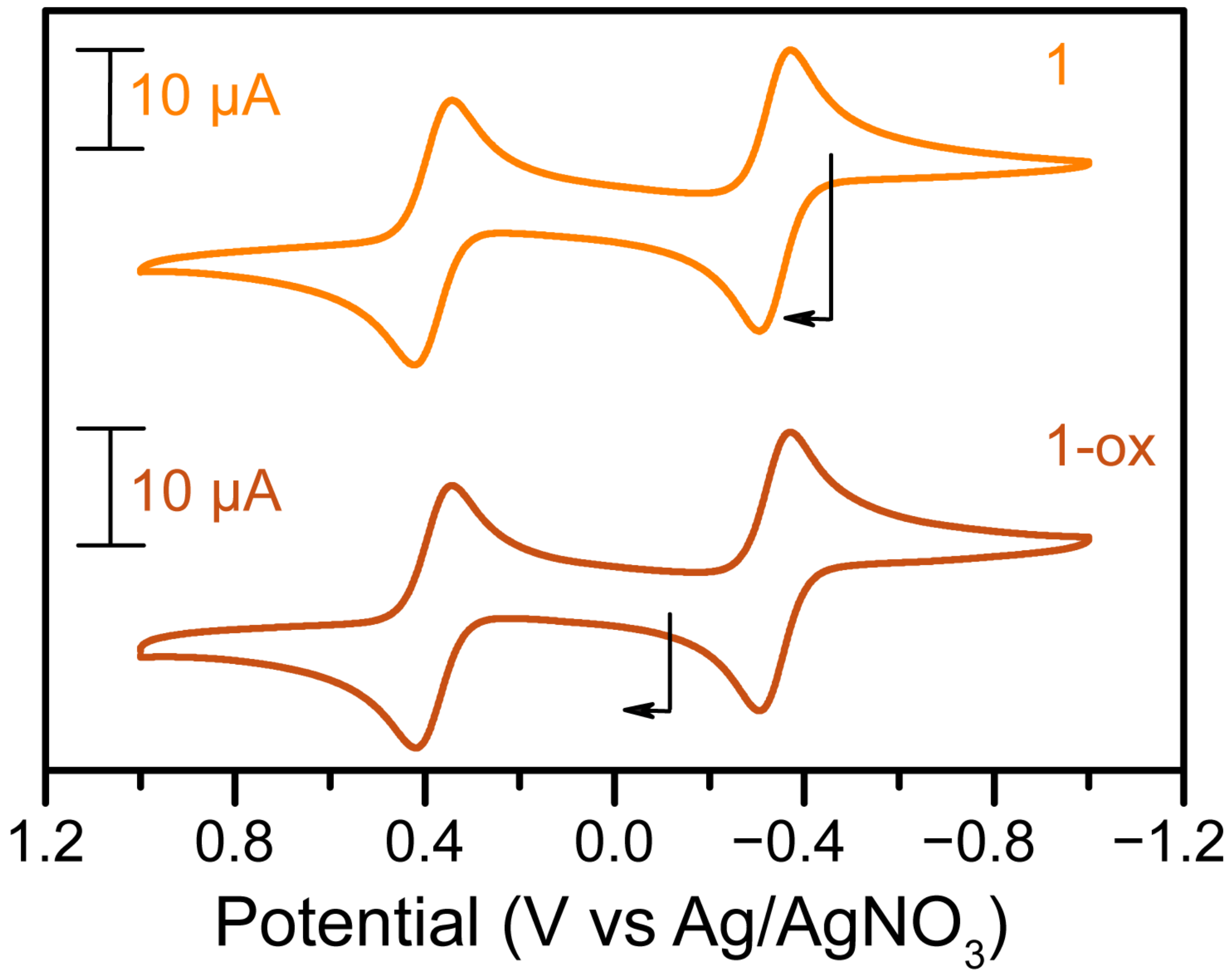

Figure S24. Comparison of CVs of 0.9 mM of **1** and 0.8 mM of **1-ox** in MeCN containing 0.1 M (^{n}Bu$_4$N)(PF$_6$) supporting electrolyte collected at ambient temperature (23–25 °C) using 100 mV s^{-1} scan rate. Vertical black lines and arrows denote the open-circuit potentials and scan direction, respectively. Glassy carbon, Ag/AgNO$_3$, and Pt mesh were used as working, reference, and counter electrodes, respectively.

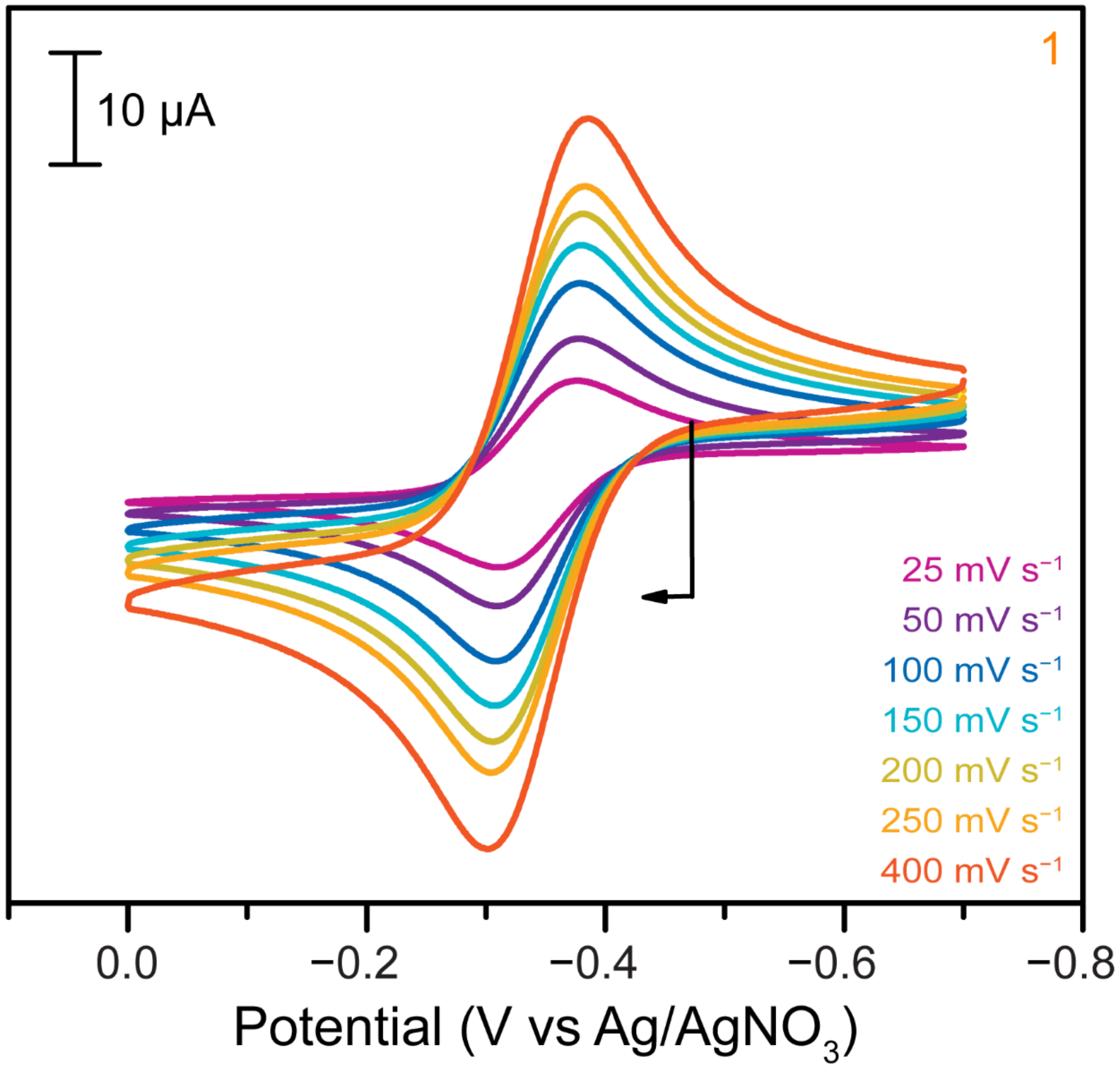

Figure S25. CVs of 1.1 mM of **1** in MeCN containing 0.1 M ($^{n}Bu_4N$)(PF_6) supporting electrolyte collected in the potential window around the $Fe^{II}Fe^{III}/Fe^{II}_2$ redox couple at ambient temperature (23–25 °C) using variable scan rates (25–400 mV s^{-1}). Vertical black line and arrow denote the open-circuit potential and scan direction, respectively. Glassy carbon, Ag/$AgNO_3$, and Pt mesh were used as working, reference, and counter electrodes, respectively.

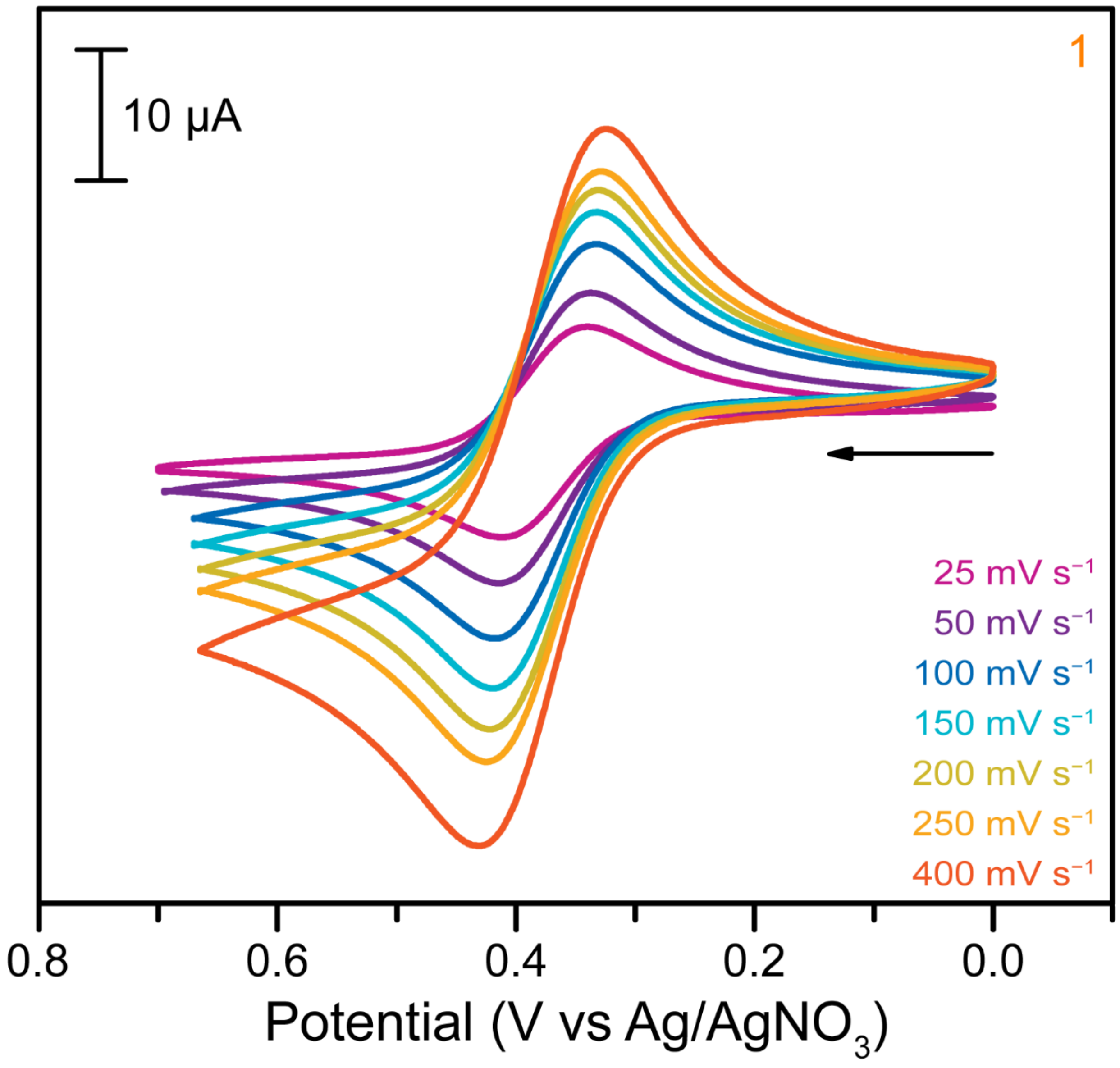

Figure S26. CVs of 1.1 mM of **1** in MeCN containing 0.1 M ($^{n}Bu_4N$)(PF_6) supporting electrolyte collected in the potential window around the $Fe^{III}_2/Fe^{II}Fe^{III}$ redox couple at ambient temperature (23–25 °C) using variable scan rates (25–400 mV s^{-1}). The black arrow denotes the scan direction. Glassy carbon, Ag/$AgNO_3$, and Pt mesh were used as working, reference, and counter electrodes, respectively.

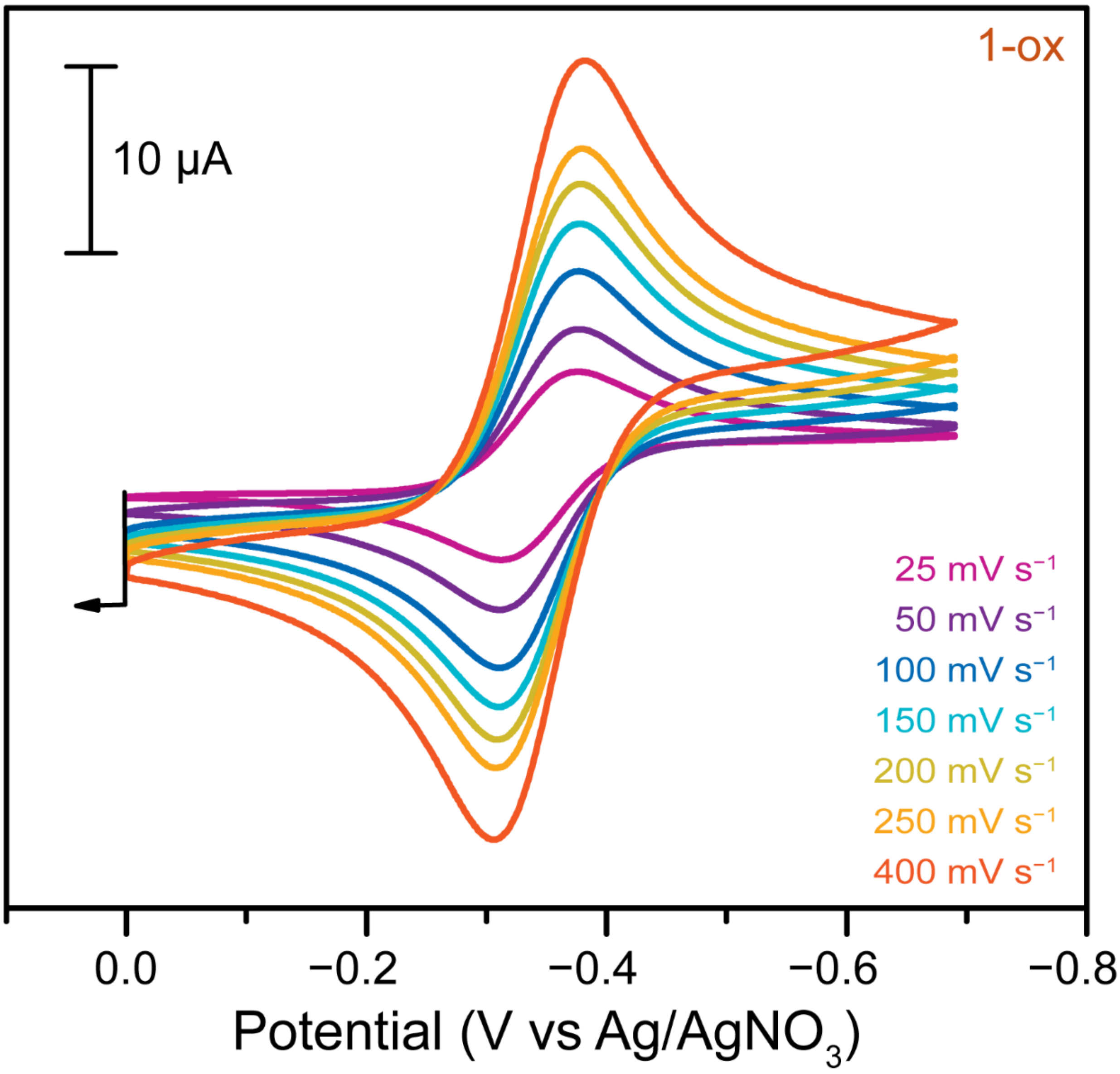

Figure S27. CVs of 0.7 mM of **1-ox** in MeCN containing 0.1 M ($^{n}Bu_4N$)(PF_6) supporting electrolyte collected in the potential window around the $Fe^{II}Fe^{III}/Fe^{II}_2$ redox couple at ambient temperature (23–25 °C) using variable scan rates (25–400 mV s^{-1}). Vertical black line and arrow denote the open-circuit potential and scan direction, respectively. Glassy carbon, Ag/$AgNO_3$, and Pt mesh were used as working, reference, and counter electrodes, respectively.

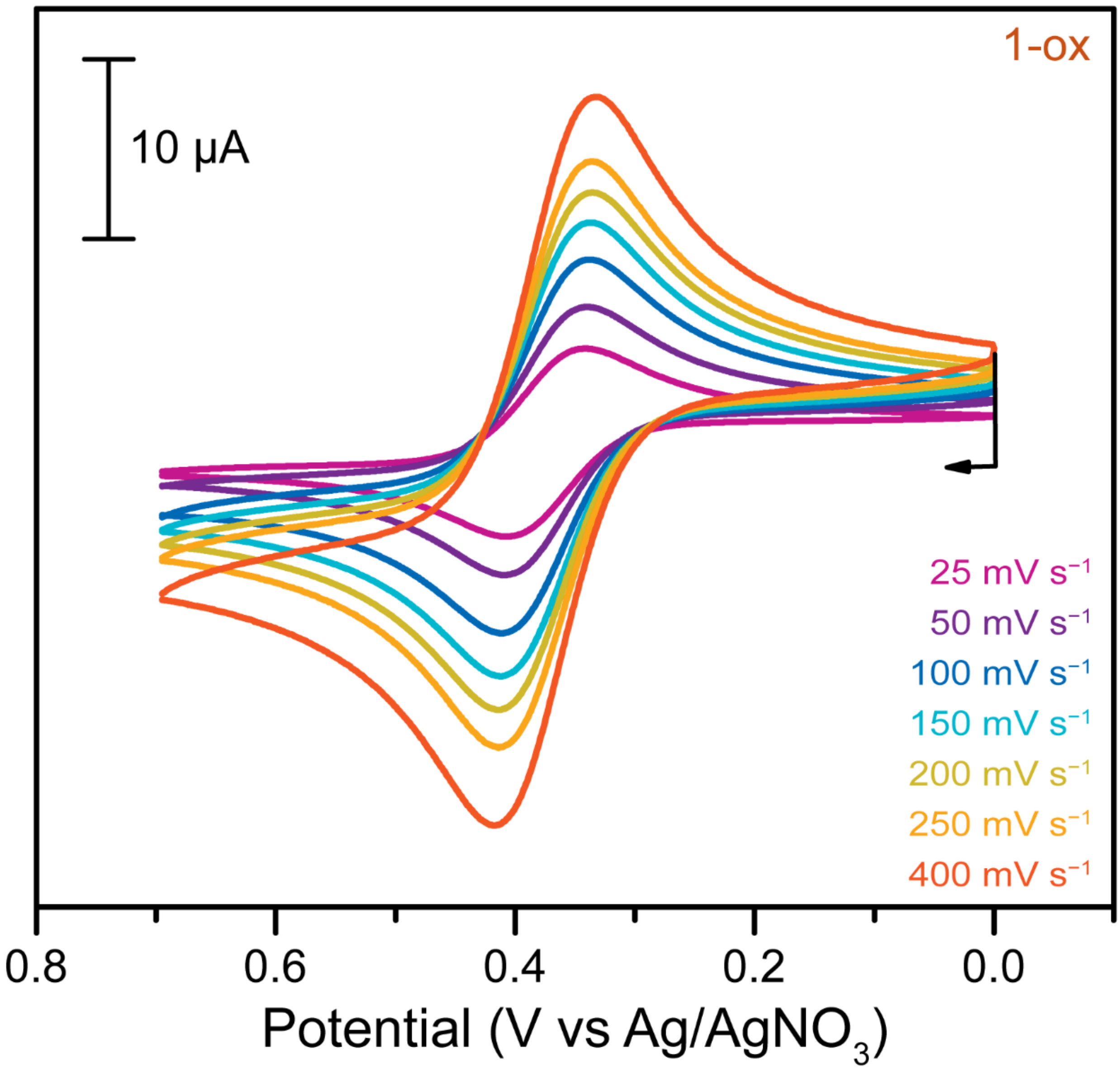

Figure S28. CVs of 0.7 mM of **1-ox** in MeCN containing 0.1 M (^{n}Bu$_4$N)(PF$_6$) supporting electrolyte collected in the potential window around the Fe^{III}_2/$Fe^{II}Fe^{III}$ redox couple at ambient temperature (23–25 °C) using variable scan rates (25–400 mV s^{-1}). The black arrow denotes the scan direction. Glassy carbon, Ag/AgNO$_3$, and Pt mesh were used as working, reference, and counter electrodes, respectively.

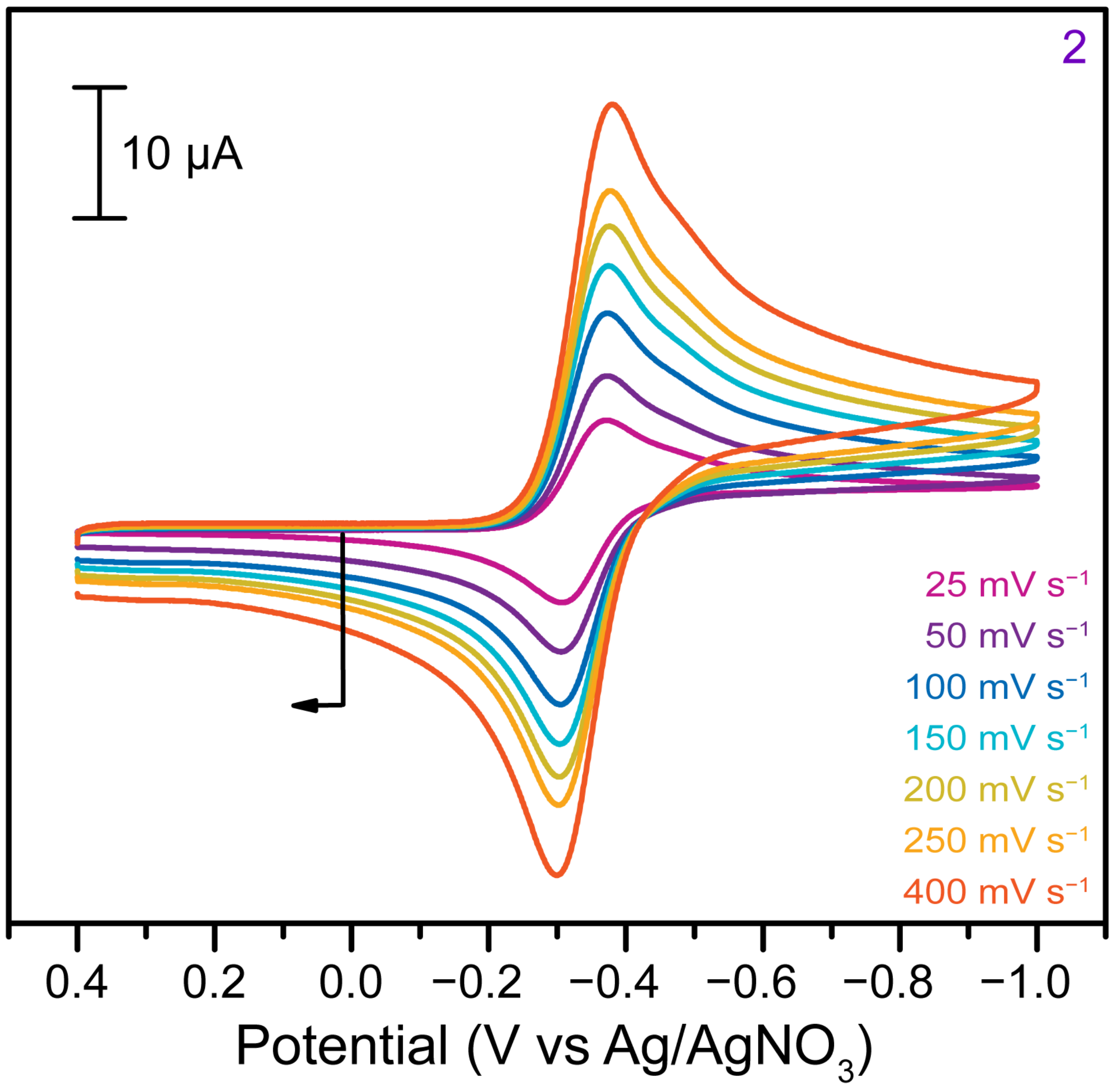

Figure S29. CVs of 0.9 mM of **2** in MeCN containing 0.1 M ($^{n}Bu_4N$)(PF_6) supporting electrolyte collected at ambient temperature (23–25 °C) using variable scan rates (25–400 mV s^{-1}). Vertical black line and arrow denote the open-circuit potential and scan direction, respectively. Glassy carbon, Ag/$AgNO_3$, and Pt mesh were used as working, reference, and counter electrodes, respectively.

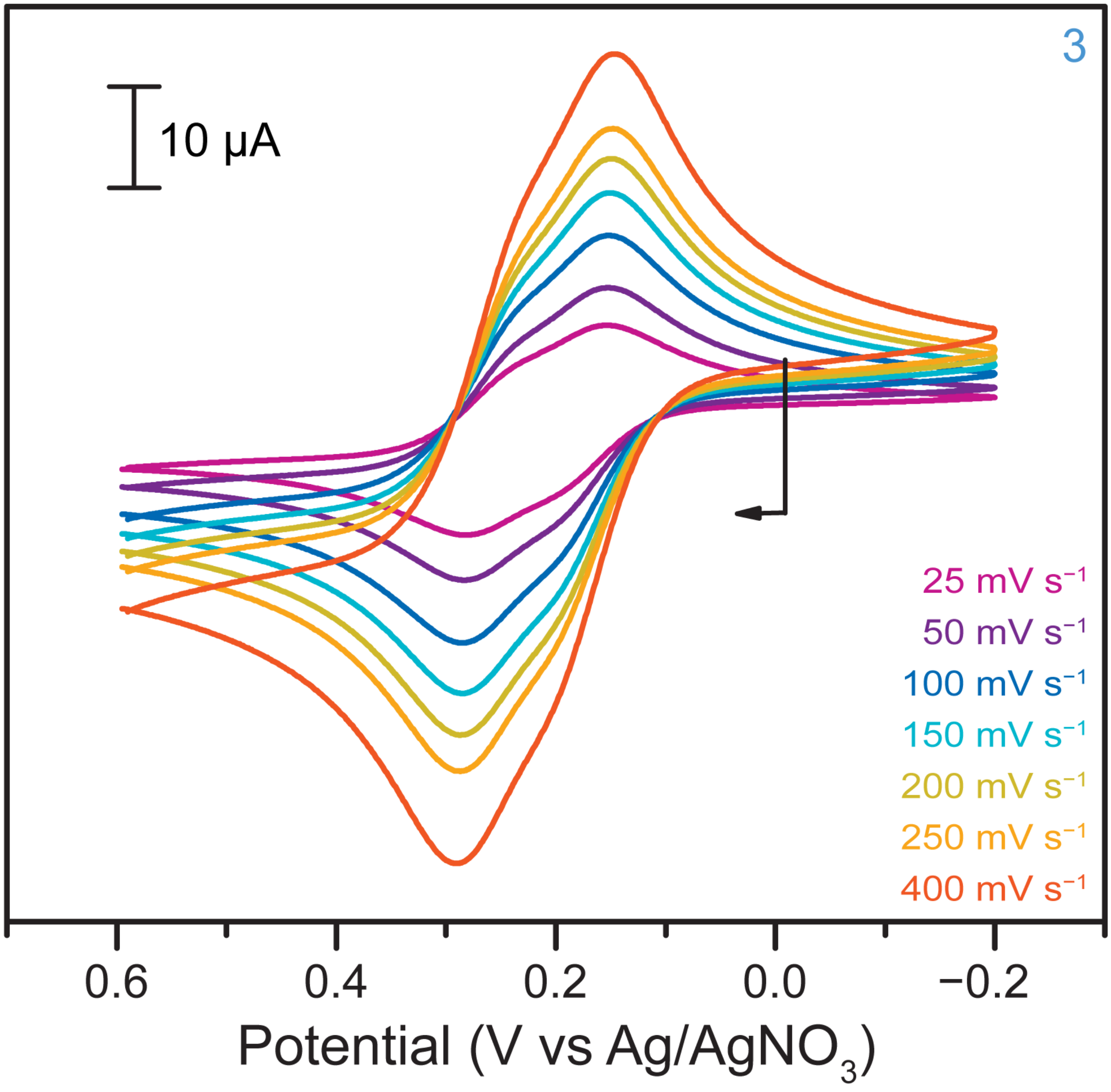

Figure S30. CVs of 0.8 mM of **3** in MeCN containing 0.1 M (^{n}Bu$_4$N)(PF$_6$) supporting electrolyte collected at ambient temperature (23–25 °C) using variable scan rates (25–400 mV s^{-1}). Vertical black line and arrow denote the open-circuit potential and scan direction, respectively. Glassy carbon, Ag/AgNO$_3$, and Pt mesh were used as working, reference, and counter electrodes, respectively.

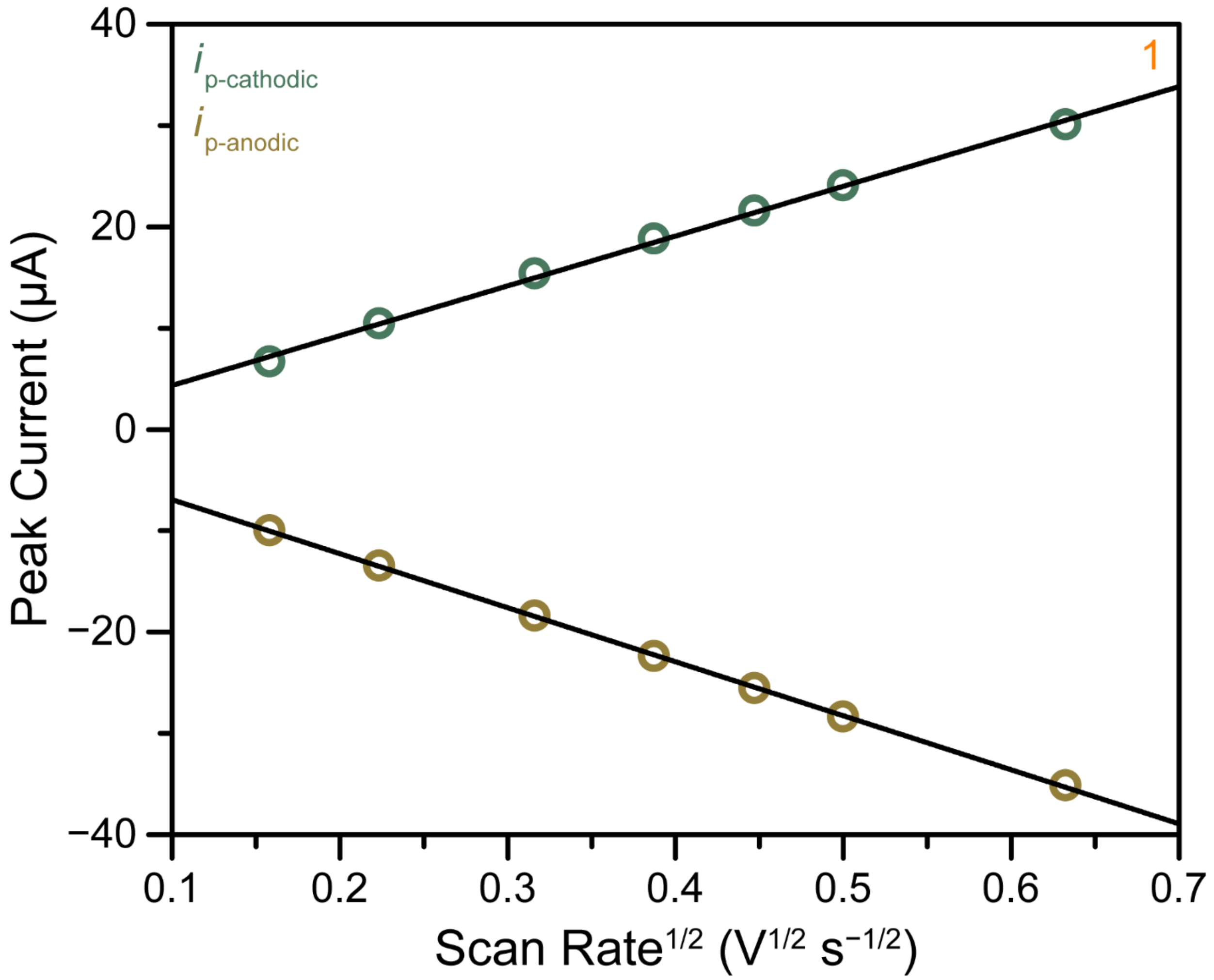

Figure S31. Randles–Ševčík plot for the $Fe^{II}Fe^{III}/Fe^{II}_2$ redox couple of **1** obtained from variable-scan-rate CV data shown in Figure S25. Colored circles denote measured data and black lines correspond to linear fits to the data. $i_{p\text{-cathodic}}$ and $i_{p\text{-anodic}}$ denote the cathodic and anodic peak current, respectively.

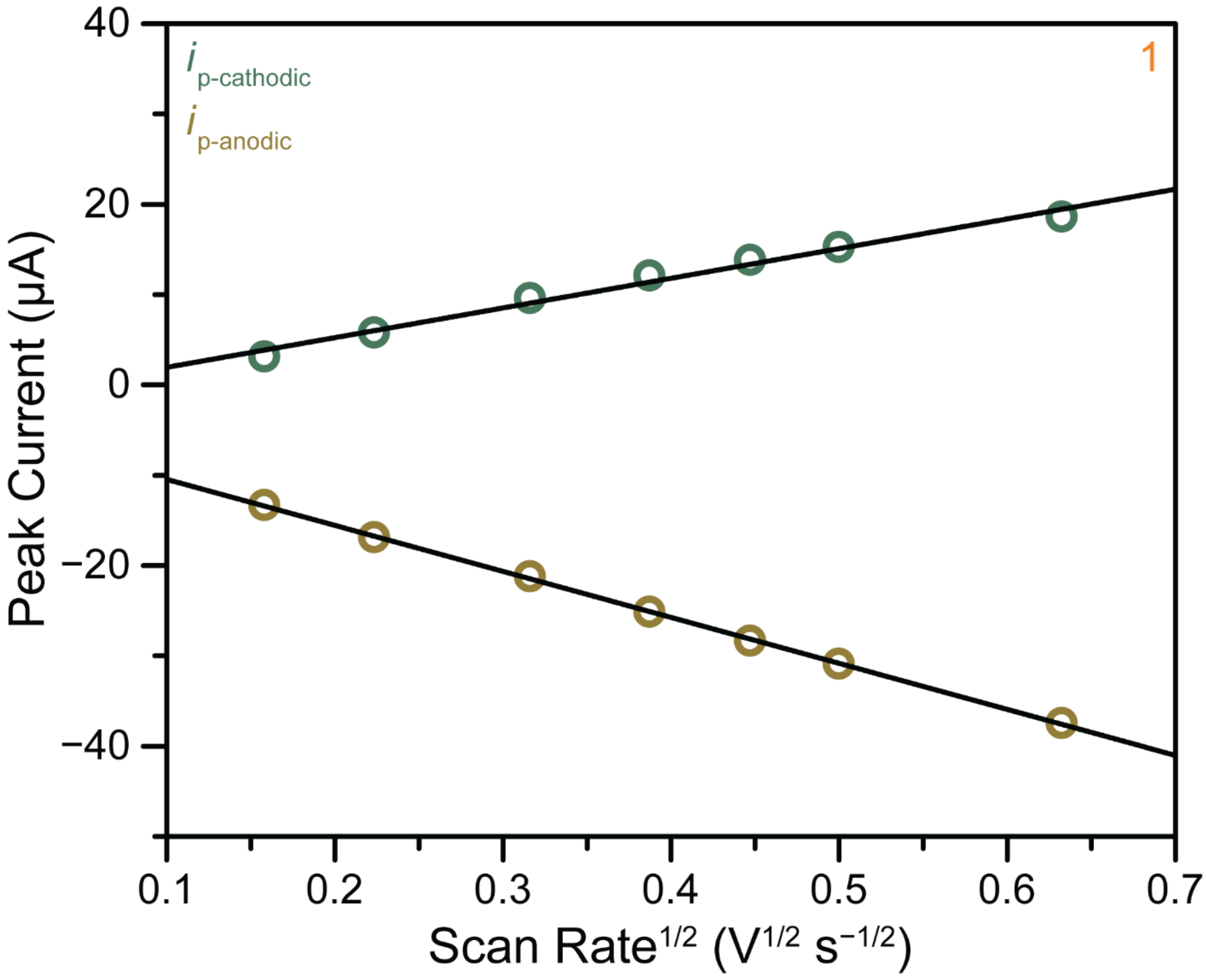

Figure S32. Randles–Ševčík plot for the Fe^{III}_2/$Fe^{II}Fe^{III}$ redox couple of **1** obtained from variable-scan-rate CV data shown in Figure S26. Colored circles denote measured data and black lines correspond to linear fits to the data. $i_{\text{p-cathodic}}$ and $i_{\text{p-anodic}}$ denote the cathodic and anodic peak current, respectively.

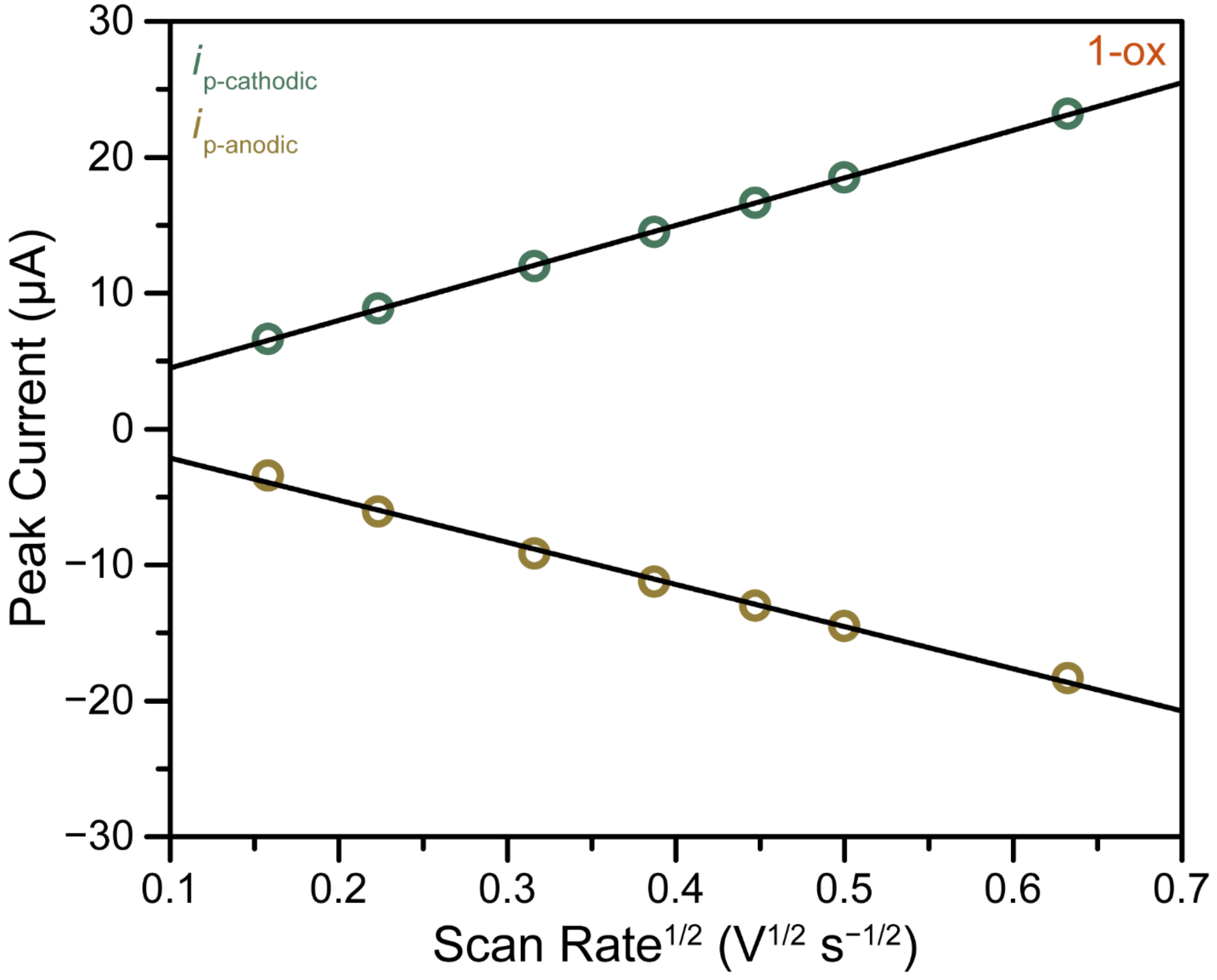

Figure S33. Randles–Ševčík plot for the $Fe^{II}Fe^{III}/Fe^{II}_2$ redox couple of **1-ox** obtained from variable-scan-rate CV data shown in Figure S27. Colored circles denote measured data and black lines correspond to linear fits to the data. $i_{\text{p-cathodic}}$ and $i_{\text{p-anodic}}$ denote the cathodic and anodic peak current, respectively.

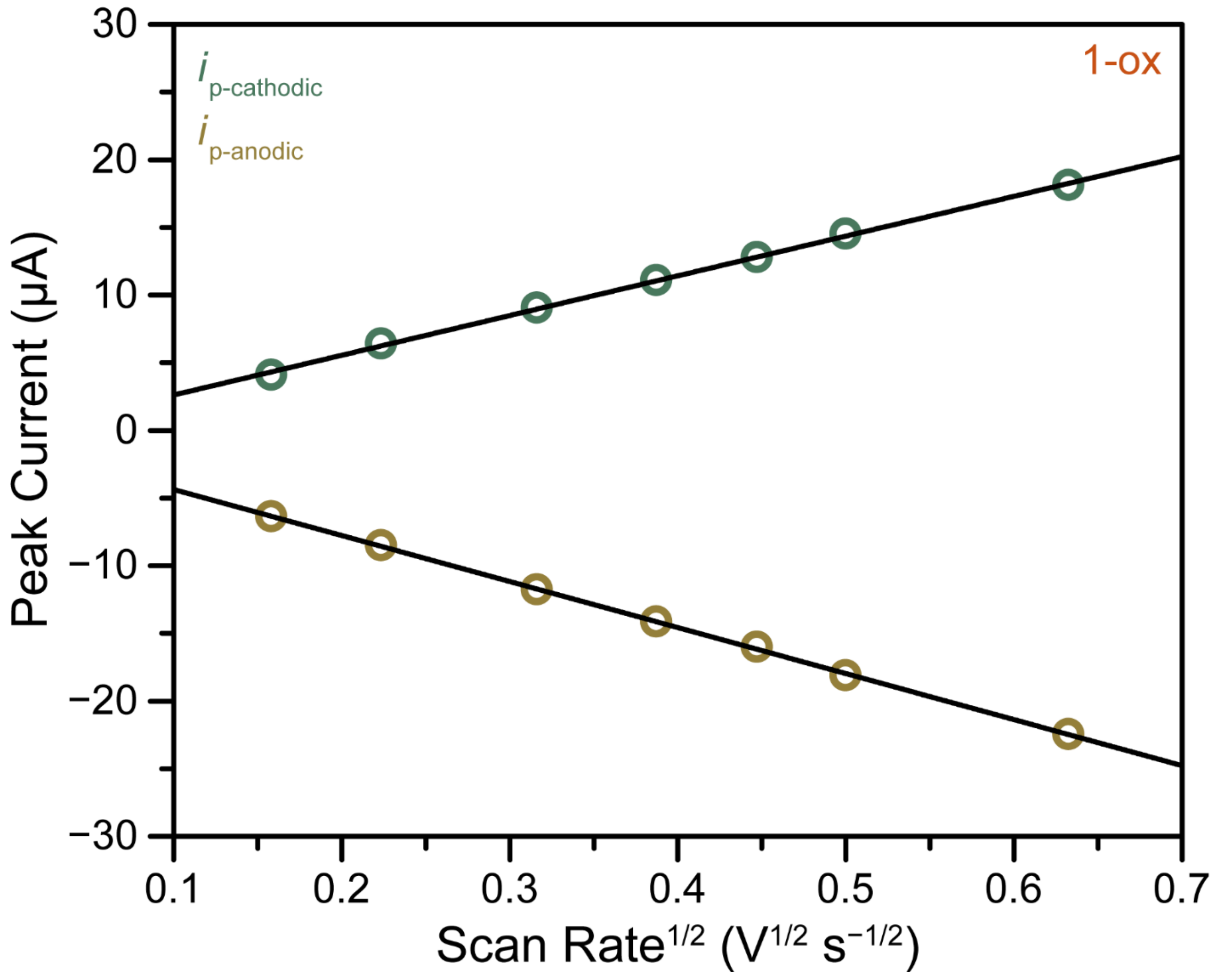

Figure S34. Randles–Ševčík plot for the $Fe^{III}_2/Fe^{II}Fe^{III}$ redox couple of **1-ox** obtained from variable-scan-rate CV data shown in Figure S28. Colored circles denote measured data and black lines correspond to linear fits to the data. $i_{p\text{-cathodic}}$ and $i_{p\text{-anodic}}$ denote the cathodic and anodic peak current, respectively.

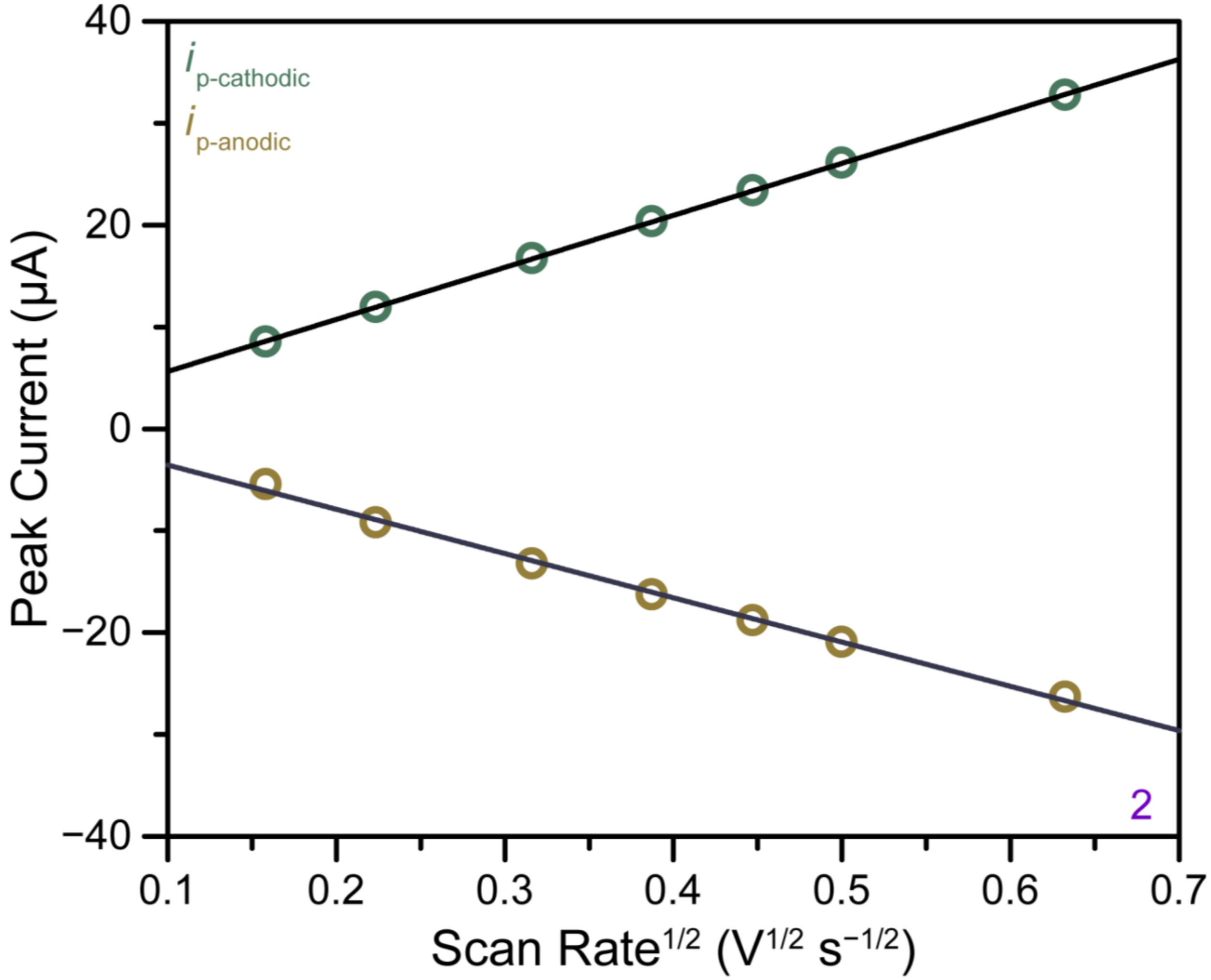

Figure S35. Randles–Ševčík plot for **2** obtained from variable-scan-rate CV data shown in Figure S29. Colored circles denote measured data and black lines correspond to linear fits to the data. $i_{p\text{-cathodic}}$ and $i_{p\text{-anodic}}$ denote the cathodic and anodic peak current, respectively.

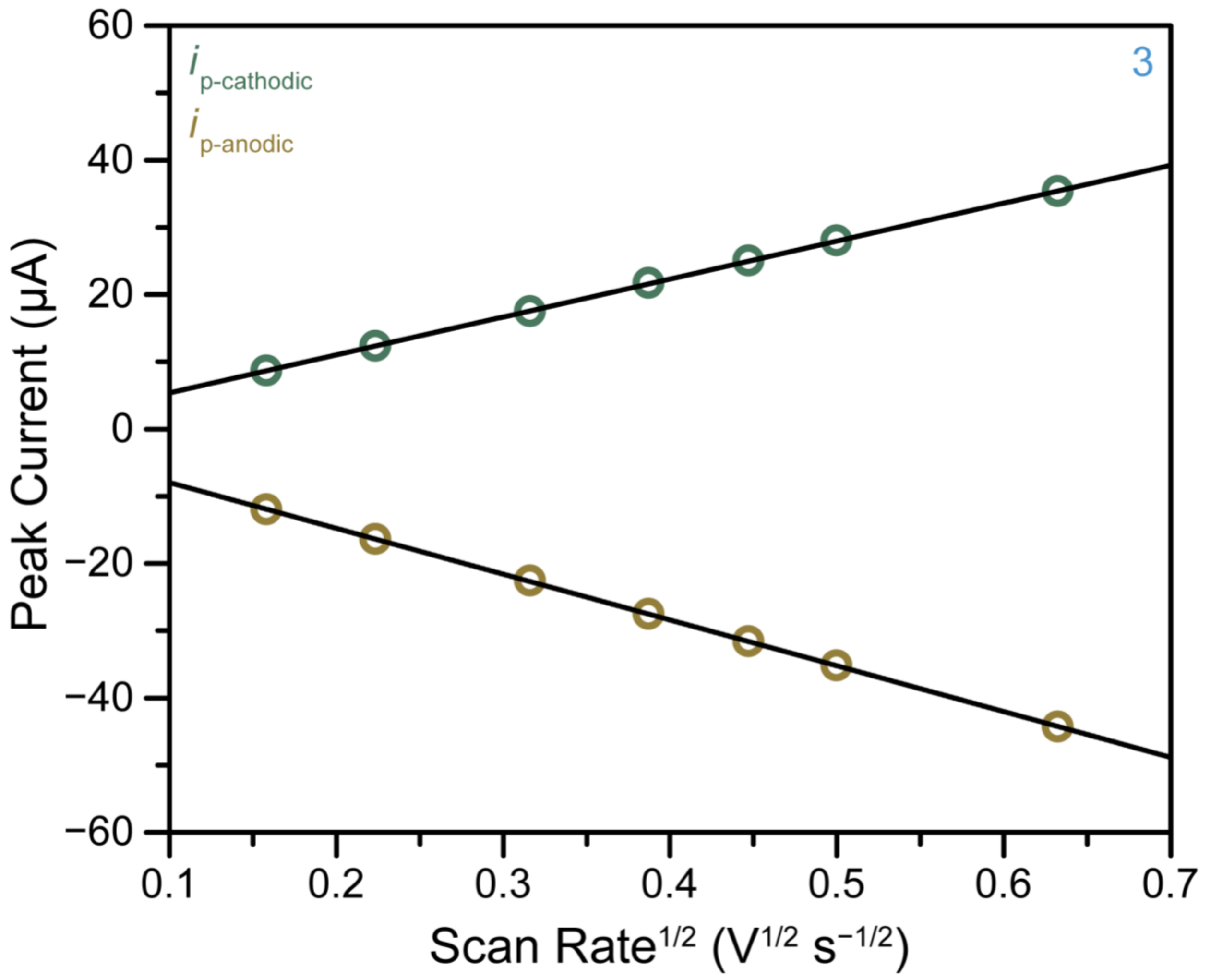

Figure S36. Randles–Ševčík plot for **3** obtained from variable-scan-rate CV data shown in Figure S30. Colored circles denote measured data and black lines correspond to linear fits to the data. $i_{p\text{-cathodic}}$ and $i_{p\text{-anodic}}$ denote the cathodic and anodic peak current, respectively.

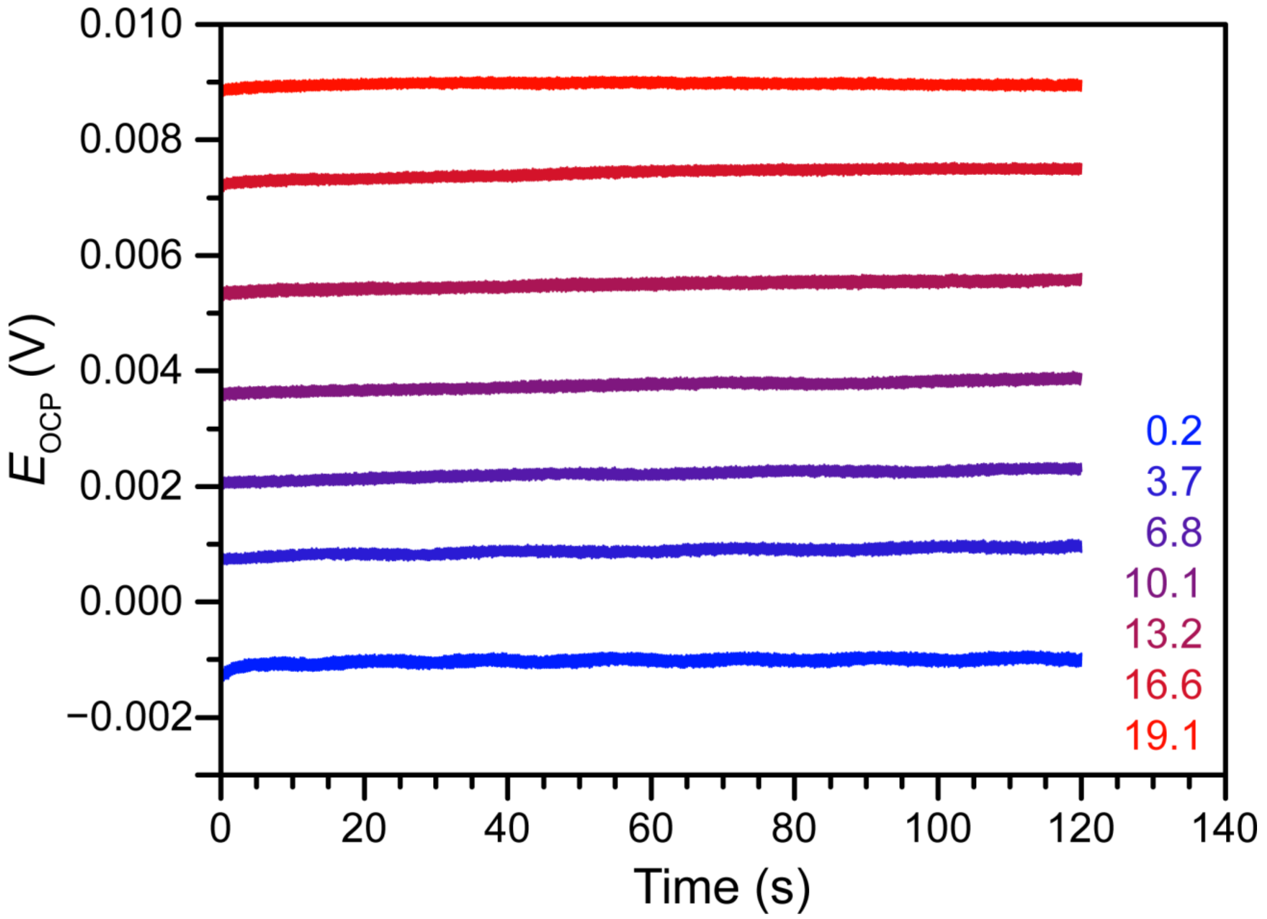

Figure S37. Plot of the potential difference between two $Ag/AgNO_3$ reference electrodes held at different temperatures at open-circuit conditions versus time. Measurements were conducted in MeCN containing 0.1 M ($^{n}Bu_4N$)(PF_6) supporting electrolyte. Colored numbers indicate the differences in temperature between the two electrodes in °C.

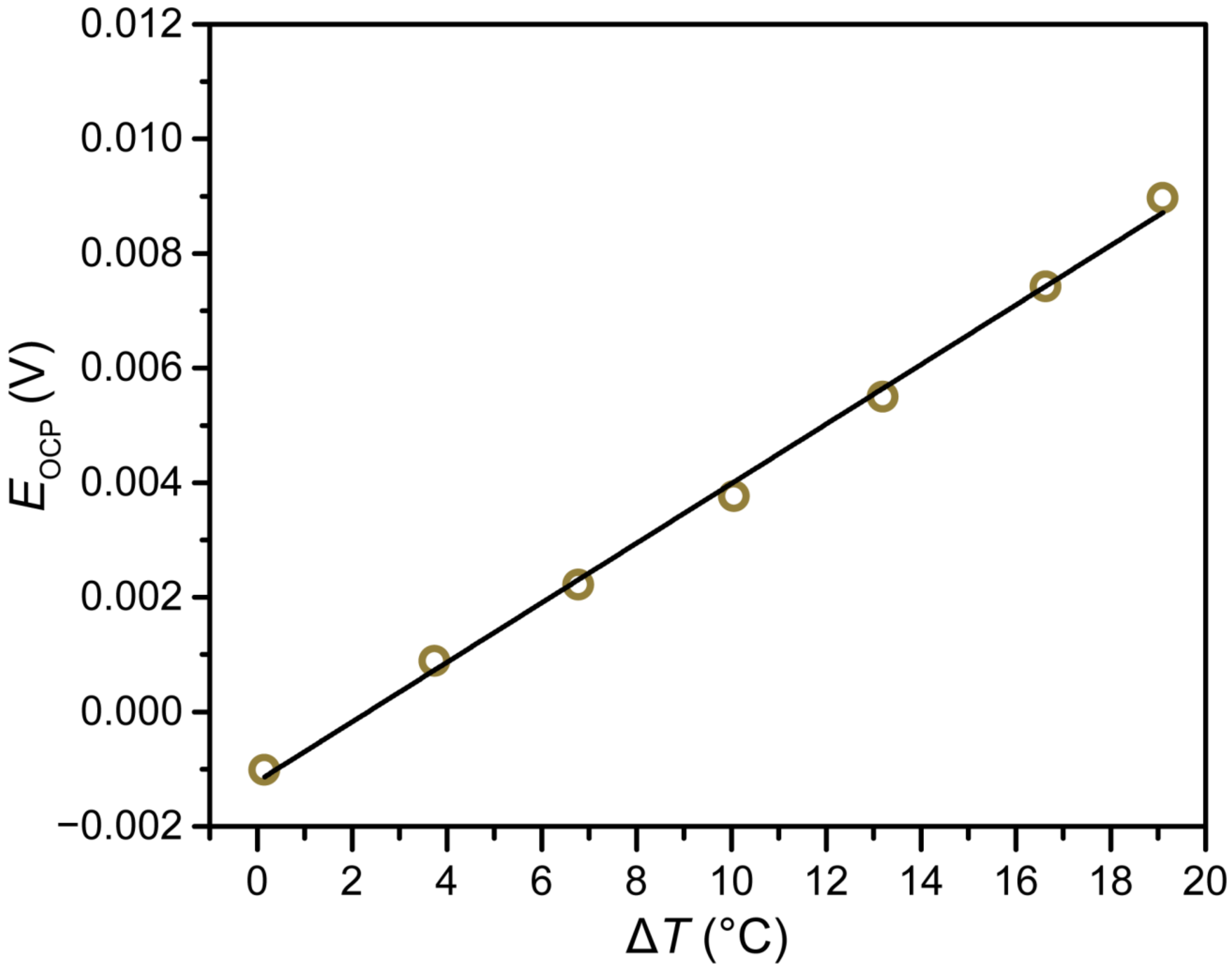

Figure S38. Plot of the potential difference between two Ag/$AgNO_3$ reference electrodes at open-circuit conditions versus temperature difference. Data were obtained from the average potential difference values shown in Figure S37. Colored circles denote experimental data and the black line corresponds to a linear fit to the data. The slope of the linear fit to the data represents the temperature coefficient of the Ag/$AgNO_3$ electrode potential in MeCN containing 0.1 M (^{n}Bu_4N)(PF_6) supporting electrolyte.

D. Supplementary Tables

Table S1. Summary of crystallographic data obtained at 100 K for **1′**, **1-ox′**, **2**, and **3′**.

	**1′**	**1-ox′**	**2**	**3′**
Empirical formula	$C_{37}H_{39}F_6Fe_2N_6O_5P$	$C_{41}H_{49}F_{12}Fe_2N_6O_7P_2$	$C_{37}H_{39}F_{12}FeN_6O_5P_2Zn$	$C_{59.22}H_{57}F_{5.35}Fe_2N_8O_{5.66}P_{0.78}S_{0.22}Zn_2$
Formula wt (g mol^{-1})	904.41	1139.50	1058.90	1346.45
Crystal system	Triclinic	Triclinic	Triclinic	Triclinic
Space group	$P\bar{1}$	$P\bar{1}$	$P\bar{1}$	$P\bar{1}$
Wavelength (Å)	1.54184	1.54184	1.54184	1.54184
Temperature (K)	100.00(10)	99.99(11)	100.00(10)	100.00(10)
a (Å)	10.09790(10)	10.02460(10)	10.05770(10)	10.13145(5)
b (Å)	12.49600(10)	11.56800(10)	11.63200(10)	13.12761(6)
c (Å)	17.61990(10)	21.2391(5)	18.8504(2)	21.59033(10)
α (°)	104.1540(10)	88.5630(10)	79.1970(10)	90.4101(4)
β (°)	91.9900(10)	82.965(2)	81.5880(10)	98.9828(4)
γ (°)	94.2370(10)	77.8730(10)	77.9070(10)	97.9814(4)
V (Å^3)	2146.74(3)	2389.89(7)	2104.98(4)	2807.64(2)
Z	2	2	2	2
ρ_{calcd} (g cm^{-3})	1.399	1.583	1.671	1.593
μ (mm^{-1})	6.408	6.412	5.125	5.971
Reflns coll./unique	71559/9218	101464/15890	65687/8962	96047/12079
$R_{int}{}^{a}$	0.0444	0.040	0.0467	0.0394
R_1 ($I > 2\sigma(I)$)^{b}	0.0328	0.0587	0.0430	0.0273
wR_2 (*all*)^{c}	0.0844	0.1645	0.1086	0.0681
GoF	1.060	1.066	1.127	1.060

$^{a}R_{int} = \Sigma|F_0^2 - \langle F_0^2\rangle| / \Sigma|F_0^2|$. $^{b}R_1 = \Sigma||F_0| - |F_c|| / \Sigma|F_0|$. $^{c}wR_2 = [\Sigma[w(F_0^2 - F_c^2)^2] / \Sigma[w(F_0^2)^2]]^{1/2}$.

Table S2. Selected mean interatomic distances (Å) and angles (°) and octahedral distortion parameter (Σ_{sum}) for the cationic complex in **1-ox′** at 100 K.

	Fe1	Fe2
Fe–$O_{phenoxo}$	1.949(3)	2.103(3)
Fe–$O_{carboxylate}$	1.984(3)	2.086(3)
Fe–$O_{average}$	1.972(2)	2.092(2)
Fe–N	2.166(2)	2.178(2)
Fe⋯Fe[a]	3.410(1)	
Fe–$O_{phenoxo}$–Fe	114.5(2)	
O–C–O	125.2(3)	
trans-O–Fe–N	168.04(8)	169.07(8)
Σ_{sum}[b]	71.08(4)	79.22(4)

[a]Intramolecular Fe⋯Fe distance. [b]Octahedral distortion parameter (Σ) = absolute deviation from 90° of each 12 *cis* angle in [FeN_3O_3].

Table S3. Summary of anodic and cathodic diffusion coefficients of redox couples for **1**, **1-ox**, **2**, and **3** at ambient temperature (23–25 °C), as calculated from Randles–Ševčík analysis for reversible redox couples (eq 2; Figures S31–S36).[a]

Compound	Charge State of Redox Couple	$D_{0\text{-anodic}}$[b] ($cm^2\ s^{-1}$)	$D_{0\text{-cathodic}}$[b] ($cm^2\ s^{-1}$)	$D_{0\text{-average}}$[c] ($cm^2\ s^{-1}$)
**1**	2+/1+	$6.8(4) \times 10^{-6}$	$5.9(3) \times 10^{-6}$	$6.4(3) \times 10^{-6}$
	3+/2+	$6.3(3) \times 10^{-6}$	$2.5(4) \times 10^{-6}$	$4.4(3) \times 10^{-6}$
**1-ox**	2+/1+	$6.0(3) \times 10^{-6}$	$7.5(2) \times 10^{-6}$	$6.8(2) \times 10^{-6}$
	3+/2+	$7.0(1) \times 10^{-6}$	$5.4(2) \times 10^{-6}$	$6.2(2) \times 10^{-6}$
**2**	2+/1+	$4.7(2) \times 10^{-6}$	$6.6(2) \times 10^{-6}$	$5.7(2) \times 10^{-6}$
**3**[d]	3+/1+	$2.1(5) \times 10^{-5}$	$1.4(4) \times 10^{-5}$	$1.8(5) \times 10^{-5}$

[a]A temperature of 25 °C was used for the calculations. [b]The reported value for each redox peak is obtained from the anodic or cathodic peak current using eq 2 (reversible redox couple). Error bars denote the standard deviation of measurements of 2–3 independently prepared samples. [c]Average value of diffusion coefficients obtained using anodic and cathodic peak currents. Error bars were obtained from error propagation. [d]The anodic and cathodic peak currents for the pseudo-two-electron redox wave were each assumed to correspond to a single-electron process (anodic peak current corresponding to oxidation of the second ferrocenecarboxylate ligand and cathodic peak current corresponding to re-reduction of the first ferrocenecarboxylate ligand), thus $n = 1$ was used in eq 2.

Table S4. Summary of anodic and cathodic diffusion coefficients of redox couples for **1**, **1-ox**, **2**, and **3** at ambient temperature (23–25 °C), as calculated from Randles–Ševčík analysis for irreversible redox couples (eq 3; Figures S31–S36).[a]

Compound	Charge State of Redox Couple	$D_{0\text{-anodic}}$[b] ($cm^2\ s^{-1}$)	$D_{0\text{-cathodic}}$[b] ($cm^2\ s^{-1}$)	$D_{0\text{-average}}$[c] ($cm^2\ s^{-1}$)
**1**	2+/1+	$1.1(1) \times 10^{-5}$	$9.6(4) \times 10^{-6}$	$1.0(1) \times 10^{-5}$
	3+/2+	$1.0(1) \times 10^{-5}$	$4.0(6) \times 10^{-6}$	$7.1(6) \times 10^{-6}$
**1-ox**	2+/1+	$9.7(4) \times 10^{-6}$	$1.2(1) \times 10^{-5}$	$1.1(1) \times 10^{-5}$
	3+/2+	$1.1(1) \times 10^{-5}$	$8.7(4) \times 10^{-6}$	$1.0(1) \times 10^{-5}$
**2**	2+/1+	$7.7(3) \times 10^{-6}$	$1.1(1) \times 10^{-5}$	$9.2(6) \times 10^{-6}$
**3**[d]	3+/1+	$3.4(7) \times 10^{-5}$	$2.3(6) \times 10^{-5}$	$2.8(8) \times 10^{-5}$

[a]A temperature of 25 °C was used for the calculations. [b]The reported value for each redox peak is obtained from the anodic or cathodic peak current using eq 3 (irreversible redox couple). Error bars denote the standard deviation of measurements of 2–3 independently prepared samples. [c]Average value of diffusion coefficients obtained using anodic and cathodic peak currents. Error bars were obtained from error propagation. [d]The anodic and cathodic peak currents for the pseudo-two-electron redox wave were each assumed to correspond to a single-electron process (anodic peak current corresponding to oxidation of the second ferrocenecarboxylate ligand and cathodic peak current corresponding to re-reduction of the first ferrocenecarboxylate ligand), thus $n = 1$ was used in eq 3.

Table S5. Comparison of diffusion coefficients for **3** obtained from different analyses.

**Method**	**D_0 (cm^2 s^{-1})**
Randles–Ševčík (eq 2)[a]	$1.8(5) \times 10^{-5}$
Randles–Ševčík (eq 3)[b]	$2.8(8) \times 10^{-5}$
Stokes–Einstein[c]	1.4×10^{-5}
DOSY NMR[d]	$0.9(2) \times 10^{-5}$

[a]A temperature of 25 °C was used for the calculations. Average value of diffusion coefficients obtained from anodic and cathodic peak currents using eq 2 (see Table S3). [b]A temperature of 25 °C was used for the calculations. Average value of diffusion coefficients obtained from anodic and cathodic peak currents using eq 3 (see Table S4). [c]The Stokes–Einstein equation: $D = k_B T/(6\pi\eta r)$, with temperature (T) of 25 °C, dynamic viscosity (η) of 0.346 mPa·s (see ref 4 for the dynamic viscosity of MeCN), and radius of gyration (r) of 4.53 Å (see eq 7; obtained from crystal structure data of **3′** at 100 K) was used for the calculations. [d]Determined in CD_3CN solution at 22–23 °C (see Experimental section for details of other experimental parameters and estimation of error bars).

E. References

(1) Thorarinsdottir, A. E.; Tatro, S. M.; Harris, T. D. Electronic Effects of Ligand Substitution in a Family of Co^{II}_2 PARACEST pH Probes. *Inorg. Chem.* **2018**, *57* (17), 11252–11263.

(2) Bedin, M.; Agarwala, H.; Marx, J.; Schünemann, V.; Ott, S.; Thapper, A. Synthesis and Properties of a Heterobimetallic Iron-Manganese Complex and Its Comparison with Homobimetallic Analogues. *Inorg. Chim. Acta* **2019**, *490*, 254–260.

(3) Hernández-Muñoz, L. S.; González-Fuentes, M. A.; Díaz-Sánchez, B. R.; Fragoso-Soriano, R.; Vázquez-López, C.; González, F. J. Covalent Modification of Carbon Surfaces with Ferrocene Groups through a Self-Mediated Oxidation of Tetrabutylammonium Salts of Ferrocene-Carboxylic Acids. *Electrochim. Acta* **2012**, *63*, 287–294.

(4) Ansari, M.; Singh, S. P. Viscosities and Densities of Acetonitrile-Water Systems at 25 °C. *Res. J. Chem. Sci*. **2022**, *12* (1), 67–69.
